# The Therapeutic Potential of Spirooxindoles in Cancer: A Focus on p53–MDM2 Modulation

**DOI:** 10.3390/ph18020274

**Published:** 2025-02-19

**Authors:** Adel S. Girgis, Yujun Zhao, Angel Nkosi, Nasser S. M. Ismail, Mohamed S. Bekheit, Dalia R. Aboshouk, Marian N. Aziz, M. Adel Youssef, Siva S. Panda

**Affiliations:** 1 Department of Pesticide Chemistry, National Research Centre, Dokki, Giza 12622, Egypt; m_bekheit@yahoo.com (M.S.B.); daliaraslan205@yahoo.com (D.R.A.); mariannady97@yahoo.com (M.N.A.); 2 State Key Laboratory of Drug Research and Small-Molecule Drug Research Center, Shanghai Institute of Materia Medica, Chinese Academy of Sciences, 555 Zuchongzhi Rd, Shanghai 201203, China; yjzhao@simm.ac.cn; 3 Department of Chemistry and Biochemistry, Augusta University, Augusta, GA 30912, USA; ankosi@augusta.edu; 4 Pharmaceutical Chemistry Department, Faculty of Pharmacy, Ain Shams University, Cairo 11566, Egypt; saadnasser2003@yahoo.com; 5 Department of Chemistry, Faculty of Science, Helwan University, Helwan 11795, Egypt; adelgirgis100@gmail.com; 6 Department of Biochemistry and Molecular Biology, Augusta University, Augusta, GA 30912, USA

**Keywords:** spirooxindole, cancer, MDM2, p53

## Abstract

The p53, often referred to as the “guardian of the genome”, is a well-established tumor-suppressor protein that plays a critical role in regulating the cell cycle, DNA repair, differentiation, and apoptosis, with its activity primarily modulated by the MDM2 protein (murine double minute 2, also known as HDM2 in humans). Disrupting the protein-protein interaction between p53 and MDM2 represents a promising therapeutic strategy for developing anticancer agents. Recent studies have shown that several spirooxindole-containing compounds exhibit significant antitumor properties, primarily by inhibiting the p53–MDM2 interaction. This review provides an overview of structure-based spirooxindoles that could have therapeutic potential. It highlights findings from the past decade concerning their antiproliferative properties and implications for interfering with the p53–MDM2 interaction. The discussion includes various analogs of spirooxindoles as promising candidates for optimizing leads in drug discovery programs aimed at developing novel and clinically effective agents.

## 1. Introduction

The human biological system develops a self-destructive pathway to maintain physiological homeostasis and defend itself against harmful substances in the environment by eliminating unwanted cells. This process is known as regulated cell death (RCD) or programmed cell death (PCD). RCD is governed by signaling pathways that play a crucial role in tissue renewal [1,2]. A natural balance between proliferation and RCD occurs in normal cells, but this balance is disrupted in cancer cells. Many RCD pathways fail to function properly in cancer cells, allowing genetic abnormalities to persist and contributing to drug resistance and recurrence, which are significant challenges in cancer treatment. Numerous subtypes of RCD have been identified, including necroptosis, pyroptosis, parthanatos, ferroptosis, cuproptosis, autophagy-dependent cell death, and apoptosis, all of which may influence cancer progression and treatment [2,3].

Apoptosis has a pivotal role in the pathogenesis of many diseases, either through hyperactivation, as seen in neurodegenerative diseases and immunodeficiency, or through suppression, as in cancer and autoimmune disorders. Therefore, regulating apoptotic signaling pathways presents a promising approach for treating various diseases [4]. Inducing cancer cell death by targeting apoptotic signaling pathways through upregulating TNFR (tumor necrosis factor receptor), p53 (tumor-suppressor gene), Apaf-1 (apoptotic peptidase activating factor 1), and Cyt-c (cytochrome complex), or downregulating BCL2 (B-cell lymphoma-2) and NF-κB (nuclear factor kappa-light-chain-enhancer of activated B cells), is a promising strategy for combating cancer [1].

The p53 protein, encoded by the TP53 gene, is a well-known tumor suppressor critical in regulating the cell cycle, DNA repair, differentiation, and apoptosis [5,6]. One of its main functions is maintaining genomic stability and preventing mutations during cell division, thereby inhibiting cancer development by arresting the cell cycle or inducing apoptosis. P53 has a short life span and is regulated by the MDM2 protein [6]. In about 50% of cancer types, the p53 protein is mutated and rendered inactive. In other cancer types, the overexpression of MDM2 negatively regulates the wild-type p53. Disrupting the interaction between p53 and MDM2 has emerged as a promising therapeutic strategy for developing new anticancer agents. Inhibiting MDM2 facilitates the release and activation of p53, enabling it to control cancer progression [7,8]. X-ray studies have shown that the interaction between p53 and MDM2 is primarily mediated by a specific set of amino acids (Phe19, Trp23, and Leu26) located in the N-terminal α-helix of p53. These amino acids can dock and fit into a specific hydrophobic pocket on MDM2 [9,10].

**Figure 1:**
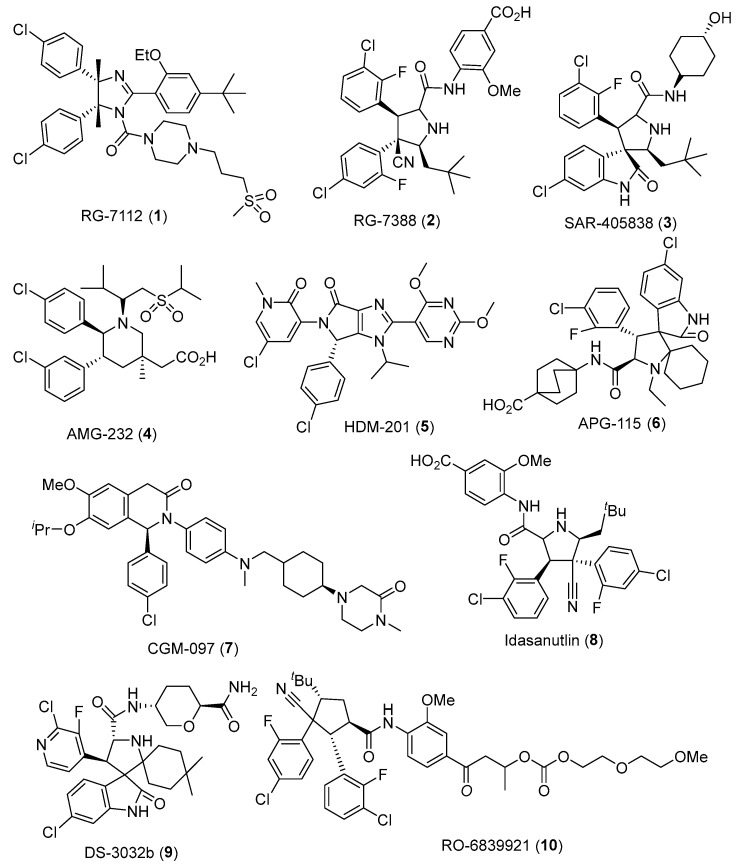
Compounds entered in clinical trials as p53–MDM2 inhibitors.

Several chemical scaffolds have shown significant inhibitory properties against p53–MDM2, including spirooxindole, imidazole, benzodiazepinedione, chalcone, isoquinoline, piperidone, and morpholinone [11,12]. Figure 1, compounds **1**–**10**, illustrates representative compounds that have progressed to human clinical trials as p53-MDM2 inhibitors [12,13,14].

**Figure 2:**
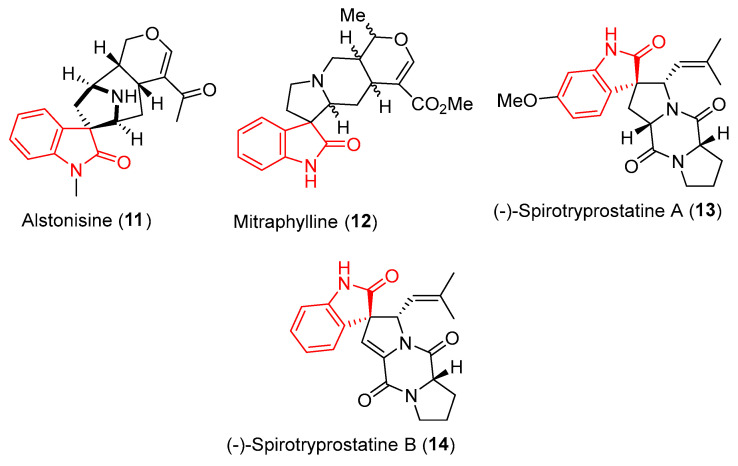
Natural spirooxindole-containing compounds with antiproliferation properties.

**Figure 3:**
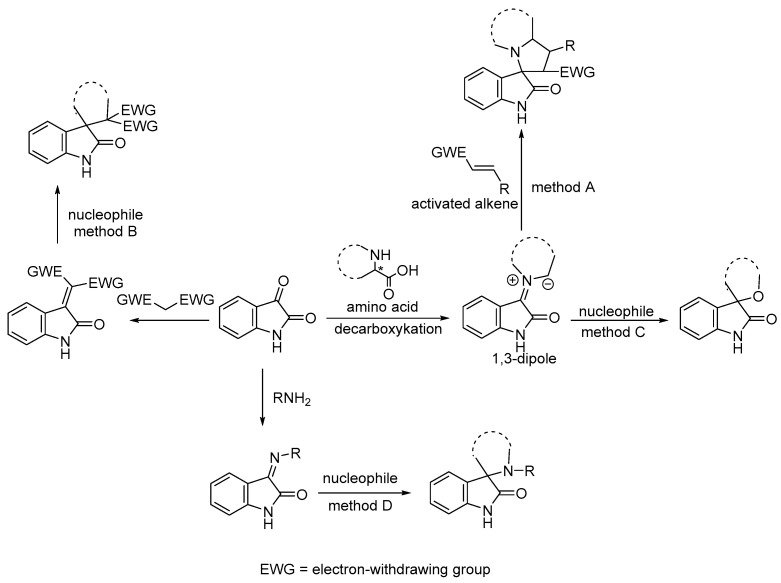
Some synthetic methodologies for spirooxindoles.

There has been growing interest in spirooxindoles due to the diverse and promising biological properties exhibited by their analogs. These compounds have shown potential as anticancer agents [15,16,17], inhibitors of SARS-CoV-2 [18,19], and cholinesterase inhibitors beneficial for Alzheimer’s disease [20], as well as demonstrating antibacterial and antifungal activity [16,21,22,23,24,25]. Additionally, some natural spirooxindole-containing compounds have been isolated and characterized with promising antiproliferative properties against various cancer types [26,27] (Figure 2 reveals some representative analogs **11**–**14**). Several synthetic methodologies for spirooxindoles have been reported [28]. These include: (a) Dipolar cycloaddition ([3+2]-cycloaddition), which typically occurs between compounds containing unsaturated linkages (dipolarophiles) and dipoles. The regio- and stereoselectivity of these reactions have been discussed [29,30,31,32]; (b) Domino Knoevenagel–Michael cyclization, which can occur between nucleophilic active methylene-containing compounds and the reactive C-3 of isatin(s), yielding the Knoevenagel alkenes that can act as potentially active Michael acceptors in further Michael reactions with cyclization; (c) Michael cyclization of isatin(s), which relies on the Michael reaction of the isatin carbonyl group at C-3, along with cyclization determined by the accessibility of the chemical structure of the attacking Michael agent; (d) Aminalization of isatin C-3, where the formed aminals can undergo cyclization reactions to yield spirooxindoles (Figure 3).

This article reviews structure-based spirooxindoles that feature various heterocycles with potential therapeutic relevance. To gather recent publications from the last decade, multiple search engines were utilized, including PubChem, Web of Science, Scopus, and ScienceDirect. The study highlights key publications showcasing the antiproliferative properties of these compounds and their interactions with p53–MDM2, supported by standard techniques. This work benefits the scientific community and aims to assist researchers in developing and optimizing new compounds that may be useful in treating various types of cancer [28,33,34,35,36,37]. While some articles have discussed similar topics, this study focuses on recent findings and provides a comprehensive overview.

Cancer remains one of the most significant health challenges globally, with GLOBOCAN statistics showing that it affects millions of people each year [38,39]. Despite the development and clinical approval of numerous targeted therapies and multi-targeted drugs, several cancer types continue to be aggressive, particularly in advanced stages. This leads to many cases becoming incurable, causing significant pain and mortality for patients [38].

The current study examines various spirooxindole-containing compounds that demonstrate promising biological properties. These findings are supported by established techniques that can be utilized to optimize more potent analogs. Furthermore, different methods, including computational studies, can aid in identifying new promising compounds based on existing bioactive analogs.

## 2. Drug Candidates of p53–MDM2 Inhibitory Properties

Although blocking the p53–MDM2 pathway is an effective approach for many cancer types, no drugs targeting this pathway have been approved for use. However, several drug candidates with impressive efficacy have been identified and are progressing into later-stage clinical trials in anticipation of their approval as accessible anticancer drugs [13].

### 2.1. Nutlins

**Figure 4:**
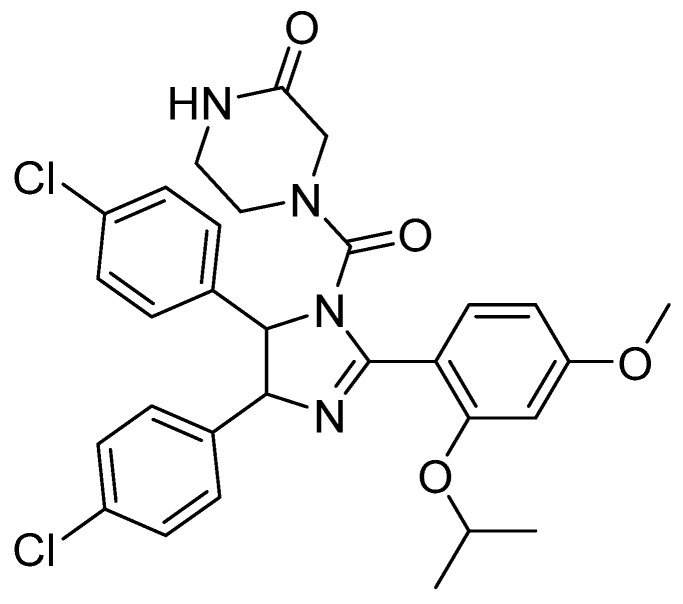
Nutlin-3 (**15**, p53–MDM2 inhibitor).

Nutlins are a group of imidazoline analogs identified as inhibitors of p53–MDM2 interactions. Several analogs from this family were examined; however, nutlin-3 **15** (Figure 4) emerged as the most promising candidate of growing interest [40]. Numerous preclinical studies indicated its oral availability as a therapeutic anticancer agent. Few side effects (e.g., weight loss) were noted in preclinical trials. Significant efficacy was observed against colon (HCT116) and PC3 (prostate) cancer cell lines; however, no clinical studies on **15** have been reported. This may be due to ongoing efforts towards optimizing agents, such as **1**, which have enhanced potency derived from the same imidazolinyl family [13].

### 2.2. RG-7112

RG-7112 (**1**, Figure 1) is an imidazoline-based analog from the nutlin family, developed by Roche. It has entered phase I clinical studies, showing significant properties against solid tumors and leukemia. Additionally, promising efficacy was noted against the HCT116 cell line (MTT assay) [13,41].

### 2.3. SAR405838 (MI-77301)

SAR405838 (MI-77301) (**3**, Figure 1) revealed *K_i_* = 0.88 nM towards human MDM2 protein with higher potency relative to that of nutlin-3a (>50 times). Its ability to activate wild p53 type in vitro and tissue xenograft cancers of solid tumors and leukemia was reported [42]. In vivo studies of mouse xenograft models (osteosarcoma, acute leukemia, prostate, colon, and dedifferentiated liposarcoma cancers) demonstrated recognizable or complete cancer inhibition [43,44]. Phase I studies (26 patients with advanced or metastatic solid tumors and wild p53 type receiving 200 mg of SAR405838 once daily combined with pimasertib 45 mg twice daily, orally) evidenced the potential therapeutic effect of SAR405838 and its safety profile [44].

### 2.4. APG-115

APG-115 (**6**, Figure 1) is a potent MDM2 inhibitor supported by clinical studies involving patients with solid tumors. Potential activity was observed against acute myeloid leukemia, as demonstrated by in vivo animal model studies. The antileukemic activity of APG-115 was regarded as a therapeutic single agent. A positive effect was also noted when APG-115 was combined with a synergistic agent [45]. Phase 1 studies showed similar observations in patients with solid tumors [46]. An enhancement of the antitumor effect (gastric adenocarcinoma) was reported when combining APG-115 with radiotherapy [47].

### 2.5. Idasanutlin (RG-7388)

Idasanutlin (RG-7388, **8**, Figure 1) is a pyrrolidinyl analog that has entered phase III clinical trials (Roche Company) for treating acute myeloid leukemia with the DNA polymerase inhibitor cytarabine. A combination of ixazomib citrate and dexamethasone was also investigated in phase I/II clinical trials for multiple myeloma and phase I trials for non-Hodgkin lymphoma [13,48,49].

### 2.6. DS-3032b (Milademetan)

Milademetan (DS-3032b, **9**, Figure 1) is a well-established TP53 activator that induces cell cycle arrest (G1 phase) and apoptosis in neuroblastoma cell lines. In vivo, studies with nude mice demonstrated tumor growth inhibition and extended survival following oral administration of 50 mg/kg body weight for 50 days, with the treatment given 4 days on and 2 days off, respectively [50]. A phase I clinical trial involving 16 adult patients showed the safety and efficacy of DS-3032b in combination with low doses of other therapies (cytarabine ± venetoclax) for adult patients with wild-type TP53 acute myeloid leukemia. Recognizable gastrointestinal toxicity was observed during the study [51].

## 3. Synthetic Spirooxindole with Various Heterocycles of p53–MDM2 Inhibitory Properties

### 3.1. Spirooxindole Combined with Five-Membered Heterocycle

#### 3.1.1. Spirooxindole–Pyrrolidine

**Scheme 1:**
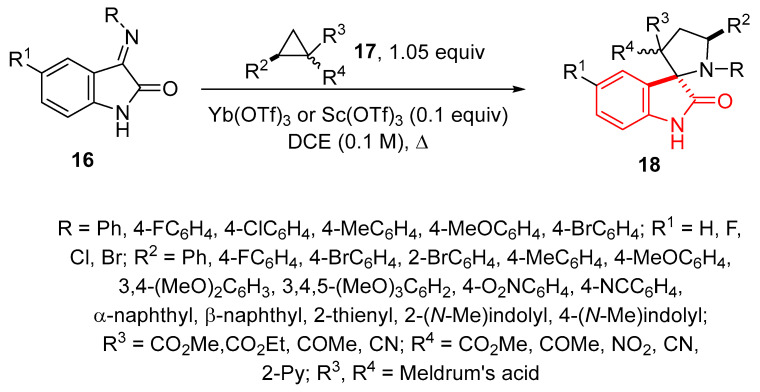
Synthesis of spiro[oxindole-3,2′-pyrrolidines] **18**.

Spiro[oxindole-3,2′-pyrrolidines] **18** were synthesized through a stereo-controlled [3+2]-cycloaddition reaction of donor-acceptor 1,1-disubstituted cyclopropanes **17** with 3-imino-2-indolinones **16** in refluxing dichloroethane (DCE) in the presence of Lewis acid [Yb(OTf)_3_ “trifluoromethanesulfonic acid ytterbium(III) salt” or Sc(OTf)_3_ “trifluoromethanesulfonic acid scandium(III) salt”) (Scheme 1). The reaction was reported to be diastereoselective, affording the trans-isomer [diastereomeric ratio (dr) 60:40–95:5]. Some of the synthesized agents exhibited considerable antiproliferative properties (MTT assay) against pancreatic PC-3 (p53−) and LNCaP (p53+) cancer cell lines. The most promising was the compound with R = Ph, R^1^ = H, R^2^ = Ph, R^3^ = CO_2_Me, R^4^ = NO_2_ relative to nutlin-3a (IC_50_ = 6.8–8.5/28.1–29.6, 6.2–7.9/2.4–2.8 μM against PC-3 (p53−) and LNCaP (p53+), respectively] (Supplementary Figure S1 shows the synthesized compounds with diastereomeric ratio values and biological properties) [52].

**Scheme 2:**
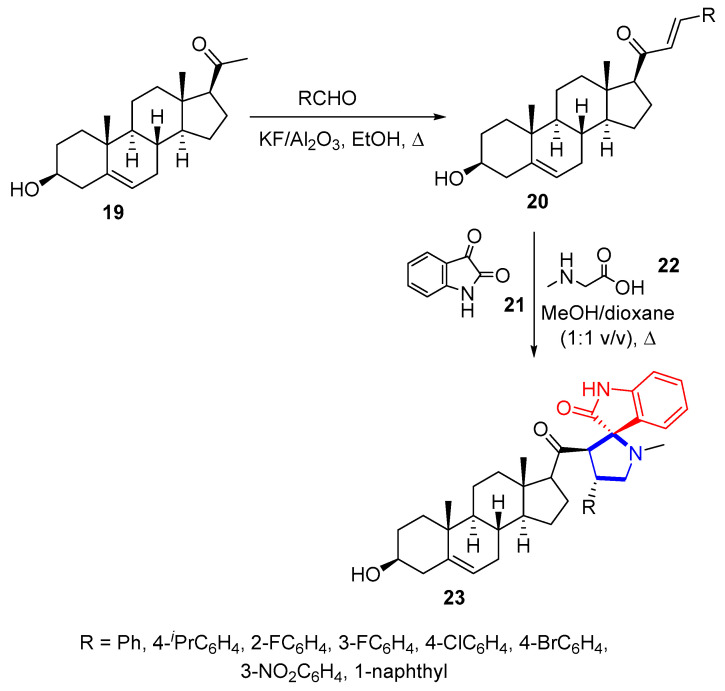
Synthesis of stereoidal spirooxindoles **23**.

Spirooxindole linked to a steroid scaffold was designed and synthesized through the reaction of 21-arylidenepregnenolones **20** (prepared from pregnenolone **19** and aryl aldehyde) with unstabilized 1,3-dipole azomethine ylide formed during the in situ condensation of isatin **21** and sarcosine **22** in refluxing methanol/1,4-dioxane (1:1 *v*/*v*), resulting in the targeted steroidal spirooxindoles **23** (Scheme 2). Considerable antiproliferative properties (MTT assay) against T24 (urinary bladder), SMMC-7721 (hepatocarcinoma), MCF7 (breast), and MGC-803 (gastric) cancer cell lines were observed in some of the synthesized agents. The most promising were those with R = 2-FC_6_H_4_ and 3-FC_6_H_4_ compared to 5-fluorouracil (standard reference/drug) (Supplementary Figure S2). This directs attention to the role of fluorine substitution in enhancing the observed antiproliferation properties. Cytofluorimetric analysis of the synthesized agent with R = 3-FC_6_H_4_, showing potent antiproliferative properties against MCF7 compared to the standard drug (IC_50_ = 4.6 ± 0.7, 10.5 ± 1.6 μM, respectively), indicated that the synthesized analog is an apoptosis inducer with concentration-dependent behavior. Cell cycle arrest was also noted at G2/M, with a significant decrease in cell numbers in other cell cycle phases. Molecular docking studies (PDB: 1RV1, AutoDock Vina software) were conducted to elucidate the mechanism underlying the activity of the most promising agent discovered (R = 3-FC_6_H_4_) against MDM2, revealing two hydrogen bonds with the Lys94 and Thr101 residues of the protein active site [53].

**Scheme 3:**
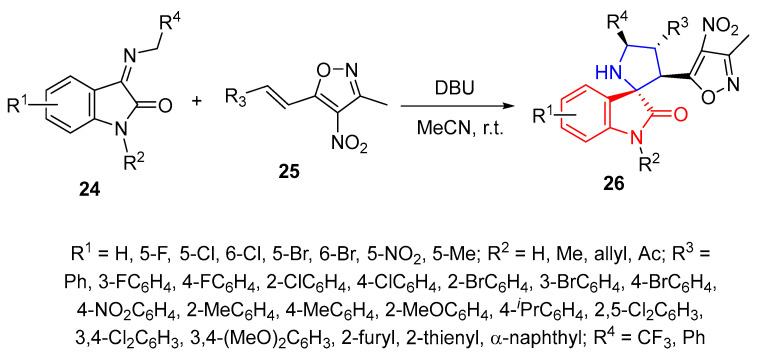
Synthesis of spirooxindoles bearing isoxazol-5-yl heterocyclic scaffold **26**.

Spirooxindoles featuring an isoxazole-5-yl heterocyclic scaffold **26** were synthesized through a reaction between isatin-imines **24** and 5-styryl isoxazoles **25** in acetonitrile, employing a catalytic amount of DBU (1,8-diazabicyclo[5.4.0]undec-7-ene) at room temperature (r.t.) (Scheme 3). Many of the synthesized compounds exhibited promising activity, with the most potent ones displaying the following configurations: R^1^ = 6-Cl/6-Br, R^2^ = H/H, R^3^ = Ph/Ph, and R^4^ = CF_3_/CF_3_, with Ki values of 0.24 ± 0.06, 0.26 ± 0.05, and 0.28 ± 0.0 μM, respectively, against MDM2. The potent agents demonstrated a high capability of inducing ferroptosis, which was assessed through cytotoxicity assays using the p53-wild-type MCF7 (breast cancer) cell line and the addition of ferrostatin-1 (Fer-1). The IC_50_ values for these compounds were 0.12 and 0.17 μM in the absence of Fer-1, and 13.5 and 13.7 μM in its presence. In xenograft models using Balb/c nude mice bearing breast cancer, the promising agent (with R^1^ = 6-Cl, R^2^ = H, R^3^ = Ph, and R^4^ = CF_3_) was administered at doses of 25 or 50 mg/kg/day via intraperitoneal injection for 17 days, further supporting the observed efficacy. Additionally, molecular modeling and docking studies (utilizing the CDOCKER module of the Discovery Studio 3.5 package) reinforced the bio-properties exhibited by the promising agent. These studies revealed hydrogen bonding between the indolyl NH and LEU54 and two π-π stacking interactions between the isoxazolyl and phenyl rings with HIS96 in the protein’s active pocket. Supplementary Figure S3 summarizes the bio-properties of the synthesized agents [54].

**Scheme 4:**
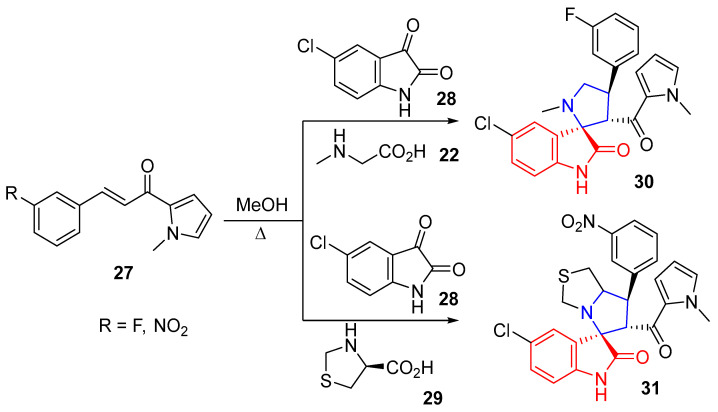
Synthesis of spirooxindoles linked to pyrrole heterocycle through a carbonyl function/spacer **30** and **31**.

**Figure 5:**
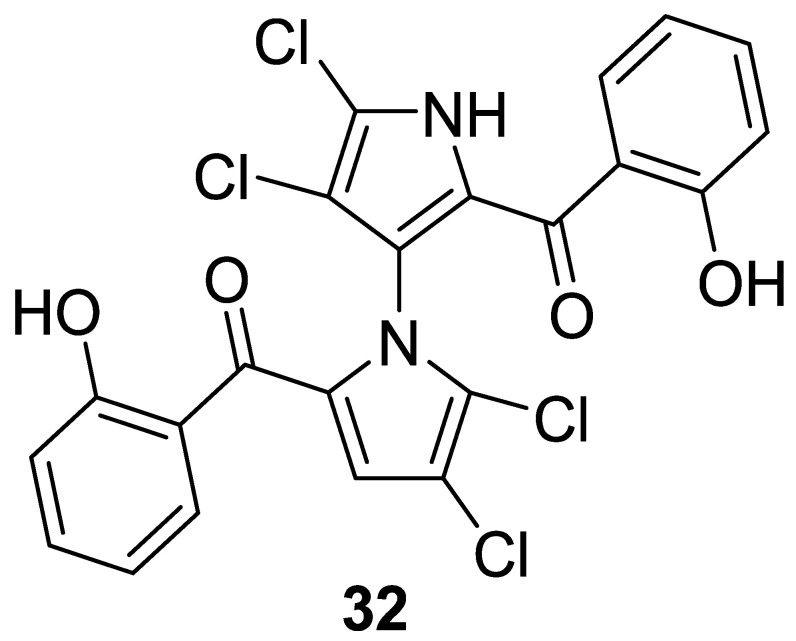
Chemical structure of marinopyrrole A **32**.

Spirooxindoles were attached to the pyrrolyl heterocycle via a carbonyl function/spacer **30** and **31**, prepared through a [3+2]-cycloaddition of 3-aryl-1-(1-methyl-1*H*-pyrrol-2-yl)-2-propen-1-ones **27** with azomethine ylides derived from 5-chloroisatin **28** and α-amino acids (sarcosine **22** or thioproline **29**) in refluxing MeOH (Scheme 4). The promising properties of the spiro-scaffold inspired the design of the targeted hybrid agents as an MDM2 inhibitor and the BCL2 (B-cell lymphoma-2) inhibitory properties of the pyrrolyl heterocycle, which is derived from marinopyrrole A **32** (a natural compound with potent BCL2 inhibition) (Figure 5). Single crystal X-ray studies confirmed the stereochemical structures of **30** and **31**. A possible cycloaddition mechanism was proposed based on quantum chemical calculations [DFT, B3LYP/6-31G(d)]. Compound **31** demonstrated greater potency than **30** in antiproliferation activity screening (MTT assay) against MDA-MB231 (breast), HepG-2 (liver), and Caco-2 (colon) carcinoma cell lines, relative to 5-fluorouracil (IC_50_ = 0.7424 ± 0.0597/33.5660 ± 4.5720/6.2330 ± 1.4650, 0.0018 ± 0.0004/0.0569 ± 0.0020/0.0028 ± 0.0020, 7.0500 ± 0.2040/4.8290 ± 0.2960/1.0480 ± 0.1560 μM against the tested cell lines for **30**, **31**, and 5-fluorouracil, respectively). Safety behavior was observed regarding Wi-38 (normal lung fibroblasts), supporting the selectivity profile against non-cancerous or normal cells. Similar findings were noted from flow cytometric studies demonstrating the apoptosis activity of **31**, which was more pronounced than that of compound **30**. The p53 immunohistochemical studies revealed a higher transactivation in HepG2-treated cells with compound **31** compared to the analog **30**. Both synthesized agents showed equally relative changes in BCl2 expression in the treated HepG2 assay (qRT-PCR assay, 1.25-fold downregulation). Molecular docking studies (PDB: 5LAW, MOE 2016.0802 software) illustrated the MDM2 inhibitory properties of the synthesized agents. Indolyl NH formed hydrogen bonds with LEU54, as noted during the docking analysis, with a docking score of **30** in the protein’s active site. Additionally, compound **31** displayed hydrogen bonding between its nitro oxygen atoms and the amino acids LYS94 and HIS96. These interacting amino acids (LEU54, LYS94, and HIS96) play a crucial role in hydrogen bonding with the co-crystallized ligand of the protein used in this study [55].

**Scheme 5:**
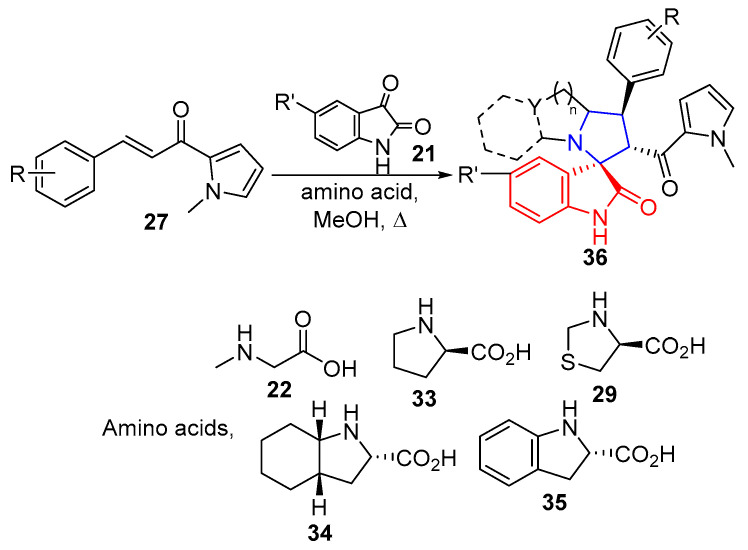
Synthesis of spirooxindoles **36**.

Similar findings have been observed for spirooxindoles **36**, produced through analogous chemical processes. These processes consist of the cycloaddition of azomethine ylides, derived from isatins **21** and various amino acids, including sarcosine **22**, L-proline **33**, L-thioproline **29**, and (2S,3aS,7aS)-octahydro-1H-indole-2-carboxylic acid **34**, with 2-propen-1-ones **27** in refluxing methanol. However, when (S)-indoline-2-carboxylic acid **35** was used alongside 2-propen-1-ones **27** under the same circumstances, the desired cycloadduct was not successfully obtained (Scheme 5). Some synthesized compounds displayed strong antiproliferative effects against MDA-MB 231, HepG-2, and Caco-2 cell lines, as determined by the MTT assay. Additionally, these compounds showed significant MDM2 inhibition, evaluated through a microscale thermophoresis (MST) assay (Supplementary Figure S4) [56].

**Scheme 6:**
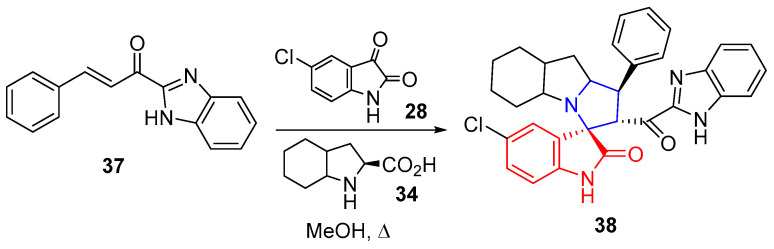
Synthesis of spirooxindole linked to benzimidazolyl heterocycle through carbonyl group **38**.

Spirooxindole linked to the benzimidazolyl heterocycle through carbonyl group **38** was obtained in good yield (75%) via the cycloaddition of benzimidazolyl chalcone **37** with the azomethine ylide formed from 5-chloroisatin **28** and octahydro-1*H*-indole-2-carboxylic acid **34** in refluxing methanol (Scheme 6). Single-crystal X-ray studies aided in assigning the stereochemical configuration. The synthesized analog exhibited potent antiproliferative properties (MTT method) compared to doxorubicin against MDA-MB-231 (breast), PC3 (prostate), HCT-116 (colon), and A549 (lung) carcinoma cell lines (IC_50_ = 2.4 ± 0.2/5.82 ± 0.4, 3.4 ± 0.3/8.80 ± 0.3, 7.2 ± 0.3/13.1 ± 2.1, 7.8 ± 0.3/11.5 ± 0.8 μM for **38** and doxorubicin, respectively). The apoptotic effect of the synthesized compound against the tested cell lines was demonstrated through flow cytometric analysis. Moderate binding was observed against MDM2 (KD = 7.94 μM), with enhancement of p53 expression experimentally supported for the synthesized compound **38**. Docking studies into the p53 binding site of MDM2 (PDB: 1YCR) corroborated the biological observations [57].

**Figure 6:**
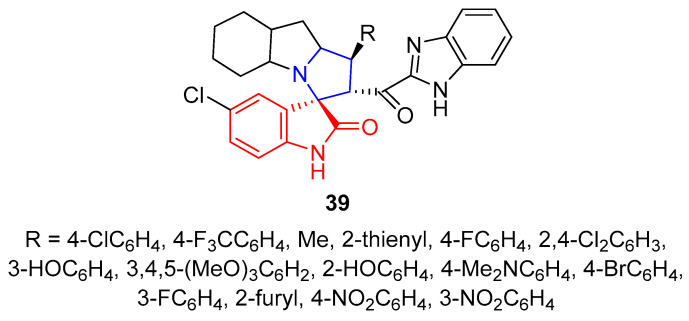
Spirooxindole linked to benzimidazolyl heterocycle **39**.

Another set of spirooxindoles linked to the benzimidazolyl heterocycle, featuring various aryl rings at C-1′ of the pyrrolo[1,2-a]indolyl heterocycle **39**, was prepared using the same synthetic protocol, revealing significant antiproliferation properties (MTT method) (Figure 6). The most promising compound is the one with R = 2-thienyl (IC_50_ = 3.797 ± 0.205 μM against MDA-MB-231 and notable activity against PC3, IC_50_ = 4.314 ± 0.036 μM). Meanwhile, the synthesized analog with R = 4-ClC_6_H_4_ demonstrated the strongest MST binding affinity for MDM2 (KD = 2.38 μM, IC_50_ = 4.763 ± 0.069, and 4.574 ± 0.011 μM against MDA-MB-231 and PC3, respectively). Molecular docking (PDB: 5LAZ, MOE 2019.01 software) clarified and validated the enzymatic results, especially for the most promising agent identified. Supplementary Figure S5 summarizes the antiproliferation and MST inhibitory properties of MDM2 for the synthesized agents [58].

**Scheme 7:**
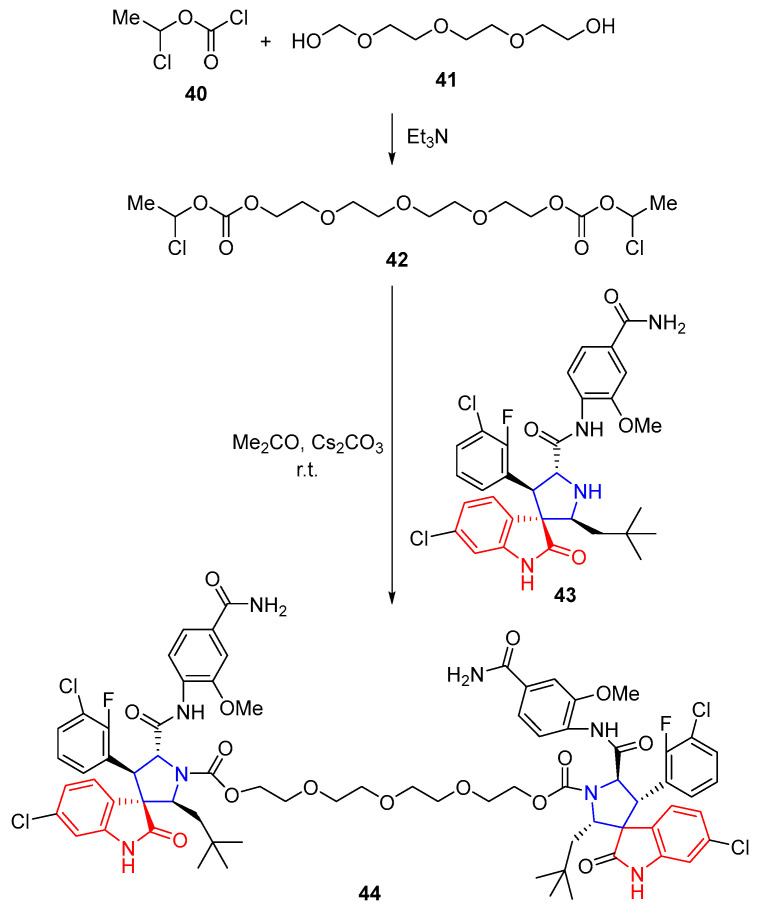
Synthesis of spiroindolinone–pyrrolidinecarboxamide **44**.

A dimer of spiroindolinone–pyrrolidinecarboxamide **44** was designed as a potent agent targeting p53–MDM2, featuring two bio-efficient spiro-scaffolds connected by an ether chain. This hypothesis aims to develop a novel agent that reactivates p53 by inhibiting the MDM2-p53 interaction while minimizing off-target toxicities. The targeted agent was synthesized through the reaction of bis(1-chloroethyl)({[oxybis(ethane-2,1-diyl)]bis(oxy)}bis(ethane-2,1-diyl))bis(carbonate) **42** (an ether chain with two terminal ester groups obtained from reaction of **40** and **41** using Et_3_N as a basic catalyst) and spirooxindole **43** in acetone at room temperature in the presence of Cs_2_CO_3_ (anhydrous) (Scheme 7). The synthesized agent **44** demonstrated an increase in p53 levels in 22Rv1 and LNCaP (prostate cancer cell lines sensitive to MDM2 inhibitors) in a dose-dependent manner. Additionally, upregulation of p53 was observed upon treatment of the HepG2 (liver, wild p53) cancer cell line with **44**. Downregulation of MDM2 (induction of protein degradation) was also reported for **44**, providing evidence of its proteolysis targeting chimeras (PROTAC) activity [59].

**Scheme 8:**
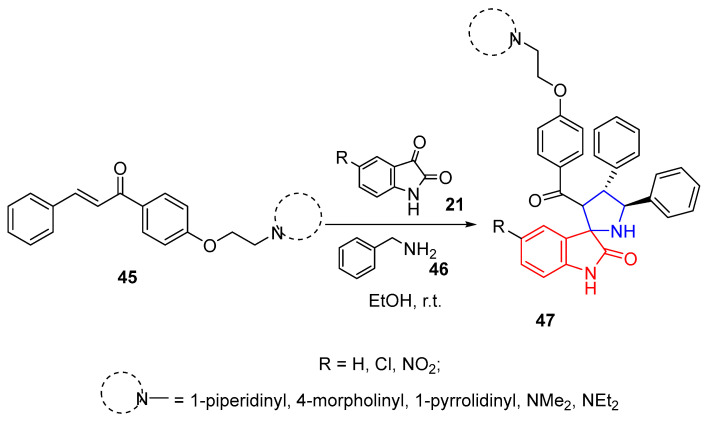
Synthesis of spirooxindoles **47**.

The multi-component cycloaddition of chalcones **45** with azomethine ylides derived from benzylamine **46** and isatins **21** in ethanol at room temperature produced the corresponding spirooxindoles **47** (Scheme 8). Some of the synthesized spirooxindoles exhibited considerable antiproliferative properties (MTT method). The most effective compound had R = Cl and a 1-piperidinyl terminal amine linkage, showing activity against breast cancer cell lines (MD-MB-231 and MCF7) relative to nutlin-3 (IC_50_ = 3.7/23.5 and 6.5/11.6 μM, respectively) while demonstrating safe behavior against human (HEK-293) and monkey (VERO) kidney normal cell lines (IC_50_ = >50 μM). Modulation of MDM2 and p53 expressions was observed for the potent analog synthesized, which induced cell cycle arrest at the G1/S phase. Moreover, it bound to MDM2 and increased the protein levels of both MDM2 and p53 in p53 wild-type cells. Notably, it exhibited minimal toxicity to normal cells, as its cell growth inhibitory IC50 value in HEK-293 cells was approximately 15-fold lower than that in MCF7 cells. Following in vivo testing (xenograft mice model, 20 mg/kg body weight over 14 days), there was a reduction in tumor growth compared to the untreated control group, indicating strong in vivo antitumor efficacy. Furthermore, it displayed limited signs of toxicity, with no observable changes in the liver, spleen, kidney, lung, or uterus based on histomorphological assessments. These data suggest that the promising agent discovered is a potent MDM2/p53 inhibitor with significant anticancer activities both in vitro and in vivo (Supplementary Figure S6) [60].

**Scheme 9:**
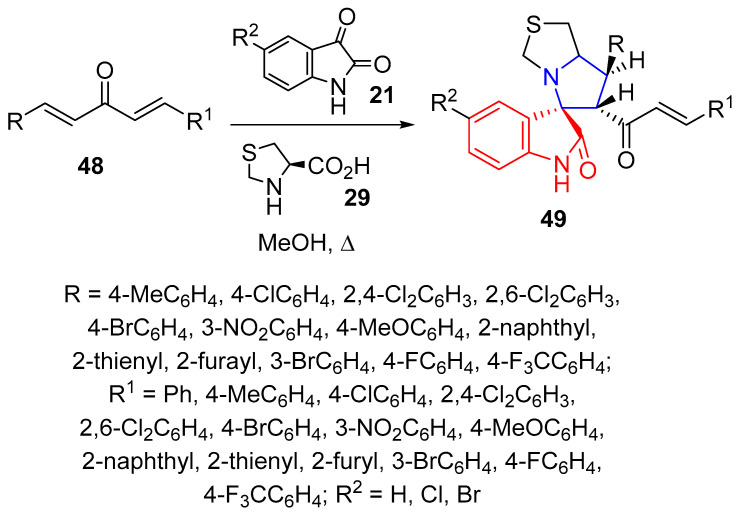
Synthesis of spirooxindoles **49**.

The cycloaddition reaction of enones **48** with azomethine ylides derived from isatin analogs **21** and L-thioproline **29** selectively produced spirooxindoles **49**, whose structures were confirmed by X-ray studies (Scheme 9). The antiproliferative properties were examined using the in vitro MTT assay against HCT116, HepG2, and PC-3 (colon, liver, and prostate) cancer cell lines and VERO-B (normal) cells. Remarkable properties were reported for the synthesized agent with R = R^1^ = 2,4-Cl_2_C_6_H_3_ and R^2^ = H, comparable to cisplatin (IC_50_ = 2 ± 0.6/12.6 ± 2, 0.85 ± 0.2/5.5 ± 1, 1.8 ± 0.3/5.0 ± 0.5, and 5 ± 0.245/5 ± 0.2 μM, respectively). Inhibition of colony formation was also noted, supported by in vitro testing of colon cancer cells and its capability to prevent cell migration and wound healing. Flow cytometric studies demonstrated the arrest of the cell cycle at the G2/M phase. These findings were suggested as potential evidence for the capacity to activate and restore the function of p53, in addition to the docking results (PDB: 5law, Open Eye software version 2.2.5), indicating the inhibition of p53 binding with MDM2 (Supplementary Figure S7) [61].

**Scheme 10:**
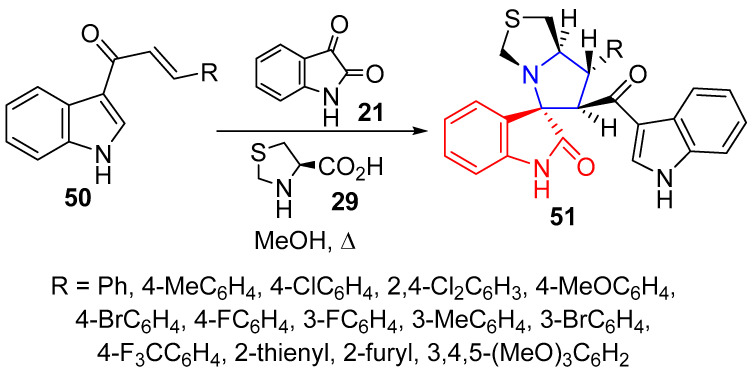
Synthesis of spirooxindoles linked to 3-acylindole **51**.

A group of spirooxindoles linked to 3-acylindole **51** was prepared through reaction of 3-aryl-1-(1-1*H*-indol-3-yl)-2-prop-1-one **50** with azomethine ylide (generated from the condensation of isatin **21**, and *L*-thioproline **29**) in refluxing MeOH (Scheme 10). The antiproliferation properties of the targeted agents were studied (MTT method). Compound with R = 4-F_3_CC_6_H_4_ reveals enhanced properties against HCT116 (colon), and close activity against HepG2 (liver), and PC3 (pancreatic) cancer cell lines relative to cisplatin (IC_50_ = 7 ± 0.27/12.6 ± 0.5, 5.5 ± 0.2/5.5 ± 0.3, 6 ± 0.23/5 ± 0.56 μM, respectively) (Supplementary Figure S8). Safe behavior against VERO-B (normal) cell lines encouraged the consideration of the discovered agent as a promising one for more detailed pharmacological studies needed for pre-clinical and/or clinical investigations. Molecular docking (PDB: 5LAW, Open Eye software version 2.2.5) was adopted for clarifying the MDM2 inhibitory properties where the most promising agent synthesized (R = 4-F_3_CC_6_H_4_) revealed hydrogen bonding interaction of the indolyl NH with LEU54 in similar behavior to that of the co-crystallized ligand in the protein active site [62].

**Figure 7:**
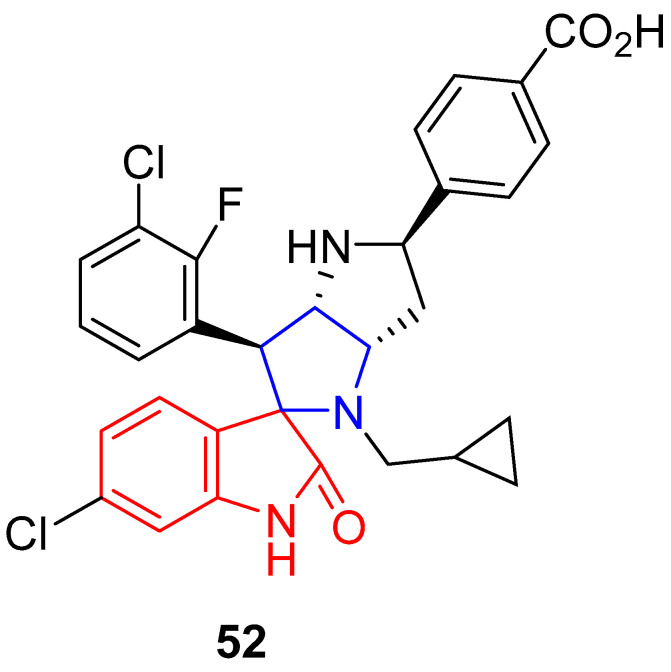
Chemical structure of BI-0252 **52**.

**Scheme 11:**
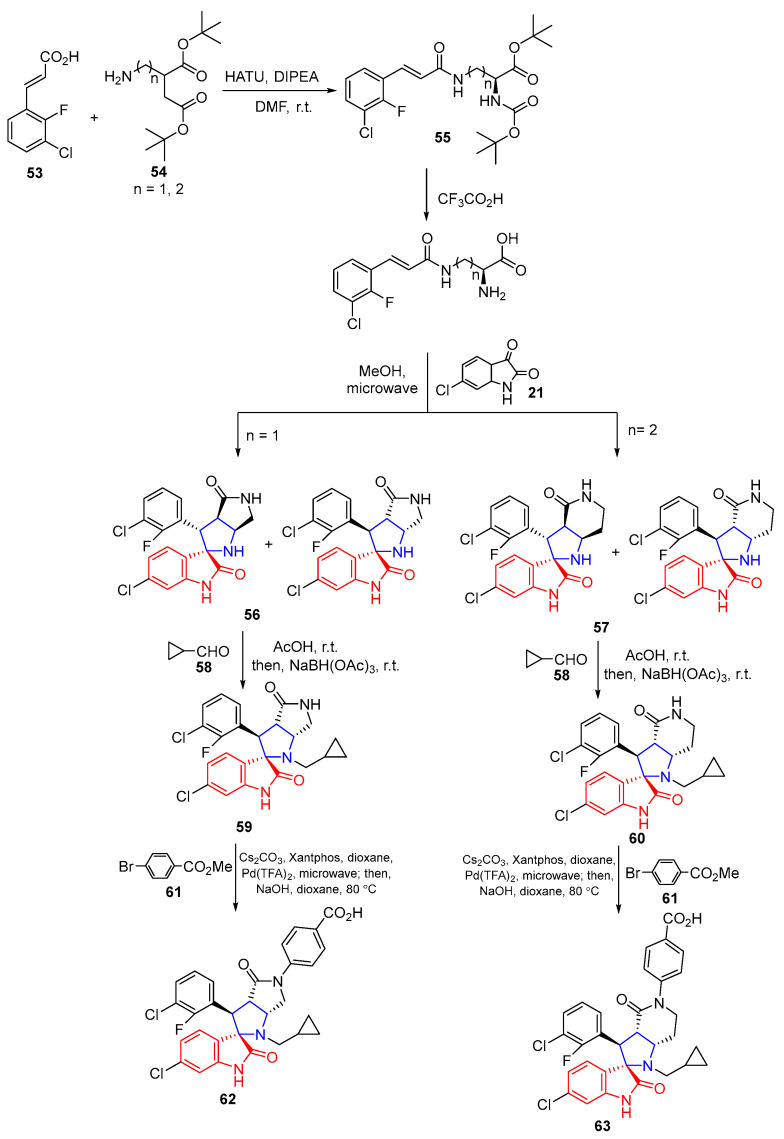
Synthesis of spirooxindoles **62**/**63**.

Efforts to identify an effective agent with properties similar to BI-0252 **52** (Figure 7), which induces tumor regression and exhibits high potency toward p53–MDM2, through intramolecular cycloaddition were considered. The reaction sequence proceeded through the coupling of 3-arylprop-2-enoic acid **53** with boc-amino esters **54** in DMF, containing the peptide coupling agent HATU (hexafluorophosphate azabenzotriazole tetramethyl uranium) and DIPEA (*N*,*N*-diisopropylethylamine, Hünig’s base) at room temperature, resulting in the corresponding amides **55**. This was followed by the removal of the protecting group using trifluoroacetic acid. A cycloaddition reaction conducted with 6-chloroisatin **21** in MeOH under microwave irradiation (100 °C) yielded **56**/**57** as diastereoisomers, which were purified by HPLC (Gilson GX-281 system, SunFire Prep C18, OBD, 10 μm, 50 × 150 mm, using A: water + 0.1% HCOOH, B: acetonitrile HPLC grade as eluent for **56**, and Thar SFC–NEW-200-1, Chiralpak AS-H (250 × 30) mm, using A: CO_2_ 50%, B: Methanol 50% as eluent for **57**). Reductive amination with cyclopropylcarbaldehyde **58** produced **59**/**60**, which underwent Buchwald coupling with methyl 4-bromobenzoate **61** to yield the final targeted agents **62**/**63** (Scheme 11). Biochemical and enzymatic testing of p53–MDM2 (IC_50_ = 4 nM for compounds **52**, **62**, and **63**) and antiproliferation properties against SJSA-1 (an osteosarcoma cancer cell line with wild p53, IC_50_ = 471, 161, 547 nM for compounds **52**, **62**, and **63**, respectively), along with minimal activity against SK-OV-3 (an ovarian cancer cell line with mutant p53, IC50 = >25,000 nM for compounds **52**, **62**, and **63**), supported the selective inhibition of wild p53, with compound **62** (containing a fused five-membered ring system) demonstrating enhanced efficacy over compound **63** (possessing a fused six-membered ring system). Computational studies (PDB: 5LAZ) substantiated the bio-observations [63].

#### 3.1.2. Dispirooxindole–Pyrrolidine

Cycloaddition to exocyclic unsaturated linkages can lead to dispirooxindoles. Numerous dispirooxindole–pyrrolidines combined with alicyclic or heterocyclic scaffolds have been successfully synthesized [64,65,66].

**Scheme 12:**
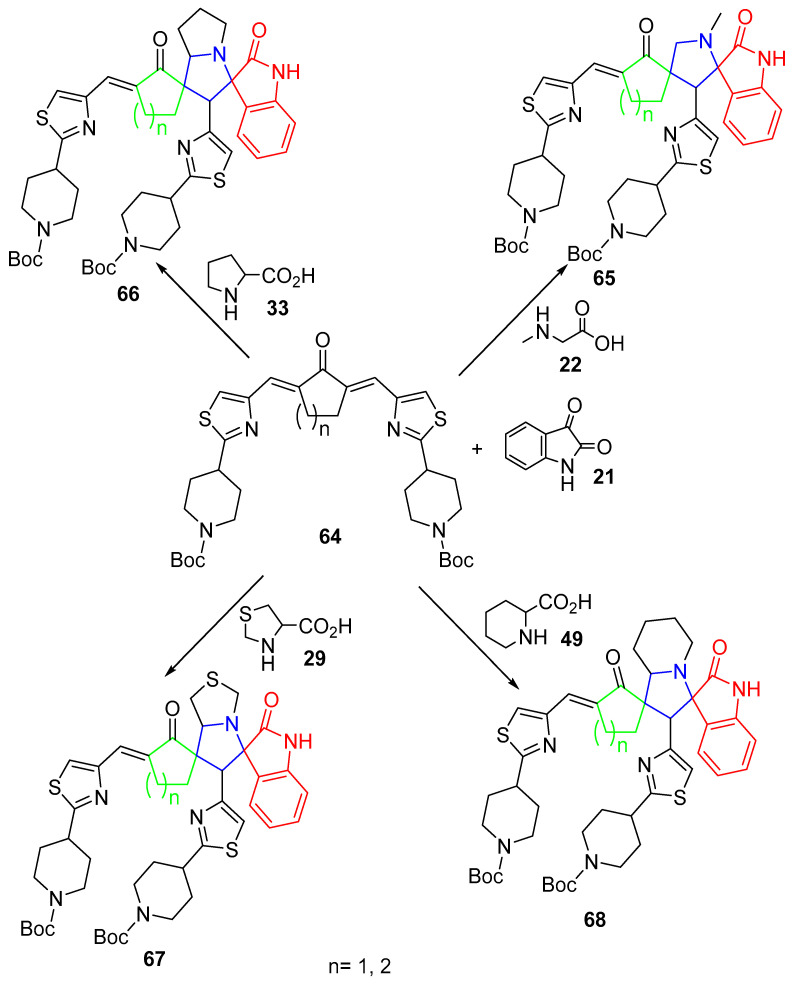
Synthesis of dispirooxindole–pyrrolidines **65**–**68**.

A variety of dispirooxindole–pyrrolidines **65**–**68** was prepared through uncatalyzed cycloaddition of bis(ylidene)cycloalkanones **64** (cyclopentanone/cyclohexanone) with isatin **21** and the respective amino acids (sarcosine **22**, proline **33**, thioproline **29**, or piperidine-2-carboxylic acid **49**) (Scheme 12). Significant antiproliferative properties (MTT method) were reported for the synthesized agents against MCF7 (breast) and HeLa (cervical) cancer cell lines. The most promising analog arose from the reaction of cyclopentanone and piperidine-2-carboxylic acid, demonstrating biological properties similar to those of doxorubicin (IC_50_ = 63.4/59.4, 76.3/65.3 μM against MCF7 and HeLa, respectively) (Supplementary Figure S9). Molecular docking (PDB: 5LAW, AutoDock Vina) was employed to establish the potential mode of action, suggesting the inhibition of the p53–MDM2 interaction [67].

**Scheme 13:**
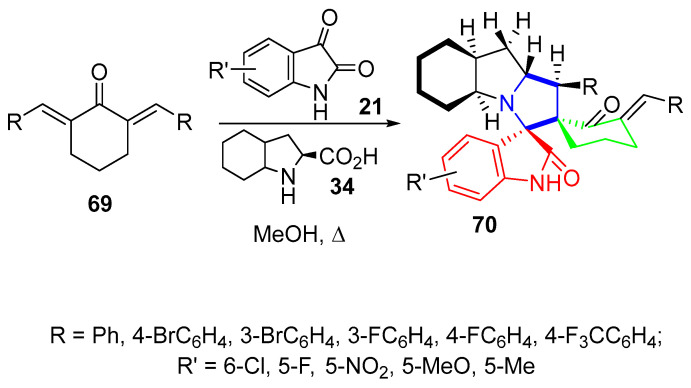
Synthes of dispirooxindole–pyrrolidines **70**.

Another set of dispirooxindole–pyrrolidines **70** was obtained through azomethine cycloaddition of isatins **21** and octahydro-1*H*-indole-2-carboxylic acid **34** with 2,6-bis(ylidene)cyclohexanones **69** in refluxing MeOH (Scheme 13). Antiproliferation properties, as determined by the MTT assay, were reported for the synthesized agents against PC3 (prostate), HeLa (cervical), MCF7, and MDA-MB-231 (breast) cancer cell lines, showing no or minimal cytotoxicity against the BJ (fibroblast) normal cell line. The synthesized analog with R = Ph and R’ = 6-Cl exhibited promising biological properties against PC3, closely resembling those of doxorubicin (IC_50_ = 3.7 ± 1.0/1.9 ± 0.4 μM, respectively) (Supplementary Figure S10). Molecular modeling, including docking studies using PDB: 1T4E and MOE v.2019, has been employed to elucidate and propose the mechanistic anticancer action of a potential MDM2 inhibitor [68].

**Scheme 14:**
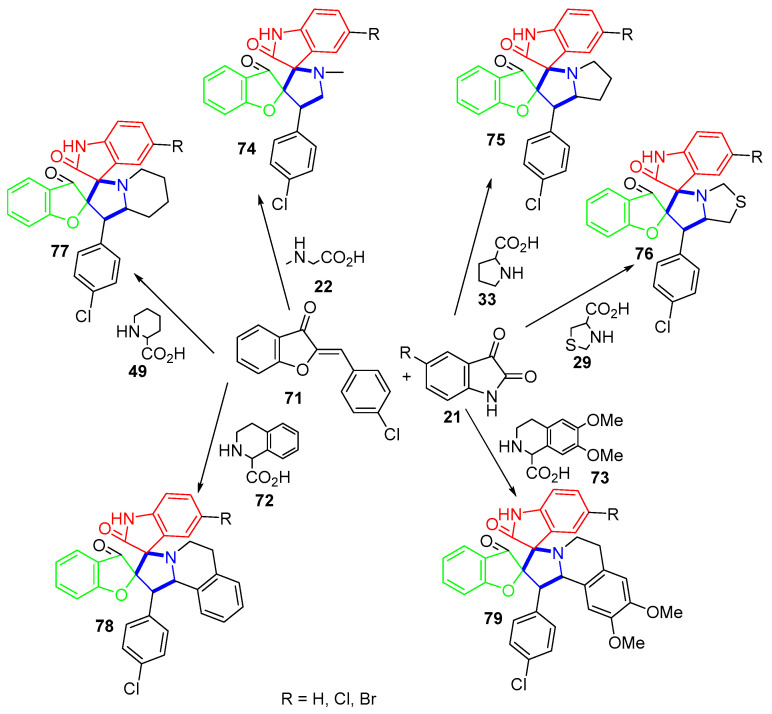
Synthesis of dispirooxindole–pyrrolidines collaborated benzofuranyl heterocycle **74**–**79**.

A variety of dispirooxindole–pyrrolidine-benzofurans **74–79** was synthesized in refluxing ionic liquid [DBU][Ac] (25 mol%) through cycloaddition reaction of 2-arylidene-3(2*H*)-benzofuranone **71** and azomethine ylides obtained from isatins **21** with different α-amino acids (sarcosine **22**, *L*-proline **33**, *L*-thioproline **29**, *L*-picolinic acid **49**, and tetrahydroisoquinolines **72**/**73**) (Scheme 14). X-ray studies were conducted to determine the stereochemical structure of the synthesized analogs. Antiproliferation properties (SRB method) were investigated against various cancer cell lines [A549, SW1573 (lung), HBL-100, T-47D (breast), HeLa (cervical), and WiDr (colon)] and compared to standard references (cisplatin, etoposide, and camptothecin). Compound **77** with R = H demonstrated antitumor properties against the HeLa cell line comparable to those of cisplatin (IC_50_ = 2.5 ± 0.3/2.0 ± 0.3 μM, respectively) (Supplementary Figure S11). Molecular modeling/docking (PDB ID 3LBL, Maestro 9.0) was employed to elucidate the inhibition of MDM2 as the presumed mode of action [69].

**Scheme 15:**
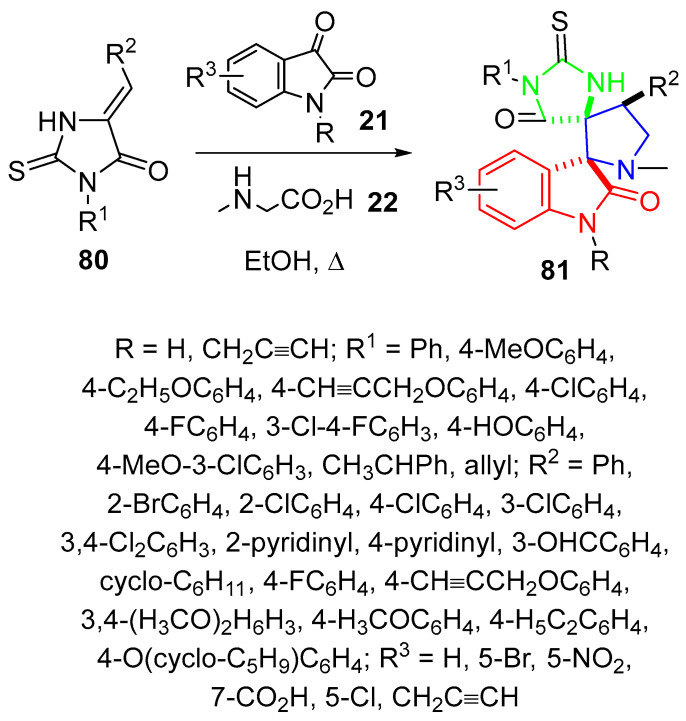
Synthesis of dispirooxindole–pyrrolidines linked to thiohydantoin **81**.

A library of dispirooxindole–pyrrolidine–thiohydantoins **81** (66 analogs) was obtained through cycloaddition of azomethine ylides (isatins **21**, and sarcosine **22**) with 5-ylidene-2-thiohydantoins **80** in refluxing EtOH. X-ray studies showed that the resulting agents are diastereomers (Scheme 15). Few synthesized agents demonstrated promising antiproliferation properties (MTT method) against LNCaP (p53+, pancreatic cancer cell line, IC_50_ = 1.2–3.5 μM) compared to nutlin-3a (IC_50_ = 2.7 μM). While molecular docking (PDB: 4JVR, MOE software) was employed to estimate the possible mode of action of the targeted agents as MDM2 inhibitors, the Western blotting technique did not verify the assumption, suggesting that the cytotoxic effect may arise from an alternative biochemical mechanism (Supplementary Figure S12) [13].

**Scheme 16:**
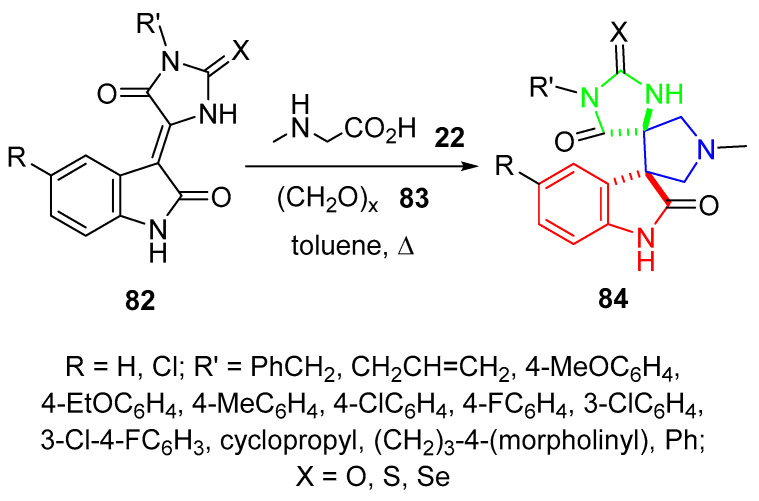
Synthesis of dispirooxindole–pyrrolidines **84**.

Dispirooxindole–pyrrolidine–hydantoins/thiohydantoins/or selenohydantoins **84** were obtained through a reaction of the corresponding 3-ylidene-2-indolinones **82** with azomethine ylide formed in situ from sarcosine **22** and paraformaldehyde **83** in refluxing toluene (Scheme 16). The absolute configuration was assigned for the synthesized agents based on X-ray crystallographic studies. Cytotoxicity (MTT assay) was evaluated against a panel of cancer cell lines [A549 (lung); MCF7 (breast); VA13 (SV40-transformed lung); Hek293T (embryonic kidney); LNCaP, PC3 (prostate); in addition to HCT116^+/+^, HCT116^−/−^ (colon positive, and negative p53)] (Supplementary Figure S13). Although one of the synthesized analogs with X = S, R = Cl, R’ = 4-MeOC_6_H_4_, exhibited comparable efficacy to that of nutlin-3 against both HCT116^+/+^, and HCT116^−/−^ (CC_50_ = 1.95 ± 0.43/3.3 ± 0.13, 2.35 ± 0.95/35.12 ± 2.65 μM, respectively), lack of selectivity, humbled the observations. Molecular docking (PDB: 4JVR, ICM Pro software, https://www.molsoft.com) was considered to elucidate the possible binding of the investigated agents towards MDM2. Hydrogen bonding was observed between the indolinyl NH and LEU54 of the protein active site. High concentration (>100 μM) of the synthesized agents revealed activation of p53. However, at that mentioned dose, most of the compounds exhibited toxicity towards the tested cells, with only about 7–10% of the tested cells remaining alive. This observation concluded that the promising agents discovered may have a mode of action other than that of the p53–MDM2 effect [70].

**Scheme 17:**
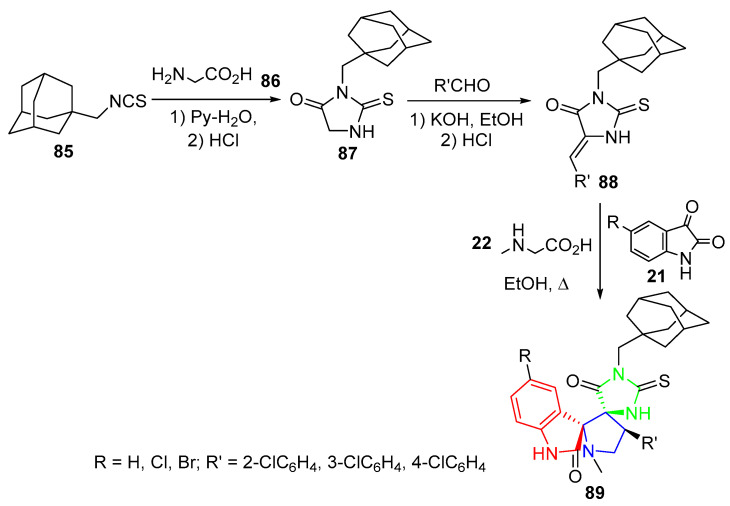
Synthesis of dispirooxindole–pyrrolidines **89**.

Dispirooxindole–pyrrolidine–thiohydantoins attached to adamantine **89** were diastereoselectively synthesized through the cycloaddition of azomethine ylide (isatins **21** and sarcosine **22**) with 5-ylidene-2-thioxoimidazolidin-4-ones **88** (obtained from a multistep reaction sequence of isothocyanate analog **85** with glycine **86** affording imidazolidinone **87** followed by condensation with various aromatic aldehydes giving **88**) in refluxing ethanol (Scheme 17). The synthesized analog with R = Br and R’ = 2-ClC_6_H_4_ showed better antiproliferative efficacy (MTT test) among all the synthesized agents. It was comparable to the standard references (nutlin-3a and cisplatin) [IC_50_ = 7.2 ± 1.85/14.9 ± 0.6/44.13 ± 3.9, 7.63 ± 0.75/10.4 ± 0.8/12.4 ± 3.9 μM against A549 (lung) and HEK293T (embryonic kidney) cancer cell lines] (Supplementary Figure S14). Despite the considerable antiproliferative properties revealed by some of the constructed agents, no significant p53 receptor activation was demonstrated in the A549 cell line testing. Only the synthesized analog with R = Br and R’ = 4-ClC_6_H_4_ exhibited mild p53 receptor activation (2.03) at 100 μM, relative to nutlin-3a (which revealed p53 receptor activation 5.1-fold at 100 μM). These observations indicate that a mechanism distinct from the p53–MDM2 interaction may regulate the observed antiproliferative behaviors [71].

**Scheme 18:**
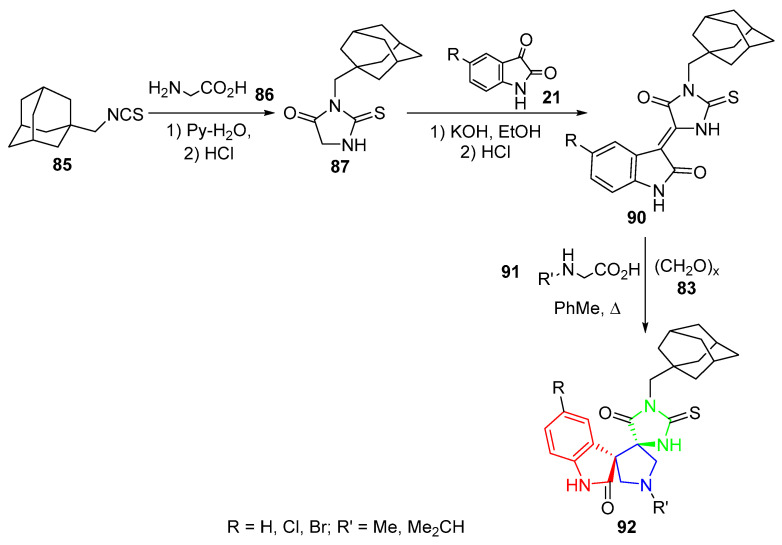
Synthesis of dispirooxindole–pyrrolidines **92**.

Similarly, dispirooxindole–pyrrolidine–thiohydantoins **92** were prepared through an azomethine reaction (obtained from paraformaldehyde **83** and α-amino acids **91**) with 4-(imidazolidinylidene)-2-indolinones **90** in refluxing toluene (Scheme 18). The synthesized analog with R = Cl and R’ = Me showed considerable antiproliferative properties relative to nutlin-3a and cisplatin (MTT method, IC_50_ = 5.0 ± 0.6/14.9 ± 0.6/44.13 ± 3.9, 2.8 ± 0.3/10.4 ± 0.8/12.4 ± 3.9 μM against A549 and HEK293T, respectively) (Supplementary Figure S15). The synthesized agent **92** with R = H and R’ = Me exhibited p53 reporter activation ranging from 1.06 to 4.39, comparable to nutlin-3a, which revealed activation ranging from 3.6 to 5.1 folds during A549 cell line testing for 1.56 to 100 μM compound concentrations. This observation suggests that the cytotoxicity observed in these analogs may be enhanced by the p53–MDM2 effect in addition to other modes of action that collectively contributed to the bio-observations [71].

**Scheme 19:**
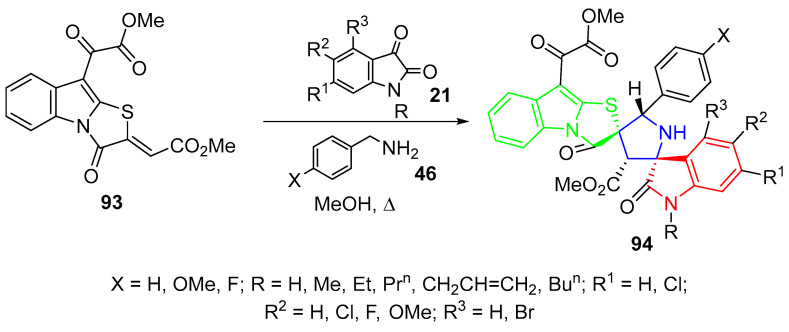
Synthesis of dispirooxindole–pyrrolidine **94**.

Dispirooxindole–pyrrolidine–thiazolo[3,2-*a*]indoles **94** were synthesized through the cycloaddition of (thiazolo[3,2-*a*]indol-9-yl)-2-oxoacetate **93** with azomethine ylides generated in situ from benzylamines **46** and isatins **21** in refluxing MeOH (Scheme 19). Gaussian 09 quantum chemical calculations at the B3LYP/6-311G^++^(2df,2p) level discussed the stereochemical selectivity of the reaction, and X-ray studies confirmed the structure. Antiproliferation properties (MTT technique) against the MCF7 (breast) cancer cell line showed mild activity of the analog synthesized with X = F, R = Pr*^n^*, R^1^ = Cl, R^2^ = R^3^ = H (about 80% cell viability at 25 μg/mL). Meanwhile, the synthesized agent with X = H, R = Pr*^n^*, R^1^ = Cl, R^2^ = R^3^ = H exhibited 80% cell viability at 200 μg/mL. Molecular docking (AutoDock Vina, PDB: 5LAW) was employed to establish/explain the MDM2 binding as the mode of action of the synthesized agents. The promising agent identified (X = F, R = Pr*^n^*, R^1^ = Cl, R^2^ = R^3^ = H) demonstrated hydrogen bonding interaction with LEU54, like the lead compound in the protein active site [72].

#### 3.1.3. Spirooxindole–Pyrazoline

**Scheme 20:**
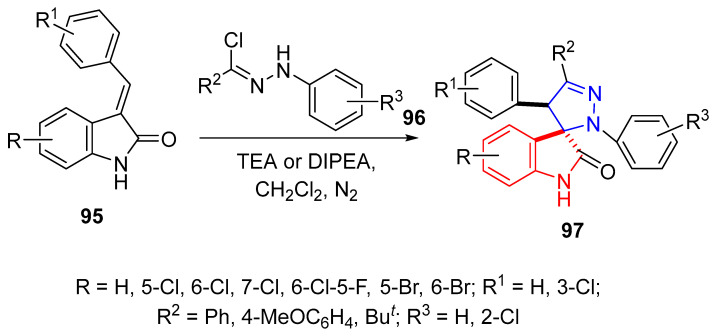
Synthesis of spirooxindole–pyrazolines **97**.

Spirooxindole–pyrazolines **97** were synthesized through the cycloaddition of nitrilimines, generated from the basic dehydrochlorination (with triethylamine “TEA” or *N*,*N*-diisopropylethylamine “DIPEA”) of hydrazonoyl chlorides **96**, with 3-ylidene-2-indolinones **95** in CH_2_Cl_2_ under a nitrogen atmosphere (Scheme 20). Promising antiproliferative properties were observed against HCT116 p53^+/+^ (colon with wild-type p53) cancer, with no cell death noted in CCD-18Co (normal/non-cancer colon fibroblast) cell lines. The most effective agents identified had R = 5-Br, R^1^ = R^3^ = H, R^2^ = Ph; R = 6-Cl, R^1^ = 3-Cl, R^2^ = 4-MeOC_6_H_4_, R^3^ = Ph (IC_50_ = 13.1 ± 1.0, 10.9 ± 0.8 μM, respectively) (Supplementary Figure S16). Induction of apoptosis and cell cycle arrest at G0/G1 was evidenced for the effective agents identified through flow cytometric studies. A Venus-based bimolecular fluorescence complementation (BiFC) assay was employed to elucidate the p53–MDM2 interaction affected by the promising agents discovered. A mild decrease in BiFC was observed at a 20 μM concentration, supporting the protein-protein (p53–MDM2) interaction disruption. The Western blotting assay revealed increased p53 levels and decreased MDM2 levels when testing the promising agents discovered (utilizing the reported IC_50_ values) with the HCT116 p53^+/+^. However, the detection of p53 for the promising compounds against HCT116 p53^−/−^ showed similar findings (IC_50_ = 14.2 ± 0.8, 11.6 ± 0.8 μM, respectively), suggesting that the antiproliferative properties of the compounds tested may be due to the contribution of the p53–MDM2 interaction alongside other anti-malignancy modes of action [73].

**Scheme 21:**
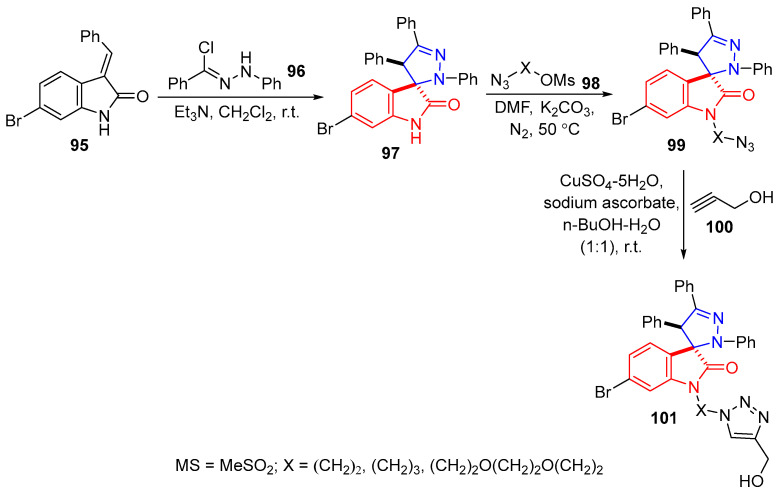
Synthesis of spirooxindole–pyrazolines linked to triazolyl heterocycle **101**.

Spirooxindole–pyrazoline **97** (mentioned in Scheme 20, where R = 6-Br and R^1^ = R^2^ = R^3^ = H) underwent alkylation using various azidoalkanes with mesylate (methane sulfonyl) terminal **98** in DMF containing K_2_CO_3_ at 50 °C under an inert (nitrogen) atmosphere, resulting in spiro-analogs **99**. The latter compound was allowed to react with propargyl alcohol **100** in n-BuOH-H_2_O (1:1 *v*/*v*) containing CuSO_4_-5H_2_O and sodium ascorbate at room temperature, ultimately furnishing the targeted spiro-analogs linked with triazolyl heterocycle **101** (Scheme 21). Compound **101**, with X = (CH_2_)_2_, demonstrated notable efficacy against breast cancer (MCF7 “wild p53” and MDA-MB-231 “mutant p53”) cell lines (MTT assay, IC_50_ = 11.9 and 9.30 μM, respectively) [74] (Supplementary Figure S17).

**Scheme 22:**
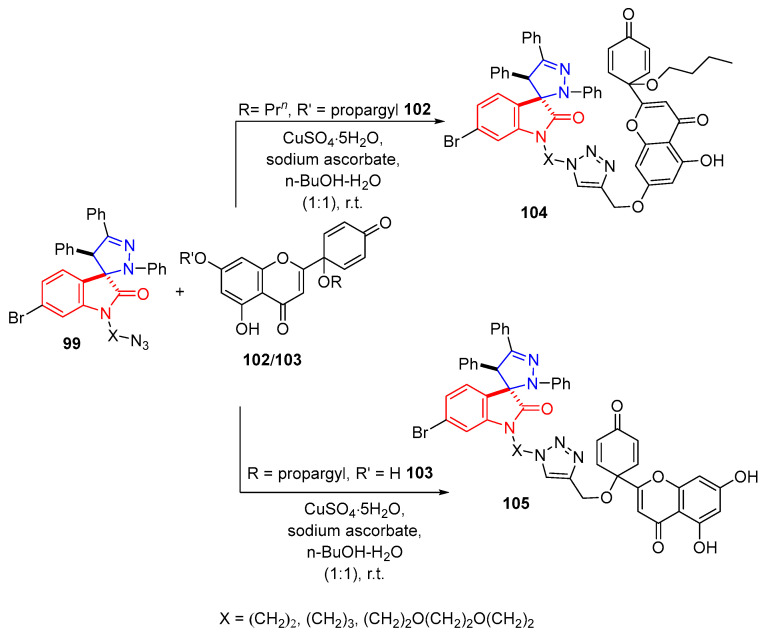
Synthesis of spirooxindole–pyrazolines conjugated with flavone **104** and **105**.

Attempts were considered for conjugation of spirooxindolyl heterocycle (due to p53–MDM2 effect) with flavone [due to ataxia telangiectasia and Rad3-related protein (ATR) inhibitory effect] considering the combination of bioactive rings approach, for developing potent anti-malignant active agents [75,76,77]. Spirooxindole–pyrazolines **99** were allowed to react with flavones **102**/**103** in n-BuOH-H_2_O (1:1 *v*/*v*) containing CuSO_4_·5H_2_O and sodium ascorbate at room temperature, giving the corresponding spiro-analogs linked with flavone heterocycle **104**/**105** (Scheme 22). Notable antiproliferation properties against breast cancer cell lines (MTT method, IC_50_ = 2.92, 1.08, 4.56 μM against MCF7 “wild p53”, MDA-MB-231 “mutant p53”, and MCF 10A, respectively) were reported for the synthesized conjugate **105** with X = (CH_2_)_2_ (Supplementary Figure S18). Additionally, it has also been reported that the synthesized analog with X = (CH_2_)_2_O(CH_2_)_2_O(CH_2_)_2_ can inhibit the ATR-dependent activation of Chk-1 beside activation of Chk-2 (serine–threonine checkpoint kinases) [74].

#### 3.1.4. Spirooxindole–Isoxazoline

**Scheme 23:**
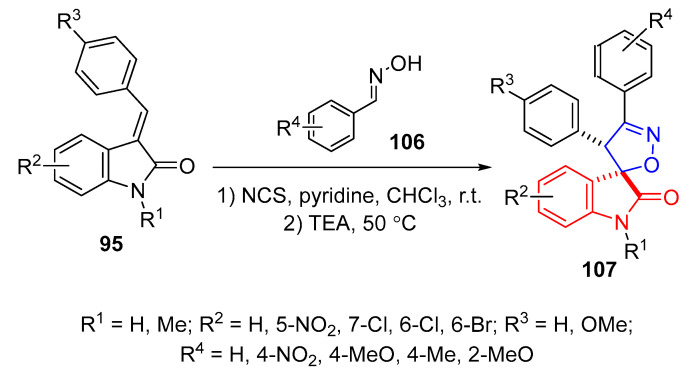
Synthesis of spirooxindole–isoxazolines **107**.

A set of spirooxindole–isoxazolines **107** was synthesized through the cycloaddition of 3-ylidene-2-indolinones **95** with nitrile-*N*-oxides, which were generated in situ from the corresponding hydroximoyl chlorides. This was achieved by stirring aldoximes **106** with *N*-chlorosuccinimide (NCS) in CHCl_3_ containing pyridine at room temperature for 24 h, followed by dehydrochlorination using TEA (Scheme 23). Antiproliferative properties were assessed using an aqueous non-radioactive cell proliferation method (Promega) against HepG2 (hepatocellular wild p53 type), as well as against HCT116 (p53^+/+^), HCT116 (p53^−/−^), and SW620 (mutant p53) cancer cell lines (Supplementary Figure S19). The synthesized agent with R^1^ = R^3^ = H, R^2^ = 6-Cl, and R^4^ = 4-Me showed promising properties compared to nutlin-3 (GI_50_ = 29.11 ± 1.09/51.31 ± 1.04, 26.56 ± 1.07/39.65 ± 1.12, 30.64 ± 1.12/52.34 ± 1.15, 31.56 ± 1.05/57.04 ± 1.04 μM, respectively). The synthesized analog with R^1^ = R^3^ = R^4^ = H and R^2^ = 6-Cl exhibited inhibitory effects on the p53–MDM2 interaction relative to nutlin-3, demonstrated by the BiFC method. It was concluded that some of the constructed compounds are promising leads, potentially useful for optimizing antitumor-effective agents in drug-discovery programs [78].

#### 3.1.5. Spirooxindole–Triazole

**Scheme 24:**
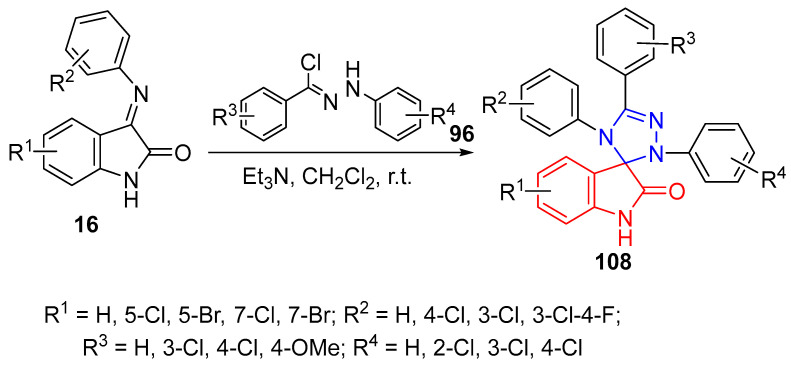
Synthesis of spirooxindole–triazoles **108**.

Spirooxindole–triazoles **108** were synthesized through the cycloaddition of 3-imino-2-indolinones **16** with nitrilimines (produced from triethylamine dehydrochlorination of hydrazonoyl chlorides **96**) in CH_2_Cl_2_ at room temperature (Scheme 24). Several of the synthesized compounds (R^1^ = 5-Cl/5-Br, R^2^ = 3-Cl/3-Cl, R^3^ = H/3-Cl, R^4^ = H/2-Cl) demonstrated promising antiproliferative activity (MTT method) against MCF7, MDA-MB-231 (breast), HCT116 (p53^+/+^), HCT116 (p53^−/−^) (colon) cancer, and HEK 293T (embryonic kidney non-cancer) cell lines (IC_50_ = 9.8 ± 1.5/9.5 ± 3.1, 8.9 ± 1.9/7.6 ± 2.4, 16.3 ± 0.2/13.9 ± 1.1, 17.9 ± 0.6/17.7 ± 1.3, >100/21.4 ± 7.4 μM, respectively) and compared with that of nutlin-3a [IC_50_ = 4.0 ± 1.2, 47.8 ± 1.9 μM against HCT116 (p53^+/+^), and HCT116 (p53^−/−^), respectively] (Supplementary Figure S20). No cell death was observed in the CCD-18Co cell line (a non-cancerous colon cell line), indicating that the agents are safe for normal cells. The induction of apoptosis and cell cycle arrest in the G0/G1 phase, coupled with the upregulation of p53 and inhibition of MDM2 in the HCT116 cell line, provide strong evidence supporting the efficacy of these promising agents. These findings suggest that further bio-studies should be carried out before preclinical and clinical investigations [79].

#### 3.1.6. Spirooxindole–Oxadiazole

**Scheme 25:**
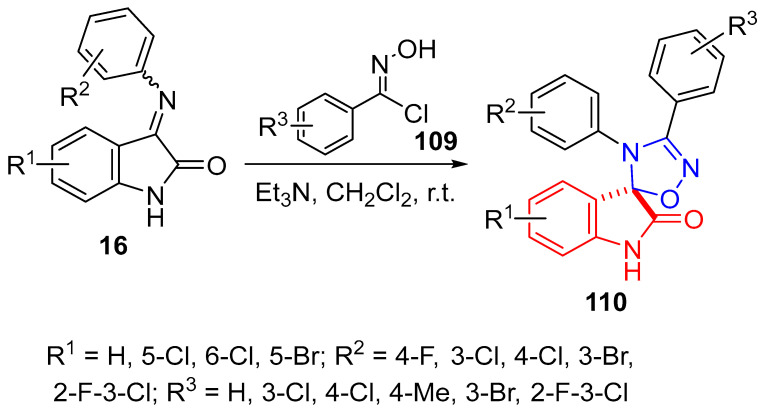
Synthesis of spirooxindole–oxadiazoles **110**.

The cycloaddition of nitrile-*N*-oxide (generated in situ through the dehydrochlorination of hydroxyl chlorides **109**) with 3-imino-2-indolinones **16** in CH_2_Cl_2_ at room temperature led to the formation of the corresponding spirooxindole–oxadiazoles **110** (Scheme 25). The synthesized analog with R^1^ = 5-Br, R^2^ = 3-Cl, R^3^ = 3-Cl demonstrated promising antiproliferation properties [aqueous non-radioactive cell method (Promega)] against HCT116 (p53^+/+^), HCT116 (p53^−/−^) (colon), HepG2 (liver, wild p53 type), and SW620 (colon, mutant p53) cell lines (GI_50_ = 2.0 ± 0.0, 2.9 ± 0.2, 2.1 ± 0.0, 2.1 ± 0.3 μM, respectively) compared to nutlin-3a [GI_50_ = 4.0 ± 1.2, 47.8 ± 1.9 μM against HCT116 (p53^+/+^), and HCT116 (p53^−/−^), respectively] (Supplementary Figure S21). It was experimentally (BiFC assay) that the promising agent inhibited the p53–MDM2 interaction and induced p53 stabilization [80].

### 3.2. Spirooxindole Combined with Six-Membered Heterocycle

#### 3.2.1. Spirooxindole–Piperidine

**Scheme 26:**
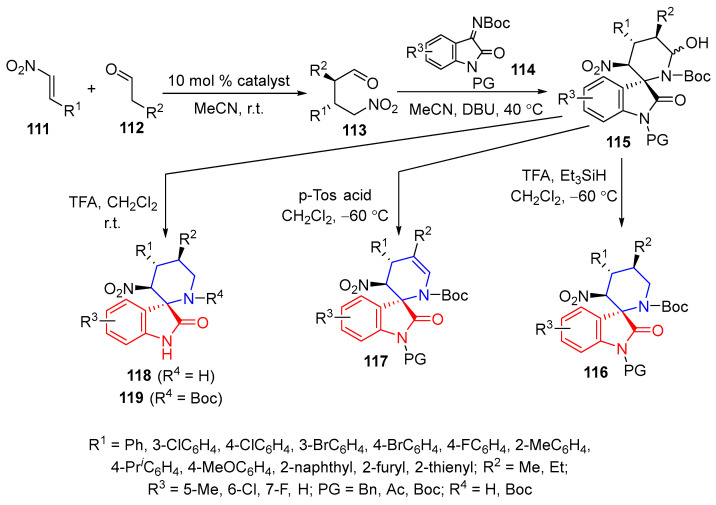
Synthesis of spirooxindole–piperidines **116**–**119**.

A variety of spirooxindole–piperidines **116** were synthesized through a multi-step reaction. The one-pot reaction began with a Michael addition between nitroalkenes **111** and saturated aldehydes **112** in MeCN containing DBU (10 mol % catalyst) at room temperature, giving **113**, that followed by the addition of isatin ketimines **114** at 40 °C, resulting in the spiro-analogs **115**. Dehydroxylation was accomplished using triethyl silane (Et_3_SiH) in CH_2_Cl_2_ with TFA (trifluoroacetic acid) at −60 °C, affording the targeted spirooxindole–piperidines **116** in good diastereoselectivity/enantioselectivity. However, stirring **115** with *p*-toluene sulfonic (*p*-Tos) acid in CH_2_Cl_2_ at −60 °C gave **117**. Compounds **118** (R^4^ = H) were obtained from **115** upon the addition of TFA at room temperature for 24 h. However, conducting the same reaction for 10 min, the corresponding **119** (R^4^ = Boc) were obtained (Scheme 26). Some of the synthesized analogs showed considerable antiproliferation properties (MTT assay) against MCF7, ZR-75-1 (wild-type p53), and BT-474 (mutated-type p53) breast cancer cell lines (Supplementary Figure S22). The most promising is **119** with R^1^ = 4-ClC_6_H_4_, R^2^ = Me, R^3^ = 6-Cl, R^4^ = Boc (% inhibition at 20 μM = 93.03, 82.28 and 50.00, for the tested cell lines, respectively). Encouraging inhibitory activity against MDM2 (HTRF method) was also observed (84.86 at 20 μM). Cell cycle arrest in the G0/G1 phase and mitochondrial apoptosis (flow cytometry and fluorescence microscope) were reported. Molecular docking and dynamic studies (AMBER 12.0) were performed (PDB: 4LWU) to gain insight into the biological observations of the effective agent discovered [81].

#### 3.2.2. Spirooxindole–Pyran

**Scheme 27:**
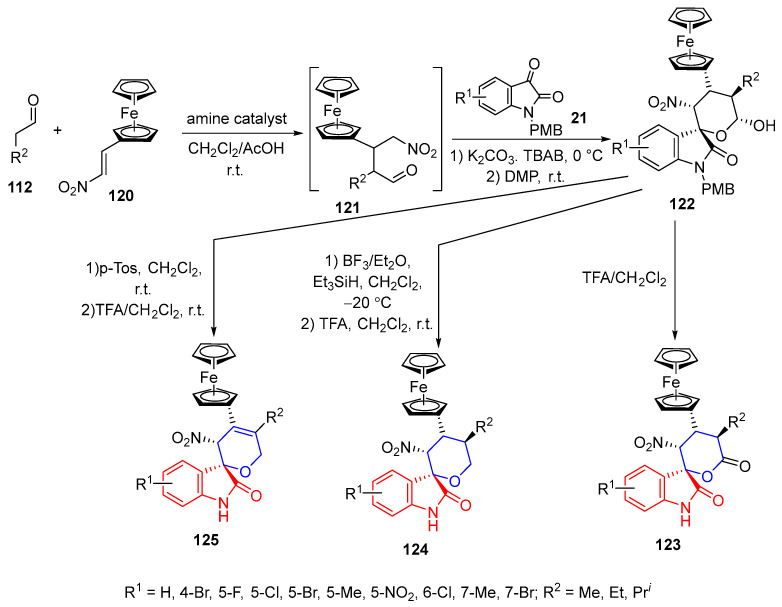
Synthesis of spirooxindole–pyrans **123**–**125**.

A set of spirooxindole–pyrans linked to the ferrocenyl ring **123**–**125** was prepared through a reaction sequence similar to that described for spirooxindole–piperidines (Scheme 26), beginning with the addition of ferrocenyl-substituted nitroalkenes **120** and saturated aldehydes **112** in CH_2_Cl_2_ and AcOH, using Hayashi–Jørgensen secondary amine as a catalyst. The resulting Michael adduct intermediates **121** were allowed to react with the appropriate isatin **21** in a one-pot reaction with K_2_CO_3_ and tetrabutylammonium bromide (TBAB) at 0 °C. Oxidation of these compounds using Dess–Martin Periodinane (DMP) at room temperature led to the formation of the target spirooxindole–pyrans **122**. Treatment of these with TFA/CH_2_Cl_2_ ultimately yielded **123**. However, treating **122** with BF_3_/Et_2_O and Et_3_SiH in CH_2_Cl_2_ at −20 °C, followed by TFA in CH_2_Cl_2_ at room temperature, produced **124**. Meanwhile, treatment of **122** with *p*-Tos in CH_2_Cl_2_ at room temperature, followed by TFA/CH_2_Cl_2_, resulted in the corresponding **125** (Scheme 27). Diastereoselectivity and enantioselectivity were noted and supported by X-ray studies. Some of the synthesized analogs were considered promising antiproliferative agents against breast cancer cell lines, among which compound **123** with R^1^ = 5-Cl, R^2^ = Me (IC_50_ = 0.88 ± 0.08, 0.67 ± 0.06, 4.63 ± 0.38, 6.27 ± 0.49, 3.56 ± 0.47, 6.59 ± 0.81, 5.61 ± 0.36 μM against MCF7, ZR-75-1 “wild-type p53”, BT-474 “mutated-type p53”, HCC1937, MDA-MB-231, MDA-MB-435, and SKBR-3, respectively) showed bio-properties against MDM2 protein (K_i_ = 0.53 ± 0.03 μM) (Supplementary Figure S23). Molecular docking studies (Discovery Studio 3.5 software, PDB: 4LWU) were utilized to explain the observed bio-properties [82].

#### 3.2.3. Spirooxindole–Benzopyran

**Scheme 28:**
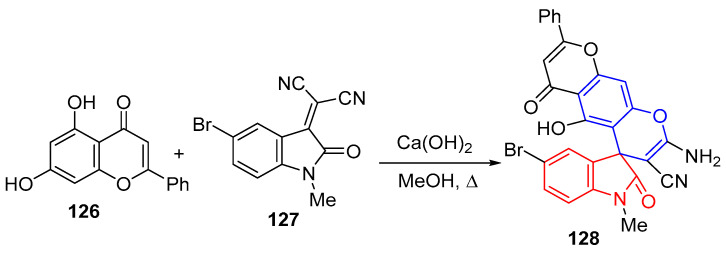
Synthesis of spirooxindole–benzopyran **128**.

Attempts towards the construction of natural compound-hybridized scaffolds (indole-benzopyran) were undertaken to develop potent antitumor active agents with selectivity/safety profiles. Chrysin (5,7-dihydroxy-2-phenyl-4*H*-1-benzopyran-4-one) **126**, a natural compound found in honey, propolis (bee glue), and plants (e.g., *Passiflora* sp.), underwent addition reaction with (2-oxoindolin-3-ylidene)malononitrile derivative **127** in refluxing MeOH containing Ca(OH)_2_**,** giving rise to spirooxindole–benzopyran **128** (Scheme 28). Promising antiproliferation properties were mentioned for **128** against A549 (lung cancer) with potential selectivity towards normal MRC-5 cells and in vivo xenographic observations in C57BL/6 mice with lung cancer. Inhibition of transplanted cancer in mice (100, 200, 400 mg/kg of the tasted agent in saline solution with tween-80 and 0.5% sodium carboxymethyl cellulose) with induction of apoptosis was also evidenced. Gradual Bax/Bcl-2 increment was also mentioned due to the elevated dosage. However, no significant effect was noticed on Cyt c protein when the low and medium dosages were used. The antitumor activity was attributed to the inhibition of MDM2, Akt, and 5-Lox proteins. The distinguished observations concluded that the synthesized **128** can be considered a promising hit valid for developing clinically useful anti-cancer drugs [83].

**Scheme 29:**
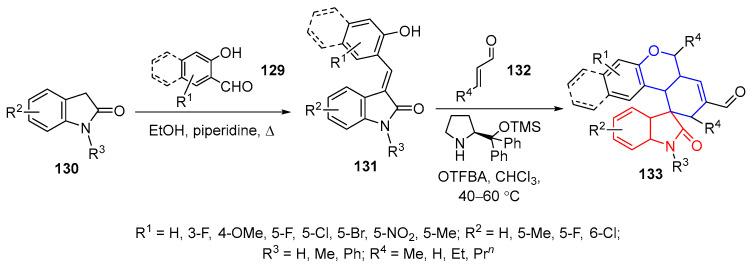
Synthesis of spirooxindole–benzopyran **133**.

3-Ylidene-2-indolinones **131** (obtained through the condensation of salicaldehydes/2-naphthaldehyde **129** and indolin-2-ones **130** in refluxing EtOH with piperidine as a basic catalyst) were allowed to react with crotonaldehyde **132** in CHCl_3_ (40–60 °C) in the presence of α,α-L-diphenylprolinol trimethylsilyl ether and 2-(trifluoromethyl)benzoic acid (OTFBA), resulting in the formation of spirooxindole–benzopyran **133** (Scheme 29). X-ray studies confirmed the structure. The synthesized analog derived from naphthaldehyde with R^2^ = R^3^ = H and R^4^ = Me exhibited promising antiproliferation properties against various cancer cell lines compared to those of cisplatin (MTT method, IC_50_ = 7.9/20.9, 23.4/27.3, 9.5/23.7, 8.6/17.4, 18.3/15.6, 16.2/7.8, 13.7/9.7 μM against A2870s, A2870T “ovarian”, CT26, HCT116 “colon”, A549 “lung”, MCF7 “breast”, and H1975 “lung”, respectively) (Supplementary Figure S24). It also demonstrated a potential effect in activating caspase-3 (supporting its ability to induce apoptosis) and upregulating MDM2 levels, expecting activation of p53, as evidenced by experimental Western blotting tests utilizing HCT116 cells. Molecular docking studies (GOLD software, version 5.0, PDB: 4OAS) were employed to elucidate the blocking behavior of the p53–MDM2 interaction of the discovered effective agent [84].

#### 3.2.4. Spirooxindole–Thiopyran

**Scheme 30:**
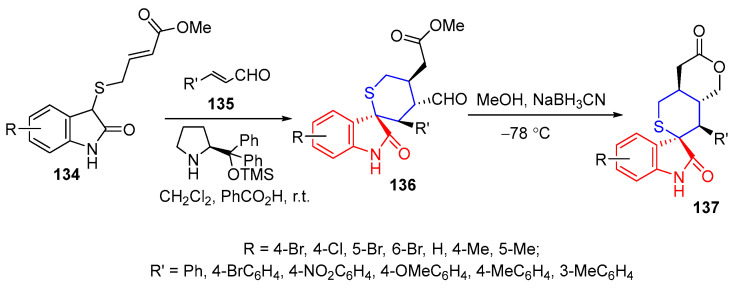
Synthesis of spirooxindole–thiopyrans **136**, and **135**.

The organocatalytic enantioselective reaction (using diphenylprolinyl silyl ether in the presence of PhCO_2_H) of (E)-4-[(2-oxoindolin-3-yl)thio]but-2-enoate methyl ester **134** with the appropriate acrylaldehydes **135** produced the corresponding spirooxindole–thiopyrans **136** with good diastereo- and enantioselectivities. A reductive condensation reaction (using MeOH and sodium cyanoborohydride “NaBH_3_CN” at −78 °C) yielded the spiro analogs **137** (see Scheme 30). The antiproliferation properties against wild-type A549 (lung), HCT116 (colon), and MDA-MB-231 (breast) carcinoma cell lines (MTT method) were examined (Supplementary Figure S25). The spiro analog **136** with R= 4-Br and R’ = 4-BrC_6_H_4_ demonstrated promising bio-properties compared to nutlin-3 (IC_50_ = 1.67/2.22, 1.57/1.16, 3.55/4.68 μM, respectively). Western blotting studies confirmed the mode of action of the promising agent discovered as a p53–MDM2 inhibitor [85].

**Scheme 31:**
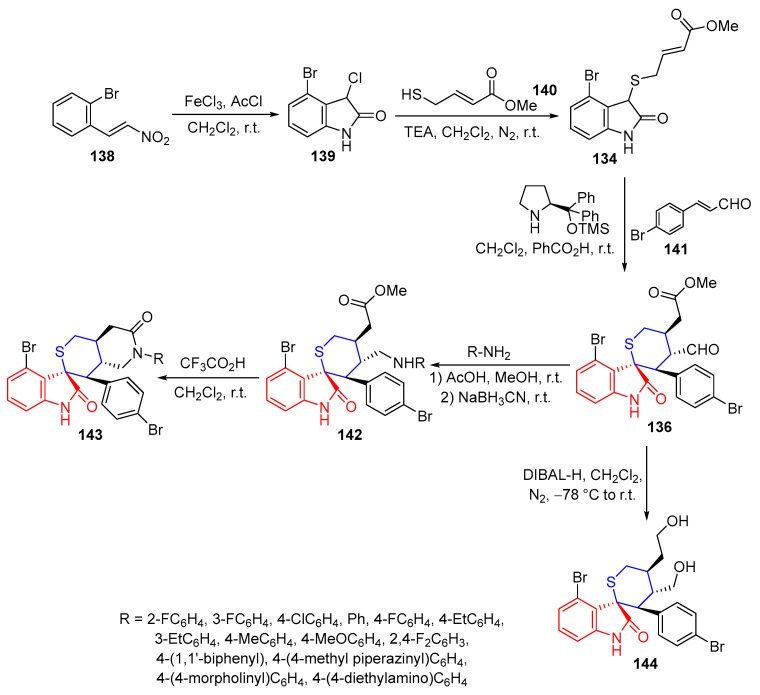
Synthesis of spirooxindole–thiopyrans **136**, **142**–**144**.

The most promising agent discovered in the previous study **136** (R = 4-Br, R’ = 4-BrC_6_H_4_. stating from the appropriate nitroalkene **138** via multi-step reaction sequence) underwent reductive amination (MeOH with two drops of AcOH as a catalyst, followed by the addition of NaBH_3_CN at room temperature), resulting in the corresponding spiro-analogs **142** that underwent intramolecular cyclization due to the influence of CF_3_CO_2_H at room temperature, yielding **143**. Reduction of the aldehydic and ester functional groups of **136** (using diisobutylaluminium hydride “DIBAL-H” in CH_2_Cl_2_ at −78 °C to room temperature under a nitrogen atmosphere) produced the corresponding **144** (Scheme 31). Some synthesized agents displayed significant antiproliferation properties (CCK-8 method) against A549 (lung), HCT116 (colon), and MCF7 (breast) cancer cell lines. Spiro-analog **143** with R = 4-(4-diethylamino)C_6_H_4_ was the most effective agent compared to nutlin-3, exhibiting promising MDM2 inhibitory properties (IC_50_ = 2.9 ± 0.2/10.0 ± 0.8, 1.8 ± 0.5/14.9 ± 0.6, 2.4 ± 0.1/28.4 ± 2.5 μM, KD = 4.2 ± 1.4/0.15 ± 0.04, respectively) (Supplementary Figure S26). The promising agent also demonstrated the induction of apoptosis in A549 cells. Molecular docking (GOLD 5.1, PDB: 4OAS) was employed to elucidate the effectiveness against MDM2. The observations suggested that the synthesized effective agent is a promising lead for optimizing clinically useful anticancer agents through extended pre-clinical and clinical studies [86].

**Scheme 32:**
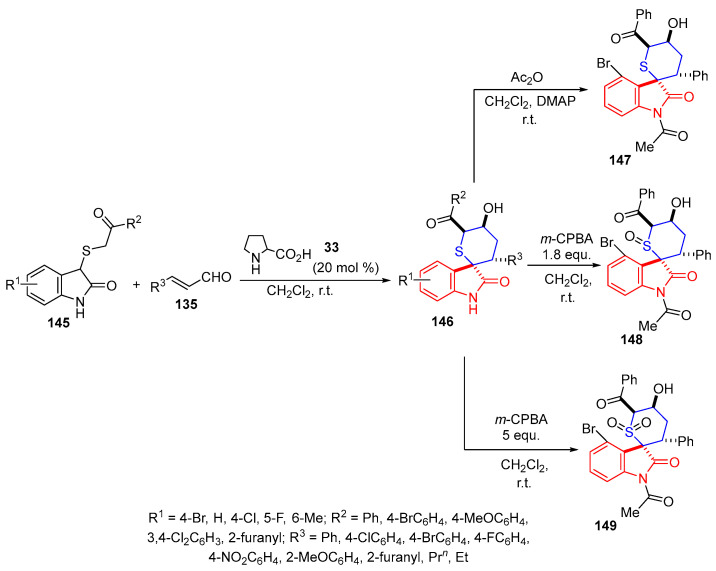
Synthesis of spirooxindole–thiopyrans **146**–**149**.

Additionally, the reaction of 3-[(2-oxo-2-arylethyl)thio]indolin-2-ones **145** with α,β-unsaturated aldehydes **135** in CH_2_Cl_2_ containing proline **33** (20 mol % catalyst) at room temperature underwent a Michael–Aldol reaction, yielding spirooxindole–thiopyrans **146** with good diastereoselectivity. Acylation of these compounds with acetic anhydride in CH_2_Cl_2_ containing DMAP (4-dimethylaminopyridine) at room temperature produced the *N*-acylated analog **147**. Oxidation of **146** with *m*-CPBA (*m*-chloroperoxybenzoic acid, 1.8 and 5.0 mol equivalent) in CH_2_Cl_2_ at room temperature resulted in the corresponding sulfoxide **148** and sulfone **149**, respectively (Scheme 32). X-ray studies confirmed the chemical structures along with their stereochemistry. The synthesized agents **146** with R^1^/R^2^/R^3^ = 4-Br, 4-BrC_6_H_4_, Ph; and **147** displayed promising antiproliferation properties (standard CCK-8 method) against A549 (lung), MCF7 (breast), and HCT116 (colon) cancer cell lines compared to nutlin-3 (% inhibition = 86.9/70.3/42.9, 50.9/77.6/33.1, 81.7/56.4/42.9 at 10 μM, respectively) (Supplementary Figure S27). Although these agents showed promising antiproliferation properties, milder efficacy was observed against MDM2 compared to nutlin-3 (KD = 6.36, 9.68, 0.24 μM, respectively) [87].

**Scheme 33:**
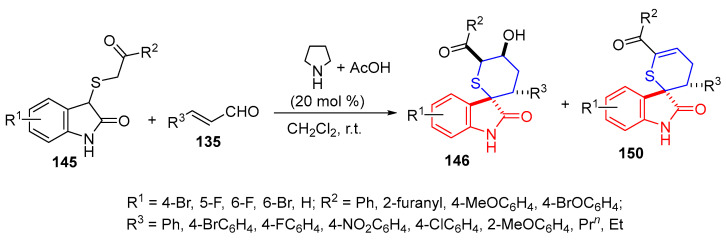
Synthesis of spirooxindole–thiopyrans **144** and **150**.

In another report, the Michael/Aldol reaction involving compounds **145** and **135** was examined, resulting in the formation of spirooxindole–thiopyrans **150**, along with compound **146**. This reaction was performed in the presence of 20 mol% each of pyrrolidine and acetic acid (AcOH) as co-catalytic agents in dichloromethane (CH_2_Cl_2_) for 3 days (refer to Scheme 33). The resulting compound **150** exhibited significant antiproliferative properties, as measured by the standard CCK-8 method, against various carcinoma cell lines, including lung A549, breast MCF7, and colon HCT116. The most promising agent had R^1^ = H, R^2^ = Ph, R^3^ = 4-BrC_6_H_4_, compared to nutlin-3 (% inhibition = 75.1/47.0, 69.4/32.8, 91.9/64.4 at 10 μM; IC_50_ = 2.1/2.4, 6.8/12.1, 1.7/4.0 μM, respectively) (Supplementary Figure S28). It has also been noted that the promising spiro-analog discovered through flow cytometric studies using A549 cells demonstrated cell apoptosis and arrested the cell cycle at the G0/G1 phase similar to nutlin-3. The fluorescence polarization assay showed the MDM2 inhibitory effect of the promising spiro-analog mentioned, with less efficacy than nutlin-3 (K_i_ = 43.8, 0.20 μM, respectively) [88].

## 4. Conclusions

The p53 is a well-known tumor-suppressor protein that controls many cellular functions, including the cell cycle, DNA repair, differentiation, and apoptosis. The essential role of p53 is its ability to conserve genomic stability and prevent mutations during cell division, thereby inhibiting cancer development by arresting the cell cycle and/or inducing apoptosis. The MDM2 protein can regulate or inhibit p53 functions. This is why disrupting the p53–MDM2 interaction appears to be an effective therapeutic strategy. Many chemical scaffolds have been identified with potential p53–MDM2 inhibitory properties; however, spirooxindole-containing compounds hold a unique position in this context. Although no clinical applications have been approved, many analogs have undergone clinical trials and achieved considerable success. Furthermore, many spirooxindoles, when combined with various heterocycles, have shown potential antiproliferative activity against different cancer types and significant inhibitory effects on p53–MDM2. These findings can help optimize novel spirooxindole-based agents as promising candidates for future drug-discovery endeavors. Several spirooxindoles have demonstrated strong anticancer effects independent of wild-type p53, suggesting the involvement of alternative pathways in the apoptosis of cancer cells. Given their unique structures and high efficacy, it is essential to identify the specific signaling pathways responsible for these effects. In this context, spirooxindoles are valuable in the quest for novel hit compounds. Computational techniques can assist in designing targeted agents with exceptional bioproperties, supported by observations from experimental bioproperty studies.

## Data Availability

This published article and its Supplementary Materials files include all data generated or analyzed during this study.

## Supplementary Materials

The following supporting information can be downloaded at: https://www.mdpi.com/article/10.3390/ph18020274/s1, Figure S1: Diastereomeric ratio (dr) values and biological properties of spirooxindole-pyrrolidines **18** and nutlin-3a; Figure S2: Antiproliferation properties of stereoidal spirooxindoles **23** and 5-fluorouracil; Figure S3: *K_i_* values (μM) and cytotoxicity against p53-wild MCF7 (breast) cancer cell line in absence and presence of Fer-1 of spirooxindoles bearing isoxazol-5-yl **26** and nutlin-3; Figure S4: Antiproliferation and MDM2 binding properties of spirooxindoles **36** and 5-fluorouracil; Figure S5: Antiproliferation and MST inhibitory properties of MDM2 of spirooxindoles **39**; Figure S6: Antiproliferation properties of spirooxindoles **47** and nutlin-3; Figure S7: Antiproliferation properties of spirooxindoles **49** and cisplatin; Figure S8: Antiproliferation properties of spirooxindoles linked to 3-acylindole **51** and cisplatin; Figure S9: Antiproliferation properties of dispirooxindole-pyrrolidines **65**–**68** and doxorubicin; Figure S10: Antiproliferation properties of dispirooxindole-pyrrolidines **70** and doxorubicin; Figure S11: Antiproliferation properties of dispirooxindole-pyrrolidines collaborating benzofuranyl heterocycle **74**–**79** and standard references (cisplatin, etoposide, and camptothecin); Figure S12: Antiproliferation properties of dispirooxindole-pyrrolidines **81** and nutlin-3a; Figure S13: Antiproliferation properties of dispirooxindole-pyrrolidines **84**, etoposide, and nutlin-3; Figure S14: Antiproliferation properties of dispirooxindole-pyrrolidines **89**, nutlin-3a and cisplatin; Figure S15: Antiproliferation properties of dispirooxindole-pyrrolidines **92**, nutlin-3a and cisplatin; Figure S16: Antiproliferation properties of spirooxindole-pyrazolines **97**; Figure S17: Antiproliferation properties of spirooxindole-pyrazolines linked to triazolyl heterocycle **101**; Figure S18**:** Antiproliferation properties of spirooxindole-pyrazolines linked to triazolyl heterocycle **104**/**105**; Figure S19: Antiproliferation properties of spirooxindole-isoxazolines **107** and nutlin-3; Figure S20: Antiproliferation properties of spirooxindole-triazoles **108** and nutlin-3a.; Figure S21: Antiproliferation properties of spirooxindole-oxadiazoles **110** and nutlin-3a; Figure S22: Antiproliferation properties and MDM2 inhibitory effect of spirooxindole-piperidines **116**–**119**; Figure S23: Antiproliferation and MDM2 inhibitory properties of spirooxindole-pyrans **123**–**125**; Figure S24: Antiproliferation properties of spirooxindole-benzopyrans **133** and cisplatin; Figure S25: Antiproliferation properties of spirooxindole-thiopyrans **136**, **137** and nutlin-3; Figure S26: Antiproliferation and MDM2 inhibitory properties of spirooxindole-thiopyrans **136**, and **142**–**144**; Figure S27: Antiproliferation properties of spirooxindole-thiopyrans **146**–**149** and nutlin-3; Figure S28: Antiproliferation properties of spirooxindole-thiopyrans **150**, and nutlin-3.

## List of Abbreviations

Apaf-1	Apoptotic peptidase activating factor 1
ATR	Ataxia telangiectasia and Rad3-related protein
BCL2	B-cell lymphoma-2
BiFC	Bimolecular fluorescence complementation
Chk	Serine–threonine checkpoint kinase
Cyt-c	Cytochrome complex
DBU	1,8-Diazabicyclo[5.4.0]undec-7-ene
DCE	Dichloroethane
DIBAL-H	Diisobutylaluminium hydride
DIPEA	*N*,*N*-Diisopropylethylamine
DMAP	4-Dimethylaminopyridine
DMP	Dess–Martin Periodinane
dr	Diastereomeric ratio
Fer-1	Ferrostatin-1
GPX4	Glutathione peroxidase 4
HATU	Hexafluorophosphate azabenzotriazole tetramethyl uranium
i.p.	Intraperitoneal
*m*-CPBA	*m*-Chloroperoxybenzoic acid
MDM2	Murine double minute 2
MST	Microscale thermophoresis
NF-κB	Nuclear factor kappa-light-chain-enhancer
OTFBA	2-(Trifluoromethyl)benzoic acid
PCD	Programmed cell death
PROTAC	Proteolysis targeting chimeras
p-Tos	p-Toluene sulfonic
r.t.	Room temperature
RCD	Regulated cell death
Sc(OTf)_3_	Trifluoromethanesulfonic acid scandium(III) salt
TBAB	Tetrabutylammonium bromide
TEA	Triethylamine
TFA	Trifluoroacetic acid
TNFR	Tumor necrosis factor receptor
Yb(OTf)_3_	Trifluoromethanesulfonic acid ytterbium(III) salt

## References

[1] Peng F., Liao M., Qin R., Zhu S., Peng C., Fu L., Chen Y., Han B. (2022). Regulated cell death (RCD) in cancer: Key pathways and targeted therapies. Signal Transduct. Target. Ther..

[2] Tong X., Tang R., Xiao M., Xu J., Wang W., Zhang B., Liu J., Yu X., Shi S. (2022). Targeting cell death pathways for cancer therapy: Recent developments in necroptosis, pyroptosis, ferroptosis, and cuproptosis research. J. Hematol. Oncol..

[3] Koren E., Fuchs Y. (2021). Modes of regulated cell death in cancer. Cancer Discov..

[4] Pfeffer C.M., Singh A.T.K. (2018). Apoptosis: A Target for anticancer therapy. Int. J. Mol. Sci..

[5] Gollner A., Rudolph D., Arnhof H., Bauer M., Blake S.M., Boehmelt G., Cockroft X.-L., Dahmann G., Ettmayer P., Gerstberger T. (2016). Discovery of novel spiro[3*H*-indole-3,2′-pyrrolidin]-2(1*H*)-one compounds as chemically stable and orally active inhibitors of the MDM2-p53 interaction. J. Med. Chem..

[6] Gupta A.K., Bharadwaj M., Kumar A., Mehrotra R. (2017). Spiro-oxindoles as a promising class of small molecule inhibitors of p53–MDM2 interaction useful in targeted cancer therapy. Top. Curr. Chem..

[7] Li J., Wu Y., Guo Z., Zhuang C., Yao J., Dong G., Yu Z., Min X., Wang S., Liu Y. (2014). Discovery of 1-arylpyrrolidone derivatives as potent p53–MDM2 inhibitors based on molecule fusing strategy. Bioorg. Med. Chem. Lett..

[8] Barcherini V., Almeida J., Lopes E.A., Wang M., Silva D.M., Mori M., Wang S., Saraiva L., Santos M.M.M. (2021). Potency and selectivity optimization of tryptophanol-derived oxazoloisoindolinones: Novel p53 activators in human colorectal cancer. ChemMedChem.

[9] Grigoreva T.A., Novikova D.S., Petukhov A.V., Gureev M.A., Garabadzhiu A.V., Melino G., Barlev N.A., Tribulovich V.G. (2017). Proapoptotic modification of substituted isoindolinones as MDM2-p53 inhibitors. Bioorg. Med. Chem. Lett..

[10] Zhao Y., Yu S., Sun W., Liu L., Lu J., McEachern D., Shargary S., Bernard D., Li X., Zhao T. (2013). A potent small-molecule inhibitor of the MDM2–p53 interaction (MI-888) achieved complete and durable tumor regression in mice. J. Med. Chem..

[11] Zhuang C., Miao Z., Zhu L., Dong G., Guo Z., Wang S., Zhang Y., Wu Y., Yao J., Sheng C. (2012). Discovery, synthesis, and biological evaluation of orally active pyrrolidone derivatives as novel inhibitors of p53-MDM2 protein-protein interaction. J. Med. Chem..

[12] Wang Y., Ji B., Cheng Z., Zhang L., Cheng Y., Li Y., Ren J., Liu W., Ma Y. (2022). Synthesis and biological evaluation of novel synthetic indolone derivatives as anti-tumor agents targeting p53-MDM2 and p53-MDMX. Molecules.

[13] Ivanenkov Y.A., Kukushkin M.E., Beloglazkina A.A., Shafikov R.R., Barashkin A.A., Ayginin A.A., Serebryakova M.S., Majouga A.G., Skvortsov D.A., Tafeenko V.A. (2023). Synthesis and biological evaluation of novel dispiro-indolinones with anticancer activity. Molecules.

[14] Panda S.S., Girgis A.S., Aziz M.N., Bekheit M.S. (2023). Spirooxindole: A versatile biologically active heterocyclic scaffold. Molecules.

[15] Xie X., Xiong S.-S., Li X., Huang H., Wu F.-B., Shen P.-F., Peng C., He G., Han B. (2021). Design and organocatalytic synthesis of spirooxindole-cyclopentene-isoxazole hybrids as novel MDM2–p53 inhibitors. Org. Chem. Front..

[16] Aboshouk D.R., Youssef M.A., Bekheit M.S., Hamed A.R., Girgis A.S. (2024). Antineoplastic indole-containing compounds with potential VEGFR inhibitory properties. RSC Adv..

[17] Kozielewicz P., Paradowska K., Erić S., Wawer I., Zloh M. (2014). Insights into mechanism of anticancer activity of pentacyclic oxindole alkaloids of *Uncaria tomentosa* by means of a computational reverse virtual screening and molecular docking approach. Monatsh. Chem..

[18] Bekheit M.S., Panda S.S., Kariuki B.M., Mahmoud S.H., Mostafa A., Girgis A.S. (2023). Spiroindole-containing compounds bearing phosphonate group of potential M^pro^-SARS-CoV-2 inhibitory properties. Eur. J. Med. Chem..

[19] Fawazy N.G., Panda S.S., Mostafa A., Kariuki B.M., Bekheit M.S., Moatasim Y., Kutkat O., Fayad W., El-Manawaty M.A., Soliman A.A.F. (2022). Development of spiro-3-indolin-2-one containing compounds of antiproliferative and anti-SARS-CoV-2 properties. Sci. Rep..

[20] Youssef M.A., Panda S.S., El-Shiekh R.A., Shalaby E.M., Aboshouk D.R., Fayad W., Fawzy N.G., Girgis A.S. (2020). Synthesis and molecular modeling studies of cholinesterase inhibitor dispiro[indoline-3,2′-pyrrolidine-3′,3″-pyrrolidines]. RSC Adv..

[21] Pandey A., Pandey A., Dubey R., Kant R., Pandey J. (2022). Synthesis and computational studies of potent antimicrobial and anticancer indolone scaffolds with spiro cyclopropyl moiety as a novel design element. J. Indian Chem. Soc..

[22] Salem M.A., Ragab A., Askar A.A., El-Khalafawy A., Makhlouf A.H. (2020). One-pot synthesis and molecular docking of some new spiropyranindol-2-one derivatives as immunomodulatory agents and in vitro antimicrobial potential with DNA gyrase inhibitor. Eur. J. Med. Chem..

[23] Jarrahpour A., Jowkar Z., Haghighijoo Z., Heiran R., Rad J.A., Sinou V., Rouvier F., Latour C., Brunel J.M., Őzdemir N. (2022). Synthesis, in-vitro biological evaluation, and molecular docking study of novel spiro-β-lactam-isatin hybrids. Med. Chem. Res..

[24] Bolous M., Arumugam N., Almansour A.I., Kumar R.S., Maruoka K., Antharam V.C., Thangamani S. (2019). Broad-spectrum antifungal activity of spirooxindolo-pyrrolidine tethered indole/imidazole hybrid heterocycles against fungal pathogens. Bioorg. Med. Chem. Lett..

[25] Moghaddam-Manesh M., Sheikhhosseini E., Ghazanfari D., Akhgar M. (2020). Synthesis of novel 2-oxospiro[indoline-3,4′-[1,3]dithiine]-5′-carbonitrile derivatives by new spiro[indoline-3,4′-[1,3]dithiine]@Cu(NO_3_)_2_ supported on Fe_3_O_4_@gly@CE MNPs as efficient catalyst and evaluation of biological activity. Bioorg. Chem..

[26] Lotfy G., El Ashry E.H., Said M.M., El Tamany E., Aziz Y.M.A., Al-Dhfyan A., Al-Majid A.M., Barakat A. (2018). Regio- and stereoselective synthesis of new spirooxindoles via 1,3-dipolar cycloaddition reaction: Anticancer and molecular docking studies. J. Photochem. Photobiol. B Biol..

[27] Gao X., Liu J., Cho K.B., Kedika S., Guo B. (2020). Chemopreventive agent 3,3’-diindolylmethane inhibits MDM2 in colorectal cancer cells. Int. J. Mol. Sci..

[28] Yu B., Yu D.-Q., Liu H.-M. (2015). Spirooxindoles: Promising scaffolds for anticancer agents. Eur. J. Med. Chem..

[29] Dong H., Song S., Li J., Xu C., Zhang H., Ouyang L. (2015). The discovery of oxazolones-grafted spirooxindoles via three-component diversity oriented synthesis and their preliminary biological evaluation. Bioorg. Med. Chem. Lett..

[30] Beloglazkina A., Barashkin A., Polyakov V., Kotovsky G., Karpov N., Mefedova S., Zagribelny B., Ivanenkov Y., Kalinina M., Skvortsov D. (2020). Synthesis and biological evaluation of novel dispiro compounds based on 5-arylidenehydantoins and isatins as inhibitors of p53–MDM2 protein–protein interaction. Chem. Heterocycl. Compd..

[31] Nafie M.S., Al-Majid A.M., Ali M., Alayyaf A.A., Haukka M., Ashraf S., Ul-Haq Z., El-Faham A., Barakat A. (2024). Exploring pyrrolidinyl-spirooxindole natural products as promising platforms for the synthesis of novel spirooxindoles as EGFR/CDK2 inhibitors for halting breast cancer cells. Front. Chem..

[32] Girgis A.S., Panda S.S., Aziz M.N., Steel P.J., Hall C.D., Katritzky A.R. (2015). Rational design, synthesis, and 2D-QSAR study of anti-oncological alkaloids against hepatoma and cervical carcinoma. RSC Adv..

[33] Huang Y., Jiao Z., Fu Y., Hou Y., Sun J., Hu F., Yu S., Gong K., Liu Y., Zhao G. (2024). An overview of the functions of p53 and drugs acting either on wild- or mutant-type p53. Eur. J. Med. Chem..

[34] Chahat, Bhatia R., Kumar B. (2023). p53 as a potential target for treatment of cancer: A perspective on recent advancements in small molecules with structural insights and SAR studies. Eur. J. Med. Chem..

[35] Bora D., Kaushal A., Shanka N. (2021). Anticancer potential of spirocompounds in medicinal chemistry: A pentennial expedition. Eur. J. Med. Chem..

[36] Santos M.M.M. (2014). Recent advances in the synthesis of biologically active spirooxindoles. Tetrahedron.

[37] Li R., Wu Y., Li Y., Shuai W., Wang A., Zhu Y., Hu X., Xia Y., Ouyang L., Wang G. (2024). Targeted regulated cell death with small molecule compounds in colorectal cancer: Current perspectives of targeted therapy and molecular mechanisms. Eur. J. Med. Chem..

[38] Siegel R.L., Giaquinto A.N., Jemal A. (2024). Cancer statistics, 2024. CA Cancer J. Clin..

[39] Bray F., Laversanne M., Sung H., Ferlay J., Siegel R.L., Soerjomataram I., Jemal A. (2024). Global cancer statistics 2022: GLOBOCAN estimates of incidence and mortality worldwide for 36 cancers in 185 countries. CA Cancer J. Clin..

[40] Nutlin-3: Uses, Interactions, Mechanism of Action|Drugbank Online. https://go.drugbank.com/drugs/DB17039.

[41] U.S. National Library of Medicine RO-5045337. National Center for Biotechnology Information. PubChem Compound Database. https://pubchem.ncbi.nlm.nih.gov/compound/rg7112.

[42] Wang S., Sun W., Zhao Y., McEachern D., Meaux I., Barrière C., Stuckey J., Meagher J., Bai L., Liu L. (2014). SAR405838: An optimized inhibitor of MDM2-p53 interaction that induces complete and durable tumor regression. Cancer Res..

[43] Bill K.L.J., Garnett J., Meaux I., Ma X., Creighton C.J., Bolshakov S., Barriere C., Debussche L., Lazar A.J., Prudner B.C. (2016). SAR405838: A novel and potent inhibitor of the MDM2:p53 axis for the treatment of dedifferentiated liposarcoma. Clin. Cancer Res..

[44] de Weger V.A., de Jonge M., Langenberg M.H.G., Schellens J.H.M., Lolkema M., Varga A., Demers B., Thomas K., Hsu K., Tuffal G. (2019). A phase I study of the HDM2 antagonist SAR405838 combined with the MEK inhibitor pimasertib in patients with advanced solid tumours. Br. J. Cancer.

[45] Fang D.D., Tang Q., Kong Y., Rong T., Wang Q., Li N., Fang X., Gu J., Xiong D., Yin Y. (2021). MDM2 inhibitor APG-115 exerts potent antitumor activity and synergizes with standard-of-care agents in preclinical acute myeloid leukemia models. Cell Death Discov..

[46] Fang D.D., Tang Q., Kong Y., Wang Q., Gu J., Fang X., Zou P., Rong T., Wang J., Yang D. (2019). MDM2 inhibitor APG-115 synergizes with PD-1 blockade through enhancing antitumor immunity in the tumor microenvironment. J. Immunother. Cancer.

[47] Yi H., Yan X., Luo Q., Yuan L., Li B., Pan W., Zhang L., Chen H., Wang J., Zhang Y. (2018). A novel small molecule inhibitor of MDM2-p53 (APG-115) enhances radiosensitivity of gastric adenocarcinoma. J. Exp. Clin. Cancer Res..

[48] U.S. National Library of Medicine Idasanutlin. National Center for Biotechnology Information. PubChem Compound Database. https://pubchem.ncbi.nlm.nih.gov/compound/Idasanutlin.

[49] Idasanutlin: Uses, Interactions, Mechanism of Action|Drugbank Online. https://go.drugbank.com/drugs/DB12325.

[50] Arnhold V., Schmelz K., Proba J., Winkler A., Wünschel J., Toedling J., Deubzer H.E., Künkele A., Eggert A., Schulte J.H. (2018). Reactivating TP53 signaling by the novel MDM2 inhibitor DS- 3032b as a therapeutic option for high-risk neuroblastoma. Oncotarget.

[51] Senapati J., Muftuoglu M., Ishizawa J., Abbas H.A., Loghavi S., Borthakur G., Yilmaz M., Issa G.C., Dara S.I., Basyal M. (2023). A Phase I study of Milademetan (DS3032b) in combination with low dose cytarabine with or without venetoclax in acute myeloid leukemia: Clinical safety, efficacy, and correlative analysis. Blood Cancer J..

[52] Akaev A.A., Bezzubov S.I., Desyatkin V.G., Vorobyeva N.S., Majouga A.G., Melnikov M.Y., Budynina E.M. (2019). Stereocontrolled [3+2] cycloaddition of donor-acceptor cyclopropanes to iminooxindoles: Access to spiro[oxindole-3,2’-pyrrolidines]. J. Org. Chem..

[53] Yu B., Sun X.-N., Shi X.-J., Qi P.-P., Zheng Y.-C., Yu D.-Q., Liu H.-M. (2015). Efficient synthesis of novel antiproliferative steroidal spirooxindoles via the [3+2] cycloaddition reactions of azomethine ylides. Steroids.

[54] Liu S.-J., Zhao Q., Peng C., Mao Q., Wu F., Zhang F.-H., Feng Q.-S., He G., Han B. (2021). Design, synthesis, and biological evaluation of nitroisoxazole-containing spiro[pyrrolidin-oxindole] derivatives as novel glutathione peroxidase 4/mouse double minute 2 dual inhibitors that inhibit breast adenocarcinoma cell proliferation. Eur. J. Med. Chem..

[55] Aziz Y.M.A., Lotfy G., Said M.M., El Ashry E.H., El Tamany E.H., Soliman S.M., Abu-Serie M.M., Teleb M., Yousuf S., Dömling A. (2021). Design, synthesis, chemical and biochemical insights into novel hybrid spirooxindole-based p53-MDM2 inhibitors With potential Bcl2 signaling attenuation. Front. Chem..

[56] Lotfy G., Aziz Y.M.A., Said M.M., El Ashry E.H., El Tamany E.H., Abu-Serie M.M., Teleb M., Dömling A., Barakat A. (2021). Molecular hybridization design and synthesis of novel spirooxindole-based MDM2 inhibitors endowed with *BCL2* signaling attenuation; a step towards the next generation p53 activators. Bioorg. Chem..

[57] Barakat A., Alshahrani S., Al-Majid A.M., Alamary A.S., Haukka M., Abu-Serie M.M., Dömling A., Mazyed E.A., Badria F.A., El-Senduny F.F. (2022). Novel spirooxindole based benzimidazole scaffold: In vitro, nanoformulation and in vivo studies on anticancer and antimetastatic activity of breast adenocarcinoma. Bioorg. Chem..

[58] Alshahrani S., Al-Majid A.M., Ali M., Alamary A.S., Abu-Serie M.M., Dömling A., Shafiq M., Ul-Haq Z., Barakat A. (2023). Rational design, synthesis, separation, and characterization of new spiroxindoles combined with benzimidazole scaffold as an MDM2 inhibitor. Separations.

[59] Cui J., Wang Y., Li X., Xiao F., Ren H., Wu M. (2023). Synthesis and antineoplastic activity of a dimer, Spiroindolinone pyrrolidinecarboxamide. Molecules.

[60] Kumar A., Gupta G., Bishnoi A.K., Saxena R., Saini K.S., Konwar R., Kumar S., Dwivedi A. (2015). Design and synthesis of new bioisosteres of spirooxindoles (MI-63/219) as anti-breast cancer agents. Bioorg. Med. Chem..

[61] Barakat A., Islam M.S., Ghawas H.M., Al-Majid A.M., El-Senduny F.F., Badria F.A., Elshaier Y.A.M.M., Ghabbour H.A. (2019). Design and synthesis of new substituted spirooxindoles as potential inhibitors of the MDM2–p53 interaction. Bioorg. Chem..

[62] Islam M.S., Ghawas H.M., El-Senduny F.F., Al-Majid A.M., Elshaier Y.A.M.M., Badria F.A., Barakat A. (2019). Synthesis of new thiazolo-pyrrolidine-(spirooxindole) tethered to 3-acylindole as anticancer agents. Bioorg. Chem..

[63] Gollner A., Weinstabl H., Fuchs J.E., Rudolph D., Garavel G., Hofbauer K.S., Oezguer J.K., Gmaschitz G., Hela W., Kerres N. (2019). Targeted synthesis of complex spiro[3*H*-indole-3,2’-pyrrolidin]-2(1*H*)-ones by intramolecular cyclization of azomethine ylides: Highly potent MDM2–p53 inhibitors. ChemMedChem.

[64] Girgis A.S., Panda S.S., Srour A.M., Farag H., Ismail N.S.M., Elgendy M., Abdel-Aziz A.K., Katritzky A.R. (2015). Rational design, synthesis and molecular modeling studies of novel anti-oncological alkaloids against melanoma. Org. Biomol. Chem..

[65] Girgis A.S., Panda S.S., Shalaby E.M., Mabied A.F., Steel P.J., Hall C.D., Katritzky A.R. (2015). Regioselective synthesis and DFT study of an anti-neoplastic fluoro-substituted dispiro-oxindole. RSC Adv..

[66] Shalaby E.M., Girgis A.S., Moustafa A.M., ElShaabiny A.M., El-Gendy B.E.M., Mabied A.F., Farag I.S.A. (2014). Regioselective synthesis, stereochemical structure, spectroscopic characterization and geometry optimization of dispiro[3H-indole-3,2′-pyrrolidine-3′,3″-piperidines]. J. Mol. Struct..

[67] Shanmugasundaram P., Easwaramoorthy D., Mhashal A.R., Khan A. (2023). Design and synthesis of densely functionalized novel spirooxindoles as anticancer agents. Rasayan J. Chem..

[68] Al-Majid A.M., Ali M., Islam M.S., Alshahrani S., Alamary A.S., Yousuf S., Choudhary M.I., Barakat A. (2021). Stereoselective synthesis of the di-spirooxindole analogs based oxindole and cyclohexanone moieties as potential anticancer agents. Molecules.

[69] Sutariya T.R., Brahmbhatt G.C., Atara H.D., Parmar N.J., RajniKant, Gupta V.K., Lagunes I., Padrón J.M., Murumkar P.R., Sharma M.K. (2023). An efficient one-pot synthesis and docking studies of bioactive new antiproliferative dispiro[oxindole/acenaphthylenone–benzofuranone] pyrrolidine scaffolds. Mol. Divers..

[70] Kukushkin M., Novotortsev V., Filatov V., Ivanenkov Y., Skvortsov D., Veselov M., Shafikov R., Moiseeva A., Zyk N., Majouga A. (2021). Synthesis and biological evaluation of S-, O- and Se-containing dispirooxindoles. Molecules.

[71] Kukushkin M.E., Skvortsov D.A., Kalinina M.A., Tafeenko V.A., Burmistrov V.V., Butov G.M., Zyk N.V., Majouga A.G., Beloglazkina E.K. (2020). Synthesis and cytotoxicity of oxindoles dispiro derivatives with thiohydantoin and adamantane fragments. Phosphorus Sulfur Silicon Relat. Elem..

[72] Meenakshy C.B., Sudheendranath A., Thomas N.V., Asha V.S., Biju P.G., Deepthi A. (2024). One-pot synthesis, structural study, in silico and in vitro anticancer evaluation of spiropyrrolidine oxindoles obtained by the 3-CR of isatin, benzyl amine and a thiazolo[3,2-*a*]indole derivative. J. Mol. Struct..

[73] Nunes R.C., Ribeiro C.J.A., Monteiro Ȃ., Rodrigues C.M.P., Amaral J.D., Santos M.M.M. (2017). In vitro targeting of colon cancer cells using spiropyrazoline oxindoles. Eur. J. Med. Chem..

[74] Girst G., Lopes E.A., Gonçalves L.M., Espadinha M., Kúsz N., Wang H.-C., Santos M.M.M., Hunyadi A. (2023). Hybrid molecules of protoflavones and spirooxindole derivatives with selective cytotoxicity against triple-negative breast cancer cells. RSC Med. Chem..

[75] Bokhtia R.M., Panda S.S., Girgis A.S., Samir N., Said M.F., Abdelnaser A., Nasr S., Bekheit M.S., Dawood A.S., Sharma H. (2023). New NSAID conjugates as potent and selective COX-2 inhibitors: Synthesis, molecular modeling and biological investigation. Molecules.

[76] Youssef M.A., Panda S.S., Aboshouk D.R., Said M.F., El Taweel A., GabAllah M., Fayad W., Soliman A.A.F., Mostafa A., Fawzy N.G. (2022). Novel curcumin mimics: Design, synthesis, biological properties and computational studies of piperidone-piperazine conjugates. ChemistrySelect.

[77] Girgis A.S., Kalmouch A., Ellithey M. (2006). Synthesis of novel vasodilatory active nicotinate esters with amino acid function. Bioorg. Med. Chem..

[78] Ribeiro C.J.A., Amaral J.D., Rodrigues C.M.P., Moreira R., Santos M.M.M. (2014). Synthesis and evaluation of spiroisoxazoline oxindoles as anticancer agents. Bioorg. Med. Chem..

[79] Ribeiro C.J.A., Nunes R.C., Amaral J.D., Gonçalves L.M., Rodrigues C.M.P., Moreira R., Santos M.M.M. (2017). Spirotriazoline oxindoles: A novel chemical scaffold with in vitro anticancer properties. Eur. J. Med. Chem..

[80] Ribeiro C.J.A., Amaral J.D., Rodrigues C.M.P., Moreira R., Santos M.M.M. (2016). Spirooxadiazoline oxindoles with promising in vitro antitumor activities. Med. Chem. Commun..

[81] Yang M.-C., Peng C., Huang H., Yang L., He X.-H., Huang W., Cui H.-L., He G., Han B. (2017). Organocatalytic asymmetric synthesis of spiro-oxindole piperidine derivatives that reduce cancer cell proliferation by inhibiting MDM2−p53 interaction. Org. Lett..

[82] Mu J., Xie X., Xiong S., Zhang Y., Wang Y., Zhao Q., Zhu H., Huang W., He G. (2021). Discovery of spirooxindole–ferrocene hybrids as novel MDM2 inhibitors. Chin. Chem. Lett..

[83] Zhang W.-H., Chen S., Liu X.-L., Bing-Lina, Liu X.-W., Zhou Y. (2020). Study on antitumor activities of the chrysin-chromene-spirooxindole on Lewis lung carcinoma C57BL/6 mice in vivo. Bioorg. Med. Chem. Lett..

[84] Zhang L., Ren W., Wang X., Zhang J., Liu J., Zhao L., Zhang X. (2017). Discovery of novel polycyclic spiro-fused carbocyclicoxindole-based anticancer agents. Eur. J. Med. Chem..

[85] Wang S., Jiang Y., Wu S., Dong G., Miao Z., Zhang W., Sheng C. (2016). Meeting organocatalysis with drug discovery: Asymmetric synthesis of 3,3′-spirooxindoles fused with tetrahydrothiopyrans as novel p53-MDM2 inhibitors. Org. Lett..

[86] Ji C., Wang S., Chen S., Jiang S.H.Y., Miao Z., Sheng C. (2017). Design, synthesis and biological evaluation of novel antitumor spirotetrahydrothiopyran–oxindole derivatives as potent p53-MDM2 inhibitors. Bioorg. Med. Chem..

[87] Wang S., Chen S., Guo Z., He S., Zhang F., Liu X., Chen W., Zhang S., Sheng C. (2018). Synthesis of spiro-tetrahydrothiopyran-oxindoles by Michael–aldol cascade reactions: Discovery of potential P53-MDM2 inhibitors with good antitumor activity. Org. Biomol. Chem..

[88] Liu W., Chen S., Zhang F., He S., Wang S., Sheng C. (2019). Design, synthesis and biological evaluation of novel antitumor spirodihydrothiopyran-oxindole derivatives. Bioorg. Med. Chem. Lett..

