# Supplementary material for: The Therapeutic Potential of Spirooxindoles in Cancer: A Focus on p53–MDM2 Modulation
Source: Pharmaceuticals (Basel). 2025 Feb 19;18(2):274. doi: 10.3390/ph18020274 (PMC11859340; doi:10.3390/ph18020274)
Supplement: Supplementary file 1 [file pharmaceuticals-18-00274-s001.zip › pharmaceuticals-3398217-supplementary.pdf]

Supplementary Material

The Therapeutic Potential of Spirooxindoles in Cancer: A Focus on p53–MDM2 Modulation

Adel S. Girgis [1,*], Yujun Zhao [2], Angel Nkosi [3], Nasser S. M. Ismail [4], Mohamed S. Bekheit [1], Dalia R. Aboshouk [1], Marian N. Aziz [1], M. Adel Youssef [5] and Siva S. Panda [3,6,*]

[1] Department of Pesticide Chemistry, National Research Centre, Dokki, Giza 12622, Egypt; m_bekheit@yahoo.com (M.S.B.); daliaraslan205@yahoo.com (D.R.A.); mariannady97@yahoo.com (M.N.A.)

[2] State Key Laboratory of Drug Research and Small-Molecule Drug Research Center, Shanghai Institute of Materia Medica, Chinese Academy of Sciences, 555 Zuchongzhi Rd, Shanghai 201203, China; yjzhao@simm.ac.cn

[3] Department of Chemistry and Biochemistry, Augusta University, Augusta, GA 30912, USA; ankosi@augusta.edu

[4] Pharmaceutical Chemistry Department, Faculty of Pharmacy, Ain Shams University, Cairo 11566, Egypt; saadnasser2003@yahoo.com

[5] Department of Chemistry, Faculty of Science, Helwan University, Helwan 11795, Egypt; adelgirgis100@gmail.com

[6] Department of Biochemistry and Molecular Biology, Augusta University, Augusta, GA 30912, USA

* Correspondence: as.girgis@nrc.sci.eg or girgisas10@yahoo.com (A.S.G.); sspanda12@gmail.com or sipanda@augusta.edu (S.S.P.)

Figure captions

Figure S1: Diastereomeric ratio (dr) values and biological properties of spirooxindole-pyrrolidines **18** and nutlin-3a.

Figure S2: Antiproliferation properties of stereoidal spirooxindoles **23** and 5-fluorouracil.

Figure S3: *K_i* values (μM) and cytotoxicity against p53-wild MCF7 (breast) cancer cell line in absence and presence of Fer-1 of spirooxindoles bearing isoxazol-5-yl **26** and nutlin-3.

Figure S4: Antiproliferation and MDM2 binding properties of spirooxindoles **36** and 5-fluorouracil.

Figure S5: Antiproliferation and MST inhibitory properties of MDM2 of spirooxindoles **39**.

Figure S6: Antiproliferation properties of spirooxindoles **47** and nutlin-3.

Figure S7: Antiproliferation properties of spirooxindoles **49** and cisplatin.

Figure S8: Antiproliferation properties of spirooxindoles linked to 3-acylindole **51** and cisplatin.

Figure S9: Antiproliferation properties of dispirooxindole-pyrrolidines **65-68** and doxorubicin.

Figure S10: Antiproliferation properties of dispirooxindole-pyrrolidines **70** and doxorubicin.

Figure S11: Antiproliferation properties of dispirooxindole-pyrrolidines collaborating benzofuranyl heterocycle **74-79** and standard references (cisplatin, etoposide, and camptothecin).

Figure S12: Antiproliferation properties of dispirooxindole-pyrrolidines **81** and nutlin-3a.

Figure S13: Antiproliferation properties of dispirooxindole-pyrrolidines **84**, etoposide, and nutlin-3.

Figure S14: Antiproliferation properties of dispirooxindole-pyrrolidines **89**, nutlin-3a and cisplatin.

Figure S15: Antiproliferation properties of dispirooxindole-pyrrolidines **92**, nutlin-3a and cisplatin.

FigureS16: Antiproliferation properties of spirooxindole-pyrazolines **97**.

Figure S17: Antiproliferation properties of spirooxindole-pyrazolines linked to triazolyl heterocycle **101**.

Figure S18: Antiproliferation properties of spirooxindole-pyrazolines linked to triazolyl heterocycle **104/105**.

Figure S19: Antiproliferation properties of spirooxindole-isoxazolines **107** and nutlin-3.

Figure S20; Antiproliferation properties of spirooxindole-triazoles **108** and nutlin-3a.

Figure S21: Antiproliferation properties of spirooxindole-oxadiazoles **110** and nutlin-3a.

Figure S22: Antiproliferation properties and MDM2 inhibitory effect of spirooxindole-piperidines **116–119**.

Figure S23: Antiproliferation and MDM2 inhibitory properties of spirooxindole-pyrans **123–125**.

Figure S24: Antiproliferation properties of spirooxindole-benzopyrans **133** and cisplatin.

Figure S25: Antiproliferation properties of spirooxindole-thiopyrans **136**, **137** and nutlin-3.

Figure S26: Antiproliferation and MDM2 inhibitory properties of spirooxindole-thiopyrans **136**, and **142-144**.

Figure S27: Antiproliferation properties of spirooxindole-thiopyrans **146–149** and nutlin-3.

Figure S28: Antiproliferation properties of spirooxindole-thiopyrans **150**, and nutlin-3.

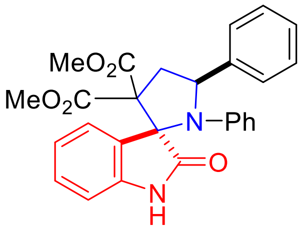

18a
dr = 93:7

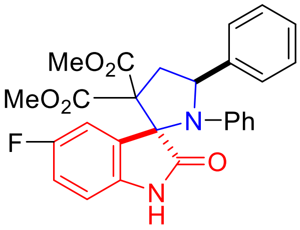

18b
dr = 94:6

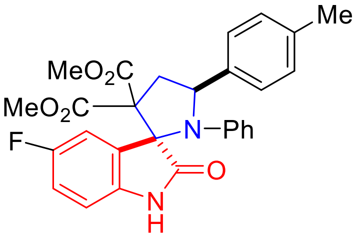

18c
dr = 94:6

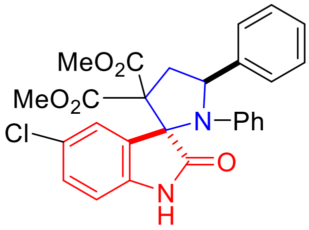

18d
dr = 94:6

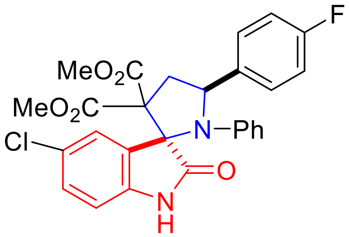

18e
dr = 93:7

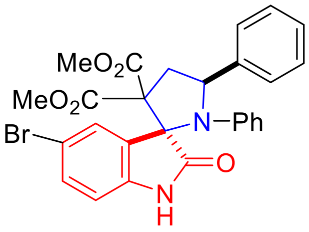

18f
dr = 94:6

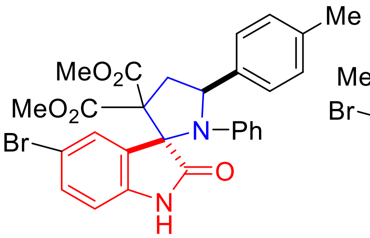

18g
dr = 93:7

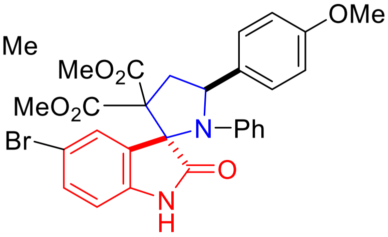

18h
dr = 95:5

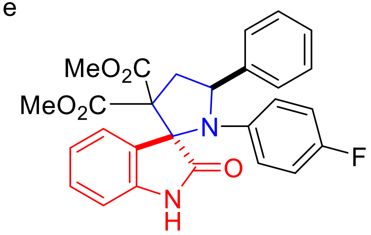

18i
dr = 94:6
IC_{50} = 8.2–9.3, 6.2–8.3 μM
against against PC-3(p53^{-})
and LNCaP (p53^{+}), respectively

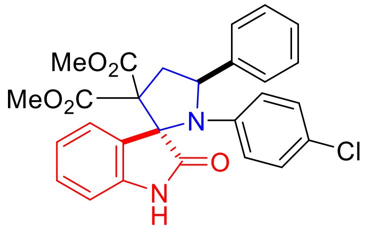

18j
dr = 94:6
IC_{50} = >100, 6.3–7.4 μM
against against PC-3(p53^{-})
and LNCaP (p53^{+}), respectively

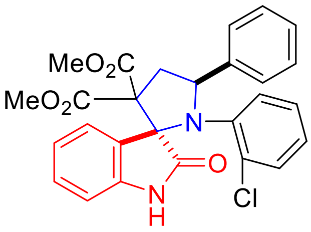

18k
dr = 95:5

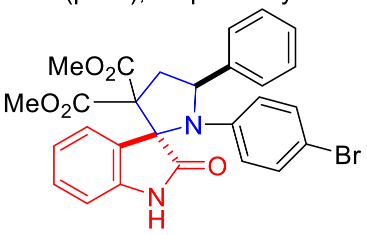

18l
dr = 94:6

Fig. S1. Diastereomeric ratio (dr) values and biological properties of spirooxindole-pyrrolidines **18** and nutlin-3a.

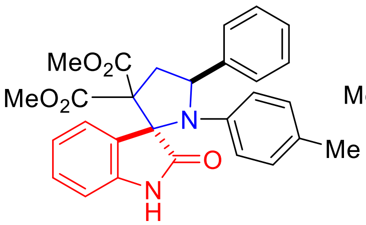

18m

dr = 93:7

IC_{50} = >50, >20 μM against against PC-3(p53⁻) and LNCaP (p53⁺), respectively

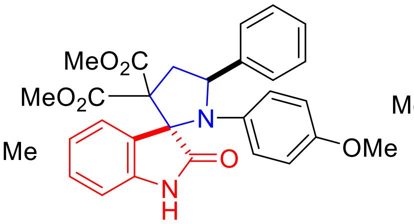

18n

dr = 92:8

IC_{50} = >50, >20 μM against against PC-3(p53⁻) and LNCaP (p53⁺), respectively

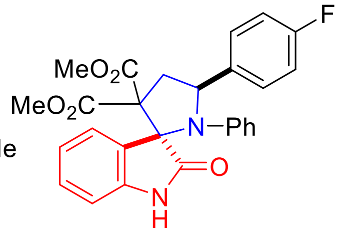

18o

dr = 91:9

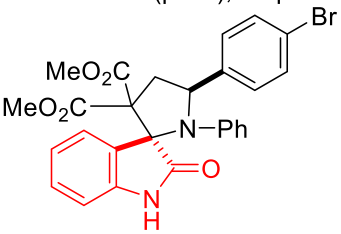

18p

dr = 91:9

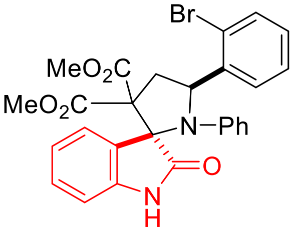

18q

dr = 91:9

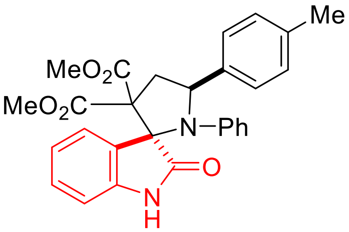

18r

dr = 93:7

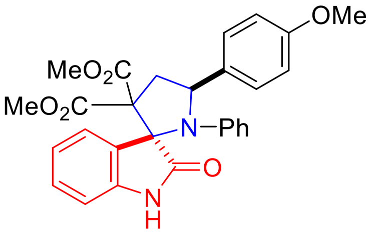

18s

dr = 93:7

IC_{50} = >100, 18–20 μM against against PC-3(p53⁻) and LNCaP (p53⁺), respectively

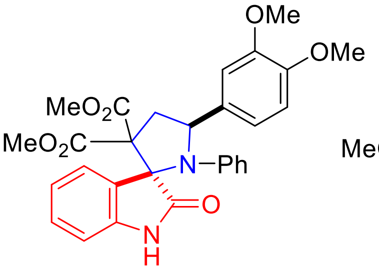

18t

dr = 93:7

IC_{50} = 18–20, 18–20 μM against against PC-3(p53⁻) and LNCaP (p53⁺), respectively

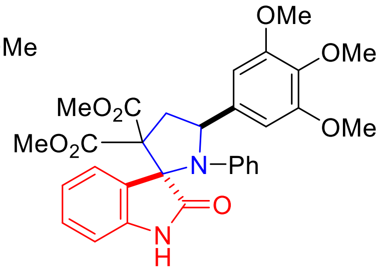

18u

dr = 93:7

IC_{50} = 17–20, 17–20 μM against against PC-3(p53⁻) and LNCaP (p53⁺), respectively

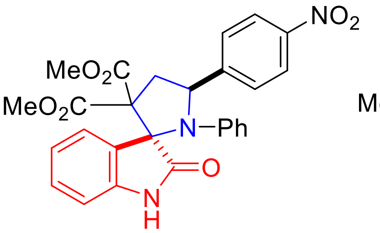

18v

dr = 91:9

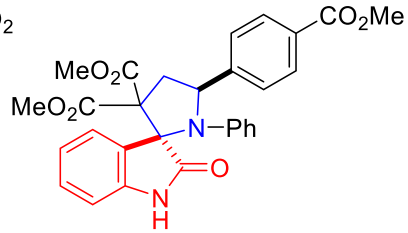

18w

dr = 92:8

IC_{50} = >50, >20 μM against against PC-3(p53⁻) and LNCaP (p53⁺), respectively

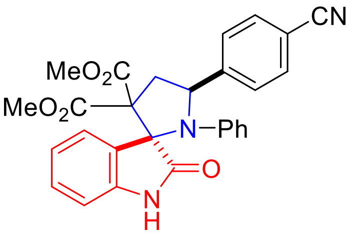

18x

dr = 91:9

IC_{50} = >100, 18–20 μM against against PC-3(p53⁻) and LNCaP (p53⁺), respectively

Fig. S1 (continued)**.** Diastereomeric ratio (dr) values and biological properties of spirooxindole-pyrrolidines **18** and nutlin-3a.

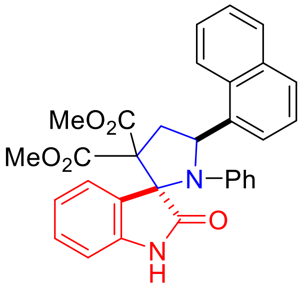

18y

dr = 93:7

IC_{50} = 13–16, 6.7–8.2 μM against against PC-3($p53^{-}$) and LNCaP ($p53^{+}$), respectively

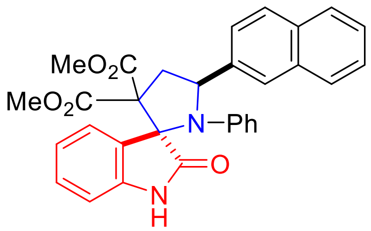

18z

dr = 93:7

IC_{50} = 9.8–11.2, 7.9–8.9 μM against against PC-3($p53^{-}$) and LNCaP ($p53^{+}$), respectively

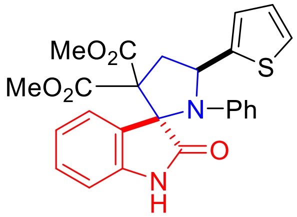

18aa

dr = 91:9

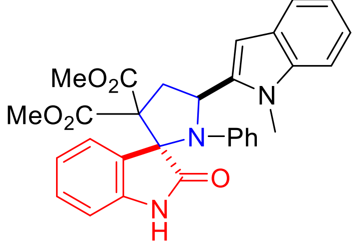

18ab

dr = 93:7

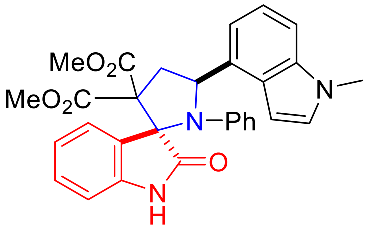

18ac

dr = 93:7

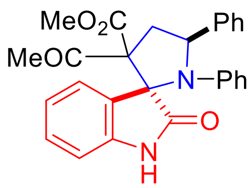

18ad

dr = 60:40

IC_{50} = >20, >20 μM against against PC-3($p53^{-}$) and LNCaP ($p53^{+}$), respectively

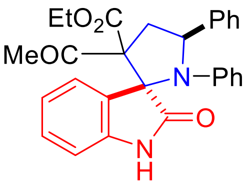

18ae

dr = 63:37

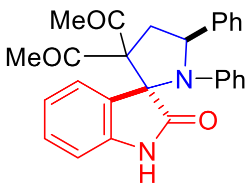

18af

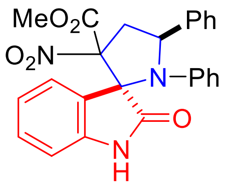

18ag

dr = 80:20

IC_{50} = 6.8–8.5, 6.2–7.9 μM against against PC-3($p53^{-}$) and LNCaP ($p53^{+}$), respectively

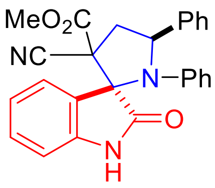

18ah

dr = 64:36

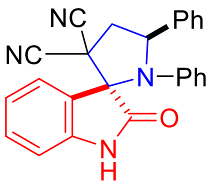

18ai

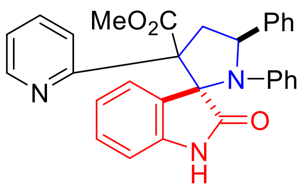

18aj

dr = 71:29

Fig. S1 (continued). Diastereomeric ratio (dr) values and biological properties of spirooxindole-pyrrolidines **18** and nutlin-3a.

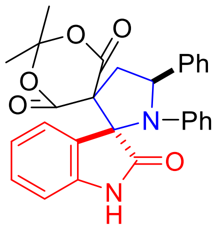

18ak

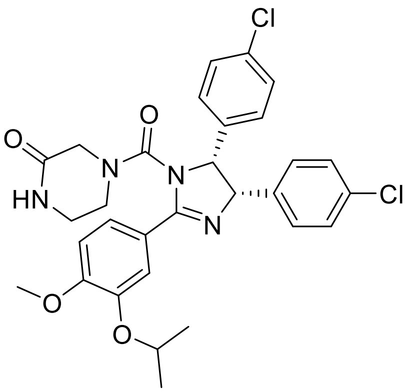

Nutlin-3a

IC_{50} = 28.1–29.6, 2.4–2.8 μM
against against PC-3(p53^{-})
and LNCaP (p53^{+}), respectively

Fig. S1 (continued). Diastereomeric ratio (dr) values and biological properties of spirooxindole-pyrrolidines **18** and nutlin-3a.

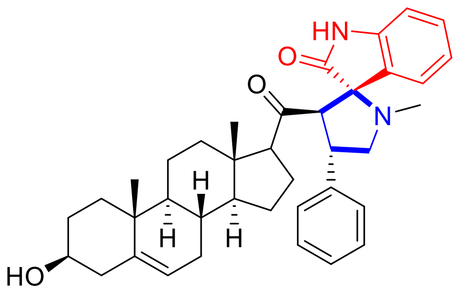

23a

IC_{50} = 23.3 ± 1.4, 18.8 ± 1.3, 5.1 ± 0.7, 8.3 ± 0.9 μM against T24, SMMC-7721, MCF7, and MGC-803, respectively

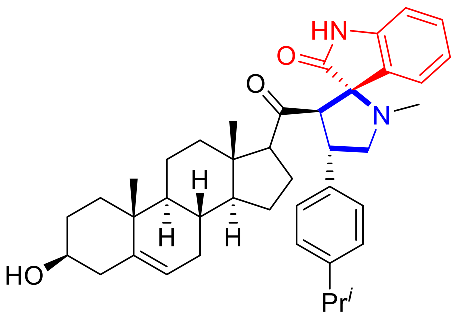

23b

IC_{50} = 80.9 ± 1.9, 35.2 ± 1.6, 21.8 ± 1.3, 15.6 ± 1.2 μM against T24, SMMC-7721, MCF7, and MGC-803, respectively

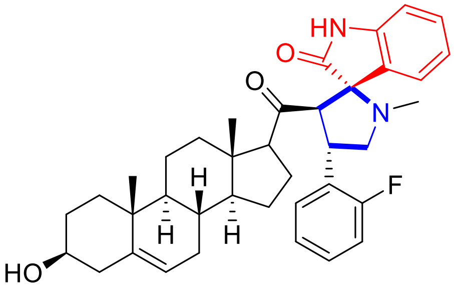

23c

IC_{50} = 4.1 ± 0.6, 43.1 ± 1.6, 17.7 ± 1.3, 16.1 ± 1.2 μM against T24, SMMC-7721, MCF7, and MGC-803, respectively

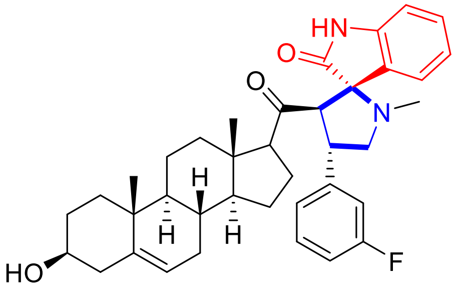

23d

IC_{50} = 6.1 ± 0.8, 6.7 ± 0.8, 4.6 ± 0.7, 10.2 ± 1.0 μM against T24, SMMC-7721, MCF7, and MGC-803, respectively

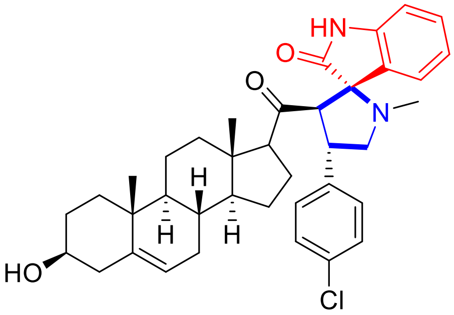

23e

IC_{50} = 15.4 ± 1.2, 18.5 ± 1.3, 4.0 ± 0.6, 6.2 ± 0.8 μM against T24, SMMC-7721, MCF7, and MGC-803, respectively

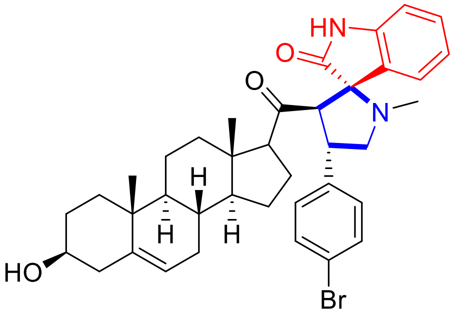

23f

IC_{50} = 14.4 ± 1.2, 9.3 ± 1.0, 3.9 ± 0.6, 11.0 ± 1.0 μM against T24, SMMC-7721, MCF7, and MGC-803, respectively

Fig. S2. Antiproliferation properties of stereoidal spirooxindoles **23** and 5-fluorouracil.

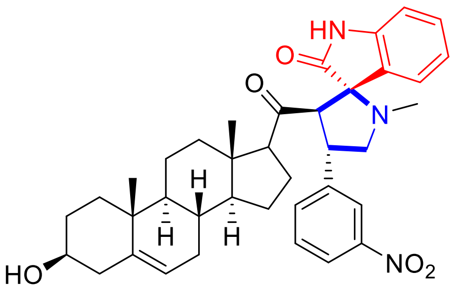

23g

IC_{50} = 8.8 ± 1.0, 44.0 ± 1.6, 10.2 ± 1.1, 13.5 ± 1.1 μM against T24, SMMC-7721, MCF7, and MGC-803, respectively

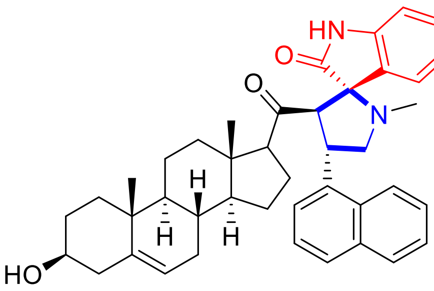

23h

IC_{50} = 44.1 ± 1.6, 12.2 ± 1.1, 9.9 ± 1.0, 14.0 ± 1.2 μM against T24, SMMC-7721, MCF7, and MGC-803, respectively

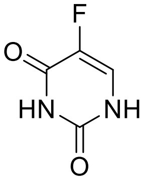

5- Fluorouracil

IC_{50} = 7.1 ± 0.9, 4.3 ± 0.6, 10.5 ± 1.6, 9.1 ± 1.0 μM against T24, SMMC-7721, MCF7, and MGC-803, respectively

Fig. S2 (continued)**.** Antiproliferation properties of stereoidal spirooxindoles **23** and 5-fluorouracil.

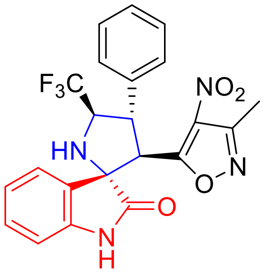

26a

K_i against MDM2 = 1.28 ± 0.23; IC_{50} = 0.38, 17.6 μM in case of without and with Fer-1

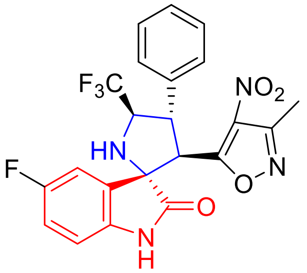

26b

K_i against MDM2 = 0.49 ± 0.08; IC_{50} = 0.21, 15.6 μM in case of without and with Fer-1

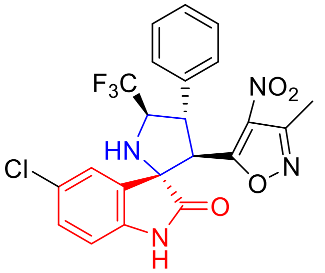

26c

K_i against MDM2 = 0.68 ± 0.13; IC_{50} = 0.37, 19.8 μM in case of without and with Fer-1

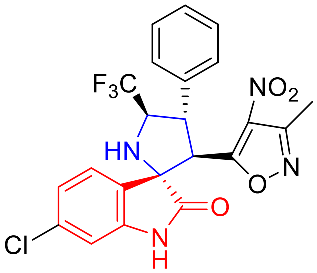

26d

K_i against MDM2 = 0.24 ± 0.06; IC_{50} = 0.12, 13.5 μM in case of without and with Fer-1

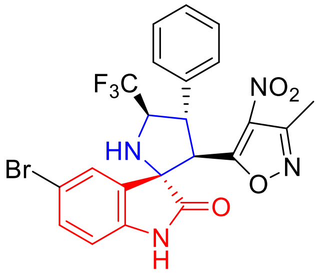

26e

K_i against MDM2 = 1.02 ± 0.23; IC_{50} = 0.55, 16.3 μM in case of without and with Fer-1

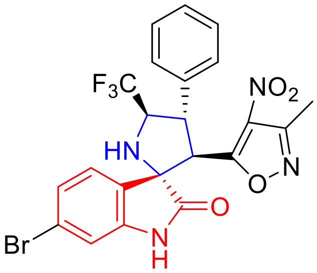

26f

K_i against MDM2 = 0.26 ± 0.05; IC_{50} = 0.17, 13.7 μM in case of without and with Fer-1

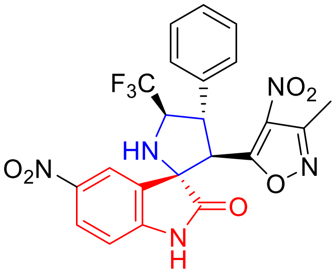

26g

K_i against MDM2 = 3.68 ± 0.57; IC_{50} = 4.75, >20 μM in case of without and with Fer-1

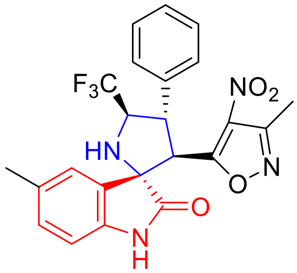

26h

K_i against MDM2 = 1.42 ± 0.21; IC_{50} = 4.91, 18.1 μM in case of without and with Fer-1

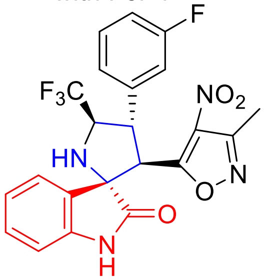

26i

K_i against MDM2 = 1.14 ± 0.20; IC_{50} = 0.87, 15.6 μM in case of without and with Fer-1

Fig. S3. K_i values (μM) and cytotoxicity against p53-wild MCF7 (breast) cancer cell line in absence and presence of Fer-1 of spirooxindoles bearing isoxazol-5-yl **26** and nutlin-3.

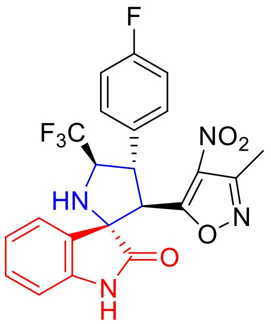

26j

K_i against MDM2 = 1.03 ± 0.14; IC_{50} = 0.55, 17.4 μM in case of without and with Fer-1

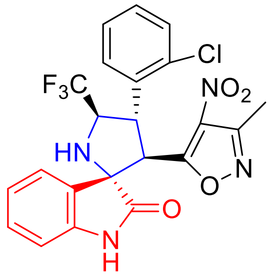

26k

K_i against MDM2 = >10; IC_{50} = 3.77, >20 μM in case of without and with Fer-1

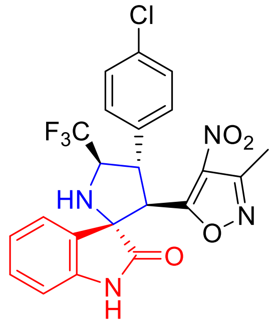

26l

K_i against MDM2 = 2.77 ± 0.31; IC_{50} = 1.14, >20 μM in case of without and with Fer-1

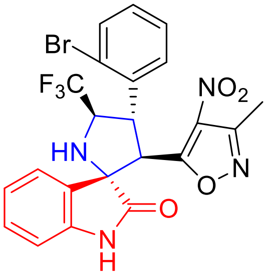

26m

K_i against MDM2 = >10; IC_{50} = 4.71, >20 μM in case of without and with Fer-1

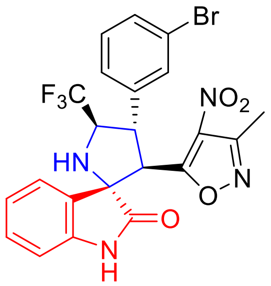

26n

K_i against MDM2 = 2.78 ± 0.37; IC_{50} = 2.31, >20 μM in case of without and with Fer-1

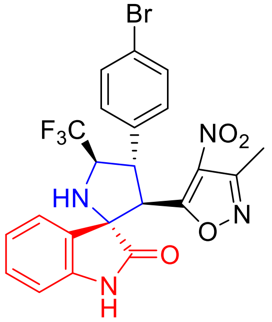

26o

K_i against MDM2 = 1.87 ± 0.20; IC_{50} = 3.01, 17.0 μM in case of without and with Fer-1

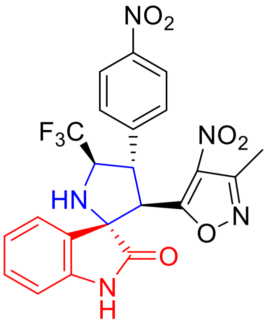

26p

K_i against MDM2 = 6.74 ± 0.96; IC_{50} = 5.36, >20 μM in case of without and with Fer-1

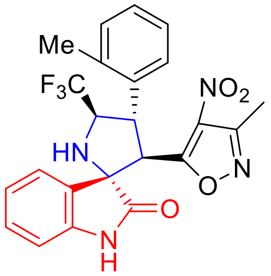

26q

K_i against MDM2 = >10; IC_{50} = 3.50, >20 μM in case of without and with Fer-1

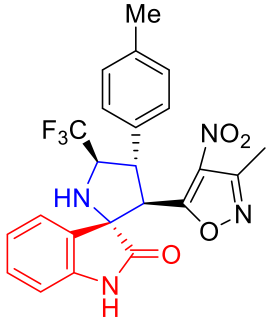

26r

K_i against MDM2 = 1.49 ± 0.36; IC_{50} = 2.81, 18.8 μM in case of without and with Fer-1

Fig. S3 (continued)**.** K_i values (μM) and cytotoxicity against p53-wild MCF7 (breast) cancer cell line in absence and presence of Fer-1 of spirooxindoles bearing isoxazol-5-yl **26** and nutlin-3.

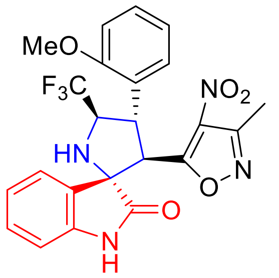

26s

K_i against MDM2 = >10; IC_{50} = 4.59, >20 μM in case of without and with Fer-1

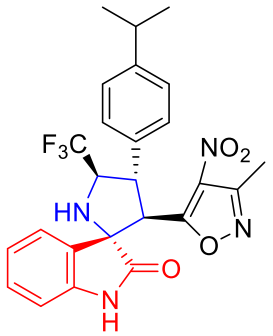

26t

K_i against MDM2 = 2.26 ± 0.37; IC_{50} = 2.83, >20 μM in case of without and with Fer-1

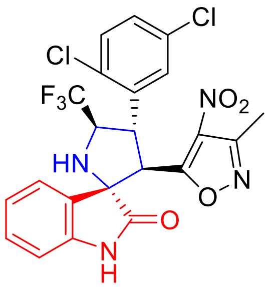

26u

K_i against MDM2 = >10; IC_{50} = 3.98, >20 μM in case of without and with Fer-1

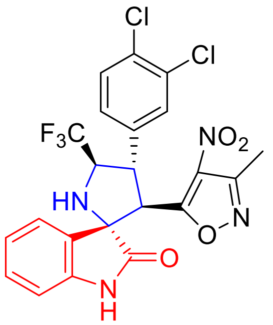

26v

K_i against MDM2 = 2.36 ± 0.17; IC_{50} = 3.22, >20 μM in case of without and with Fer-1

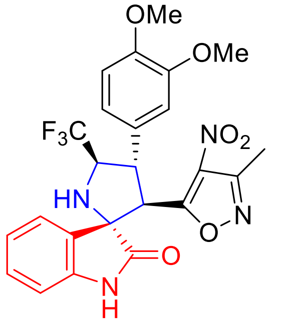

26w

K_i against MDM2 = 3.68 ± 0.29; IC_{50} = 4.13, >20 μM in case of without and with Fer-1

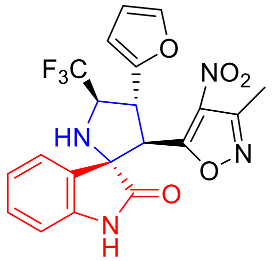

26x

K_i against MDM2 = 1.19 ± 0.21; IC_{50} = 1.46, 14.3 μM in case of without and with Fer-1

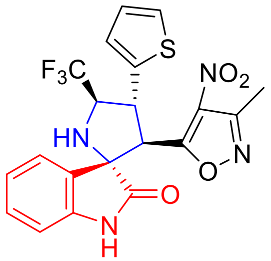

26y

K_i against MDM2 = 1.36 ± 0.24; IC_{50} = 1.53, 19.0 μM in case of without and with Fer-1

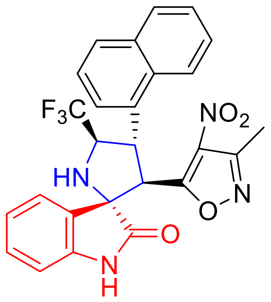

26z

K_i against MDM2 = 8.35 ± 1.04; IC_{50} = 7.17, >20 μM in case of without and with Fer-1

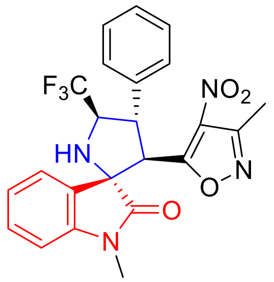

26aa

K_i against MDM2 = >10; IC_{50} = 3.52, >20 μM in case of without and with Fer-1

Fig. S3 (continued)**.** K_i values (μM) and cytotoxicity against p53-wild MCF7 (breast) cancer cell line in absence and presence of Fer-1 of spirooxindoles bearing isoxazol-5-yl **26** and nutlin-3.

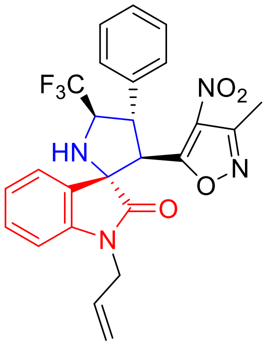

26ab

K_i against MDM2 = >10; IC_{50} = 3.42, >20 µM in case of without and with Fer-1

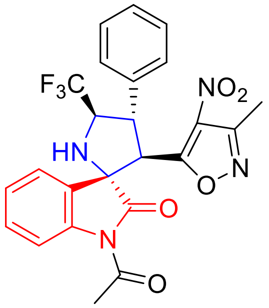

26ac

K_i against MDM2 = >10; IC_{50} = 3.23, >20 µM in case of without and with Fer-1

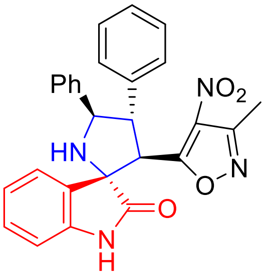

26ad

K_i against MDM2 = 6.33 ± 0.95; IC_{50} = 5.24, >20 µM in case of without and with Fer-1

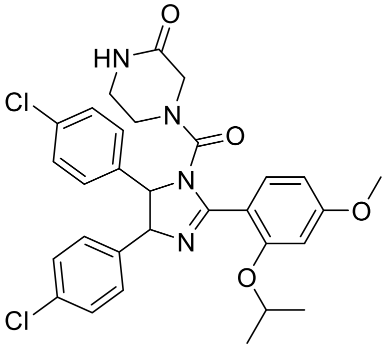

Nutlin-3

K_i against MDM2 = 0.28 ± 0.0; IC_{50} = 14.63, 15.72 µM in case of without and with Fer-1

Fig. S3 (continued)**.** K_i values (µM) and cytotoxicity against p53-wild MCF7 (breast) cancer cell line in absence and presence of Fer-1 of spirooxindoles bearing isoxazol-5-yl **26** and nutlin-3.

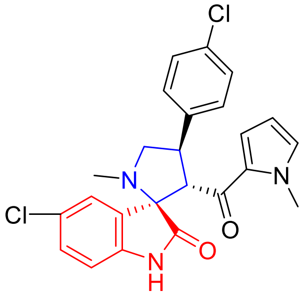

36a

IC_{50} = 6.4655 ± 0.1093, 4.4893 ± 0.3420, 4.1212 ± 0.0311 μM against MDA-MB-231, HepG-2, and Caco-2

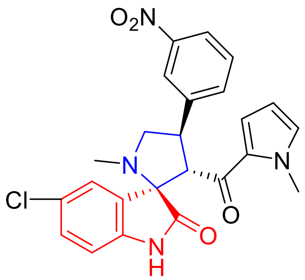

36b

IC_{50} = 1.0472 ± 0.0451, 9.4071 ± 0.3155, 9.7911 ± 0.666 μM against MDA-MB-231, HepG-2, and Caco-2

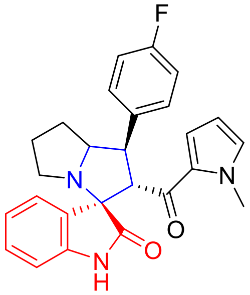

36c

IC_{50} = 19.200 ± 0.0741, 20.655 ± 0.3914, 18.361 ± 0.1642 μM against MDA-MB-231, HepG-2, and Caco-2; MDM2 binding K_D = 173 μM

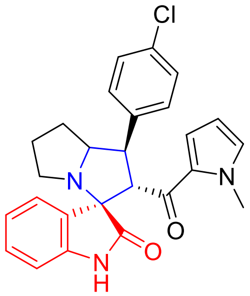

36d

IC_{50} = 0.7615 ± 0.0043, 5.8529 ± 0.2500, 5.6191 ± 0.1995 μM against MDA-MB-231, HepG-2, and Caco-2; MDM2 binding K_D = 102 μM

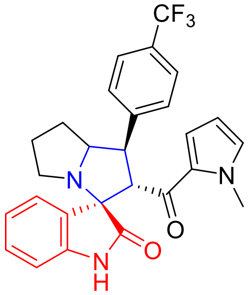

36e

IC_{50} = 0.8856 ± 0.0233, 8.6516 ± 0.1781, 8.7335 ± 0.4369 μM against MDA-MB-231, HepG-2, and Caco-2

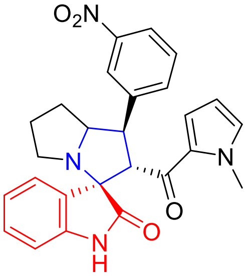

36f

IC_{50} = 0.9960 ± 0.0042, 7.9959 ± 0.2260, 8.6462 ± 0.4107 μM against MDA-MB-231, HepG-2, and Caco-2

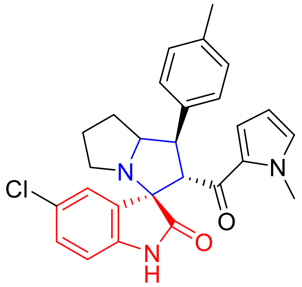

36g

IC_{50} = 0.5053 ± 0.0130, 3.6016 ± 0.0793, 4.2193 ± 0.2700 μM against MDA-MB-231, HepG-2, and Caco-2

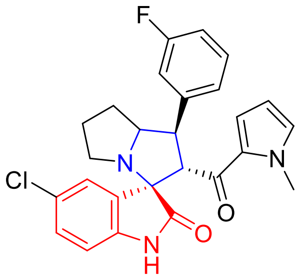

36h

IC_{50} = 0.6451 ± 0.0137, 8.9919 ± 0.0427, 4.2549 ± 0.0879 μM against MDA-MB-231, HepG-2, and Caco-2; no binding detected with with MDM2

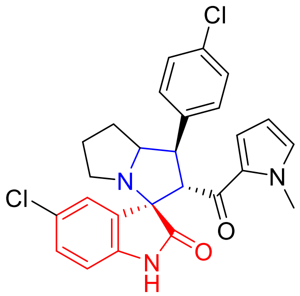

36i

IC_{50} = 0.2529 ± 0.0139, 1.3954 ± 0.0645, 0.9079 ± 0.0673 μM against MDA-MB-231, HepG-2, and Caco-2; MDM2 binding K_D = 1.32 μM

Fig. S4. Antiproliferation and MDM2 binding properties of spirooxindoles **36** and 5-fluorouracil.

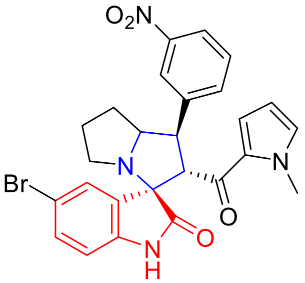

36j

IC_{50} = 0.3669 ± 0.0084, 0.2223 ± 0.0013, 0.2582 ± 0.0116 μM against MDA-MB-231, HepG-2, and Caco-2

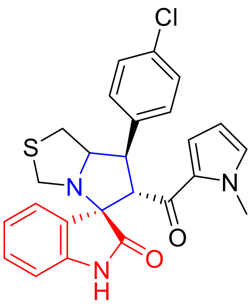

36k

IC_{50} = 0.0668 ± 0.0103, 0.1369 ± 0.0090, 0.5198 ± 0.0344 μM against MDA-MB-231, HepG-2, and Caco-2

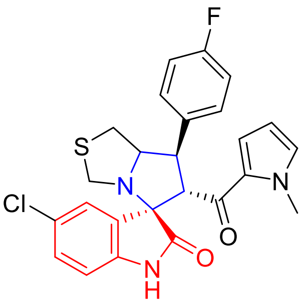

36l

IC_{50} = 10.873 ± 0.0041, 7.1605 ± 0.1010, 6.7823 ± 0.1334 μM against MDA-MB-231, HepG-2, and Caco-2; MDM2 binding K_D = 48.9 μM

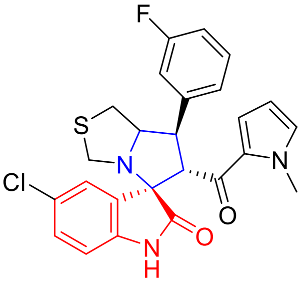

36m

IC_{50} = 0.1034 ± 0.0150, 0.6097 ± 0.0110, 0.5075 ± 0.0557 μM against MDA-MB-231, HepG-2, and Caco-2; MDM2 binding K_D = 7.75 μM

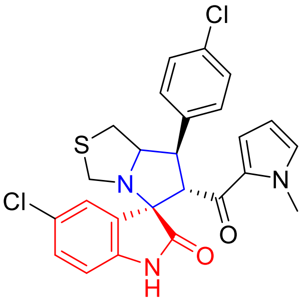

36n

IC_{50} = 0.0041 ± 0.0004, 0.0039 ± 0.0010, 0.0096 ± 0.0013 μM against MDA-MB-231, HepG-2, and Caco-2

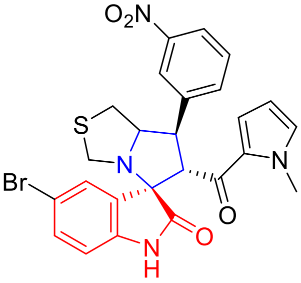

36o

IC_{50} = 0.0113 ± 0.0005, 0.0832 ± 0.0065, 0.0583 ± 0.0020 μM against MDA-MB-231, HepG-2, and Caco-2

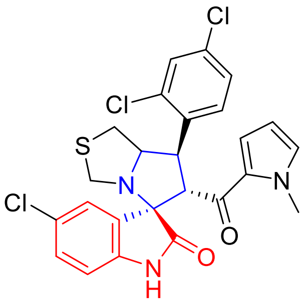

36p

IC_{50} = 0.0138 ± 0.0010, 3.3394 ± 0.1860, 0.0497 ± 0.0087 μM against MDA-MB-231, HepG-2, and Caco-2; MDM2 binding K_D = 2.79 μM

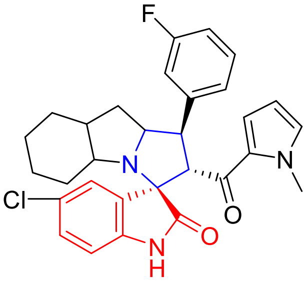

36q

IC_{50} = 0.0002 ± 0.0000, 0.0091 ± 0.0074, 0.0003 ± 0.0000 μM against MDA-MB-231, HepG-2, and Caco-2; MDM2 binding K_D = 1.72 μM

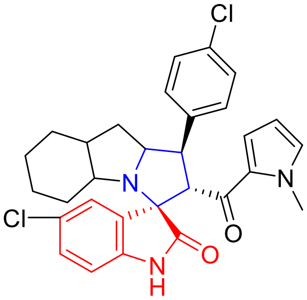

36r

IC_{50} = 0.0002 ± 0.0000, 0.0004 ± 0.0000, 0.0049 ± 0.0001 μM against MDA-MB-231, HepG-2, and Caco-2; MDM2 binding K_D = 0.612 μM

Fig. S4 (continued). Antiproliferation and MDM2 binding properties of spirooxindoles **36** and 5-fluorouracil.

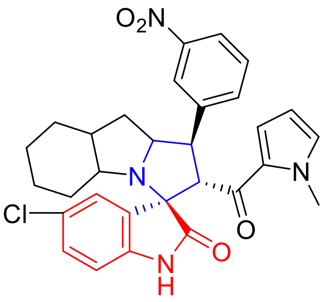

36s

IC_{50} = 0.1470 ± 0.0299, 3.7597 ± 0.0162, 0.0077 ± 0.0009 μM against MDA-MB-231, HepG-2, and Caco-2; MDM2 binding K_D = 1.13 μM

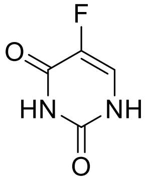

5- Fluorouracil

IC_{50} = 7.0500 ± 0.2040, 4.8290 ± 0.2960, 1.0480 ± 0.1560 μM against MDA-MB-231, HepG-2, and Caco-2

Fig. S4 (continued)**.** Antiproliferation and MDM2 binding properties of spirooxindoles **36** and 5-fluorouracil.

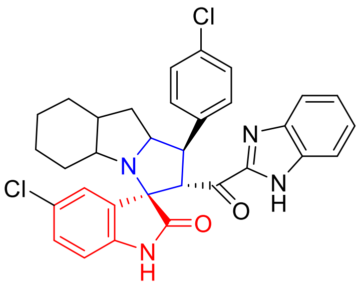

39a

IC_{50} = 4.763 ± 0.069, 4.574 ± 0.011 μM against MDA-MB-231, PC3, respectively; MST binding of MDM2, K_D = 2.38 μM

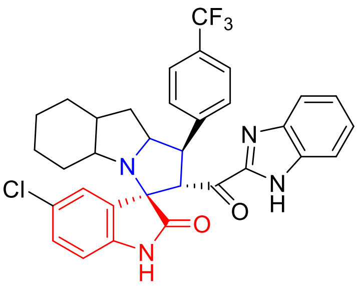

39b

IC_{50} = 4.284 ± 0.007, 4.404 ± 0.008 μM against MDA-MB-231, PC3, respectively

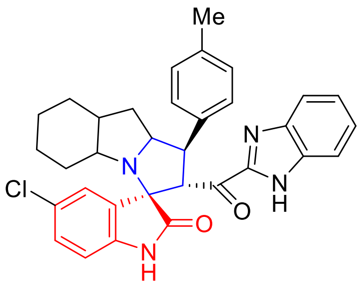

39c

IC_{50} = 4.221 ± 0.070, 4.504 ± 0.059 μM against MDA-MB-231, PC3, respectively

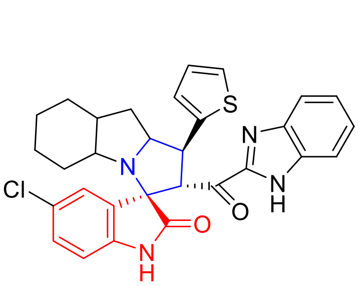

39d

IC_{50} = 3.797 ± 0.205, 4.314 ± 0.036 μM against MDA-MB-231, PC3, respectively

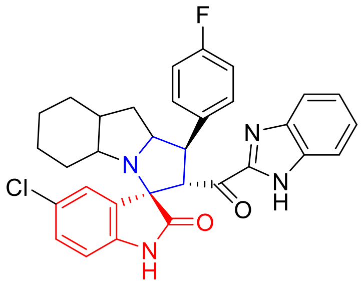

39e

IC_{50} = 4.143 ± 0.164, 4.252 ± 0.128 μM against MDA-MB-231, PC3, respectively

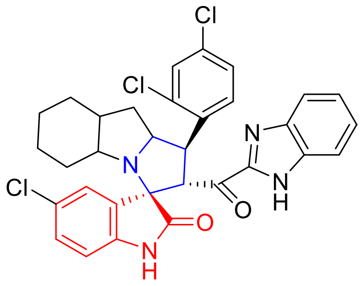

39f

IC_{50} = 4.274 ± 0.167, 4.388 ± 0.008 μM against MDA-MB-231, PC3, respectively

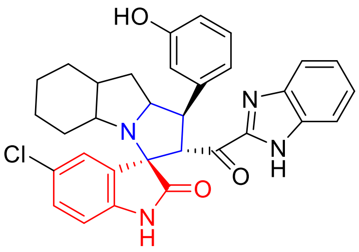

39g

IC_{50} = 4.329 ± 0.163, 4.294 ± 0.023 μM against MDA-MB-231, PC3, respectively; MST binding of MDM2, K_D = 38 μM

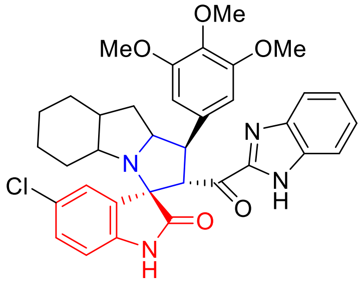

39h

IC_{50} = 4.261 ± 0.095, 4.241 ± 0.045 μM against MDA-MB-231, PC3, respectively; MST binding of MDM2, K_D = 10.6 μM

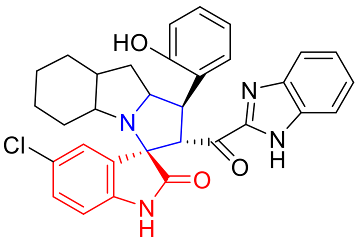

39i

IC_{50} = 4.371 ± 0.174, 4.495 ± 0.034 μM against MDA-MB-231, PC3, respectively

Fig. S5. Antiproliferation and MST inhibitory properties of MDM2 of spirooxindoles **39**.

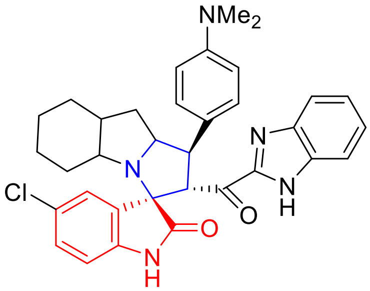

39j

IC_{50} = 4.261 ± 0.079, 4.420 ± 0.008 μM against MDA-MB-231, PC3, respectively

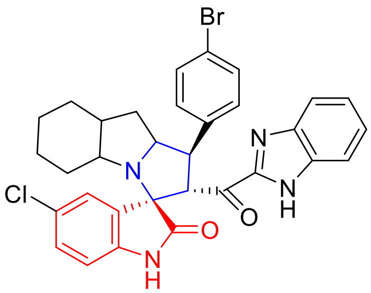

39k

IC_{50} = 4.479 ± 0.313, 4.539 ± 0.094 μM against MDA-MB-231, PC3, respectively; no MST binding of MDM2

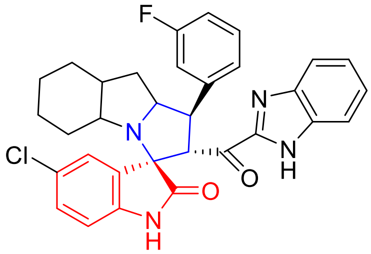

39l

IC_{50} = 4.156 ± 0.135, 4.360 ± 0.208 μM against MDA-MB-231, PC3, respectively

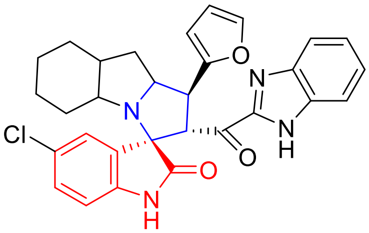

39m

IC_{50} = 6.039 ± 0.111, 5.098 ± 0.119 μM against MDA-MB-231, PC3, respectively

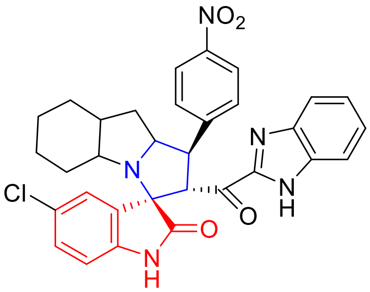

39n

IC_{50} = 6.879 ± 0.308, 7.567 ± 0.709 μM against MDA-MB-231, PC3, respectively

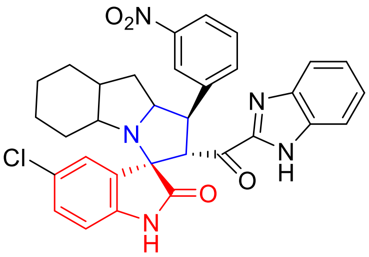

39o

IC_{50} = 4.138 ± 0.186, 4.275 ± 0.005 μM against MDA-MB-231, PC3, respectively

Fig. S5 (continued)**.** Antiproliferation and MST inhibitory properties of MDM2 of spirooxindoles **39**.

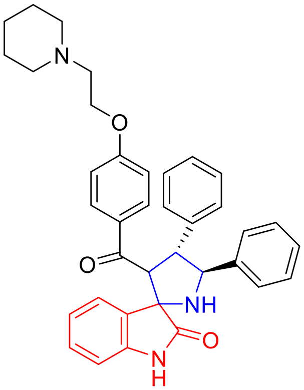

47a

IC_{50} = 28.2, 13.9, 30.8, 32.4 µM against MDA-MB-231, MCF7, HEK-293, and VERO, respectively

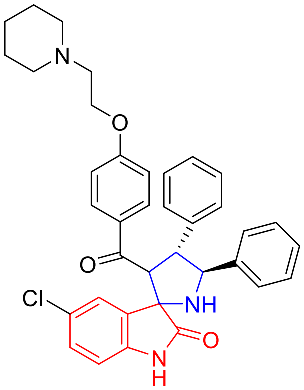

47b

IC_{50} = 3.7, 6.5, >50, >50 µM against MDA-MB-231, MCF7, HEK-293, and VERO, respectively

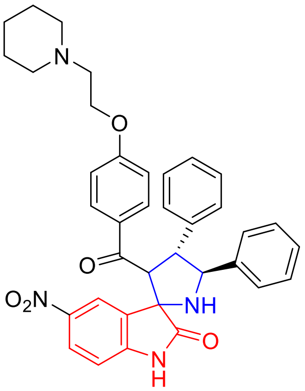

47c

IC_{50} = 13.5, 15.2, 18.8, 22.4 µM against MDA-MB-231, MCF7, HEK-293, and VERO, respectively

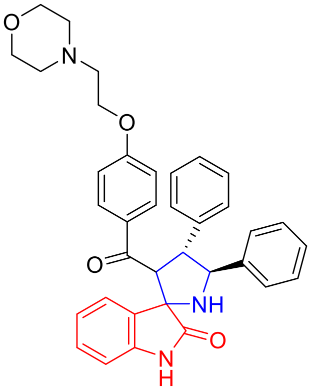

47d

IC_{50} = >50, >50, >50, >50 µM against MDA-MB-231, MCF7, HEK-293, and VERO, respectively

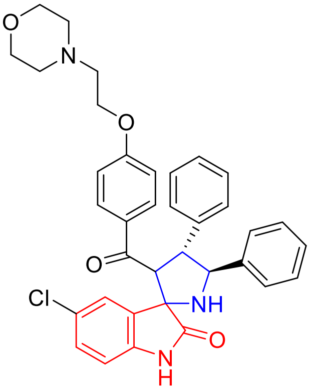

47e

IC_{50} = 19.2, 18, 25.4, 30.8 µM against MDA-MB-231, MCF7, HEK-293, and VERO, respectively

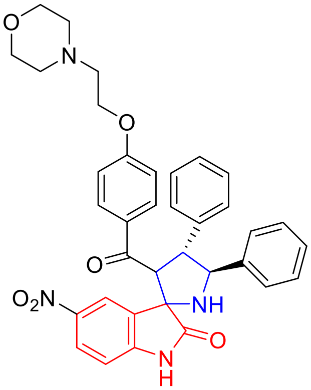

47f

IC_{50} = >50, 24.7, 45.4, >50 µM against MDA-MB-231, MCF7, HEK-293, and VERO, respectively

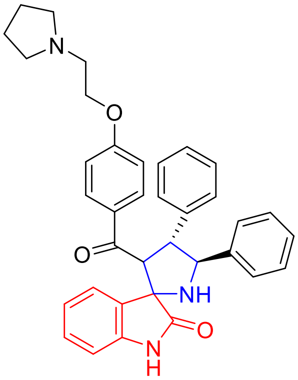

47g

IC_{50} = 16.8, 23, 25.3, 19.4 µM against MDA-MB-231, MCF7, HEK-293, and VERO, respectively

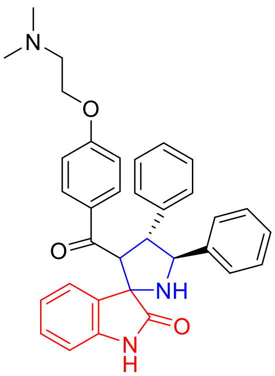

47h

IC_{50} = 9.2, 9.6, >50, >50 µM against MDA-MB-231, MCF7, HEK-293, and VERO, respectively

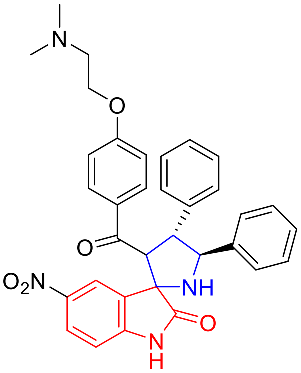

47i

IC_{50} = 17.8, 17.2, 24.2, 28.8 µM against MDA-MB-231, MCF7, HEK-293, and VERO, respectively

Fig. S6. Antiproliferation properties of spirooxindoles **47** and nutlin-3.

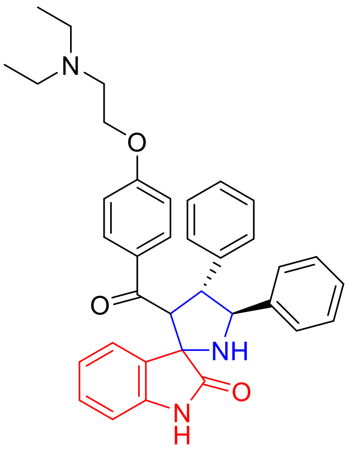

47j

IC_{50} = 14.5, 13.4, 23.2, 20.6 μM against MDA-MB-231, MCF7, HEK-293, and VERO, respectively

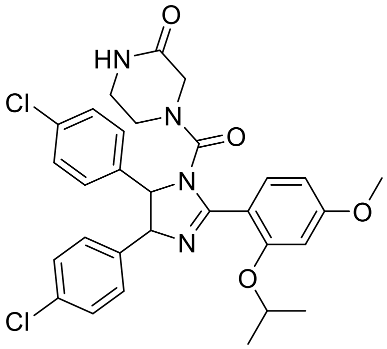

Nutlin-3

IC_{50} = 23.5, 11.6, >50, >50 μM against MDA-MB-231, MCF7, HEK-293, and VERO, respectively

Fig. S6 (continued)**.** Antiproliferation properties of spirooxindoles **47** and nutlin-3.

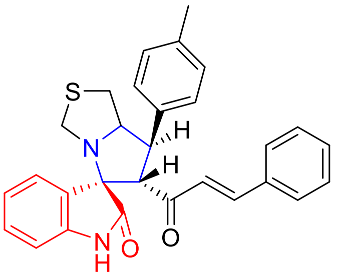

49a

IC_{50} = 9 ± 1, 3.85 ± 0.3, >50, 8 ± 0.5 μM against HCT116, HepG2, PC-3, and VERO-B, respectively

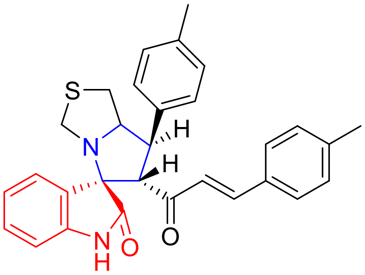

49b

IC_{50} = 8 ± 1.2, 3.57 ± 0.3, >50, 11.8 ± 1 μM against HCT116, HepG2, PC-3, and VERO-B, respectively

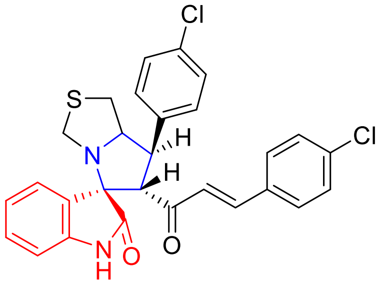

49c

IC_{50} = 3 ± 0.5, 2.0 ± 0.5, 2.7 ± 0.4, 7 ± 0.45 μM against HCT116, HepG2, PC-3, and VERO-B, respectively

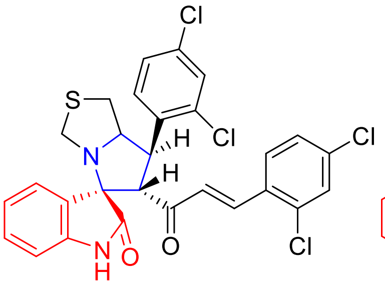

49d

IC_{50} = 2 ± 0.6, 0.85 ± 0.2, 1.8 ± 0.3, 5 ± 0.245 μM against HCT116, HepG2, PC-3, and VERO-B, respectively

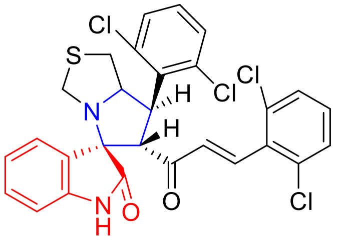

49e

IC_{50} = 3 ± 0.3, 1.0 ± 0.3, >50, 3 ± 0.5 μM against HCT116, HepG2, PC-3, and VERO-B, respectively

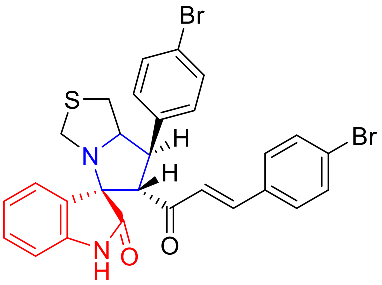

49f

IC_{50} = 3 ± 0.5, 0.8 ± 0.1, >50, 3 ± 0.32 μM against HCT116, HepG2, PC-3, and VERO-B, respectively

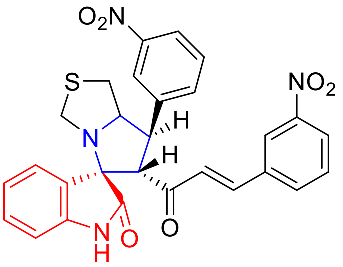

49g

IC_{50} = 2.5 ± 0.2, >50, >50, 2.5 ± 0.2 μM against HCT116, HepG2, PC-3, and VERO-B, respectively

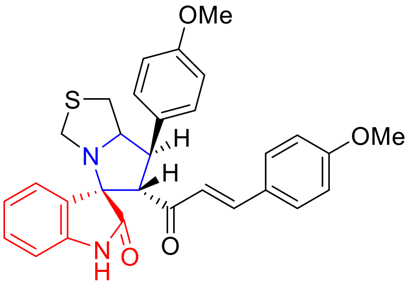

49h

IC_{50} = 7 ± 0.9, 2.25 ± 0.2, >50, 7.5 ± 0.6 μM against HCT116, HepG2, PC-3, and VERO-B, respectively

Fig. S7. Antiproliferation properties of spirooxindoles **49** and cisplatin.

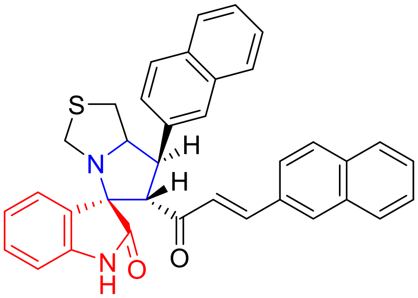

49i

IC_{50} = 8 ± 0.3, 2.4 ± 1.0, 2.9 ± 0.2, 7 ± 0.28 μM against HCT116, HepG2, PC-3, and VERO-B, respectively

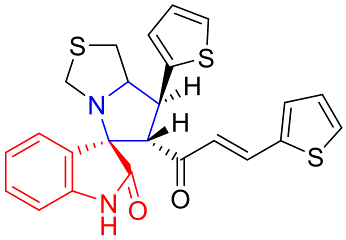

49j

IC_{50} = 14.5 ± 1.5, >50, >50, 18 ± 2 μM against HCT116, HepG2, PC-3, and VERO-B, respectively

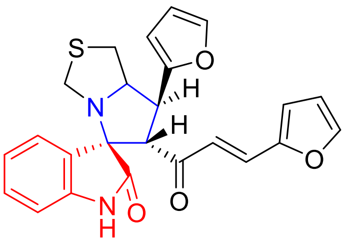

49k

IC_{50} = 19 ± 2, >50, >50, 25 ± 2.5 μM against HCT116, HepG2, PC-3, and VERO-B, respectively

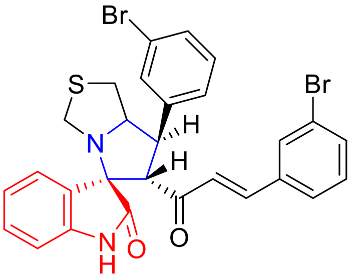

49l

IC_{50} = 1.57 ± 0.3, 0.9 ± 0.1, 1.5 ± 0.5, 3 ± 0.56 μM against HCT116, HepG2, PC-3, and VERO-B, respectively

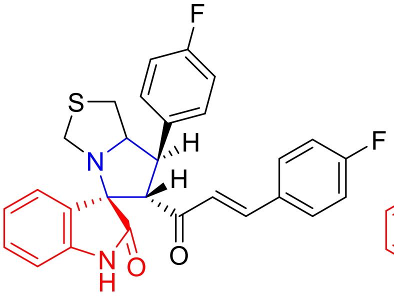

49m

IC_{50} = 5 ± 0.3, 2.4 ± 0.4, 2.5 ± 0.2, 6 ± 0.7 μM against HCT116, HepG2, PC-3, and VERO-B, respectively

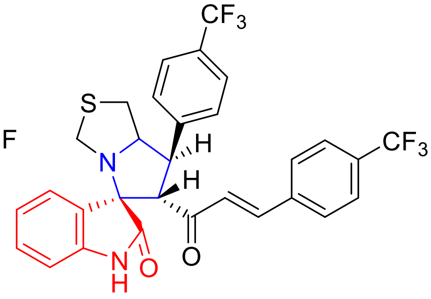

49n

IC_{50} = 2.9 ± 0.4, 0.9 ± 0.2, 1.0 ± 0.2, 2.5 ± 0.12 μM against HCT116, HepG2, PC-3, and VERO-B, respectively

Fig. S7 (continued)**.** Antiproliferation properties of spirooxindoles **49** and cisplatin.

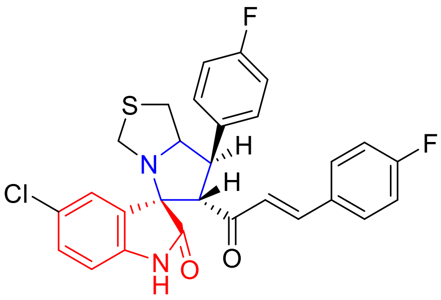

49o

IC_{50} = 7 ± 0.6, 2 ± 0.4, 2.5 ± 0.5, 8 ± 0.78 μM against HCT116, HepG2, PC-3, and VERO-B, respectively

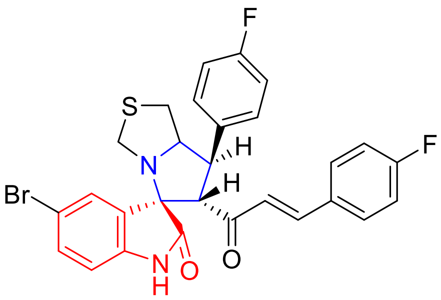

49p

IC_{50} = 3.5 ± 0.3, 0.8 ± 0.1, 1.5 ± 0.1, 3 ± 0.35 μM against HCT116, HepG2, PC-3, and VERO-B, respectively

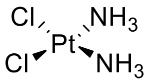

Cisplatin

IC_{50} = 12.6 ± 2, 5.5 ± 1, 5.0 ± 0.5, 5 ± 0.2 μM against HCT116, HepG2, PC-3, and VERO-B, respectively

Fig. S7 (continued)**.** Antiproliferation properties of spirooxindoles **49** and cisplatin.

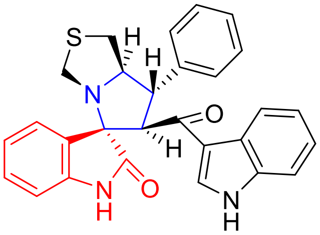

51a

IC_{50} = 30 ± 3, 30 ± 2.4, 30 ± 3.4, 68 μM against HCT116, HepG2, PC3 and VERO-B cell lines, respectively

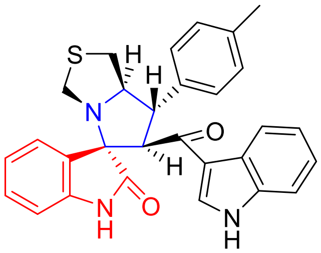

51b

IC_{50} = 9 ± 1.78, 10 ± 2, 15.3 ± 2.3, 28 μM against HCT116, HepG2, PC3 and VERO-B cell lines, respectively

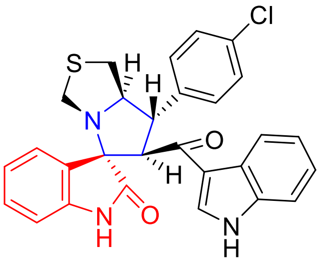

51c

IC_{50} = 11.5 ± 2, 15 ± 1.1, 18.4 ± 1.5, 40 μM against HCT116, HepG2, PC3 and VERO-B cell lines, respectively

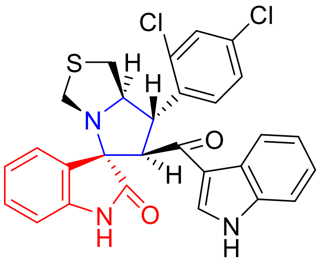

51d

IC_{50} = 9 ± 1.5, 7 ± 1, 6 ± 0.3, 26 μM against HCT116, HepG2, PC3 and VERO-B cell lines, respectively

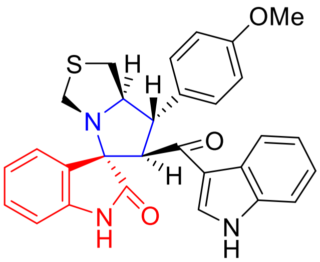

51e

IC_{50} = 26 ± 2, 30 ± 3, 24.6 ± 3, 50 μM against HCT116, HepG2, PC3 and VERO-B cell lines, respectively

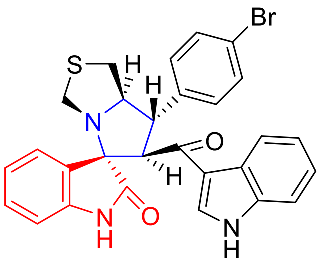

51f

IC_{50} = 11.3 ± 1, 8 ± 1.56, 8 ± 0.5, 22 μM against HCT116, HepG2, PC3 and VERO-B cell lines, respectively

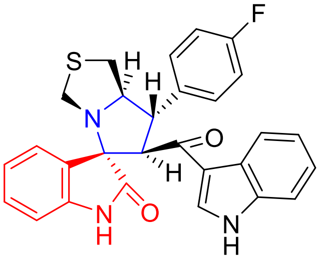

51g

IC_{50} = 13 ± 1.2, 12 ± 1, 16.8 ± 2, 30 μM against HCT116, HepG2, PC3 and VERO-B cell lines, respectively

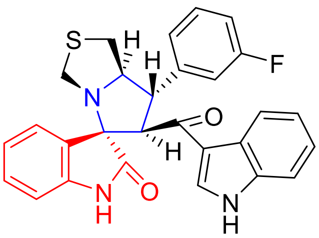

51h

IC_{50} = 9 ± 0.98, 8 ± 2.1, 8 ± 0.45, 22 μM against HCT116, HepG2, PC3 and VERO-B cell lines, respectively

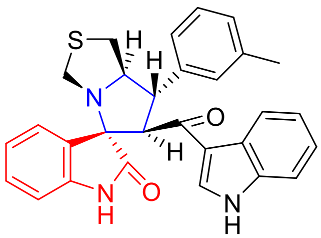

51i

IC_{50} = 15.3 ± 2, 12 ± 2, 17.7 ± 3, 32 μM against HCT116, HepG2, PC3 and VERO-B cell lines, respectively

Fig. S8. Antiproliferation properties of spirooxindoles linked to 3-acylindole **51** and cisplatin.

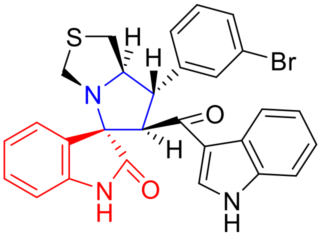

51j

IC_{50} = 9 ± 1, 7 ± 1.5, 7 ± 0.15, 20 μM against HCT116, HepG2, PC3 and VERO-B cell lines, respectively

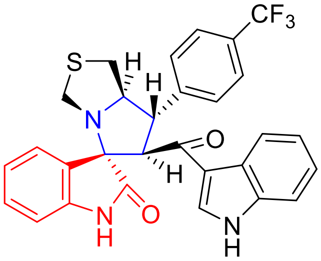

51k

IC_{50} = 7 ± 0.27, 5.5 ± 0.2, 6 ± 0.23, 26 μM against HCT116, HepG2, PC3 and VERO-B cell lines, respectively

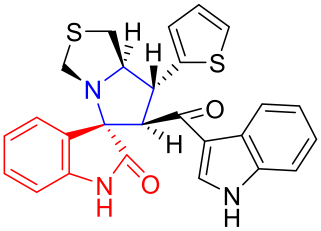

51l

IC_{50} = 90 ± 8, 35 ± 4, 25 ± 2.9, 40 μM against HCT116, HepG2, PC3 and VERO-B cell lines, respectively

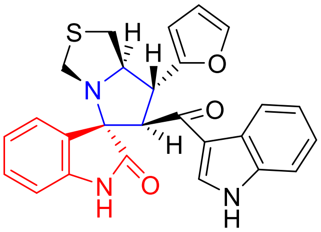

51m

IC_{50} = 40 ± 5, 30 ± 2, 18.5 ± 1.86, 33 μM against HCT116, HepG2, PC3 and VERO-B cell lines, respectively

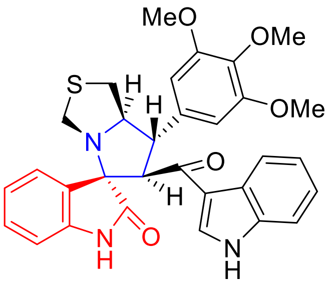

51n

IC_{50} = >40 ± 3, 35 ± 3, 18.5 ± 2, 42 μM against HCT116, HepG2, PC3 and VERO-B cell lines, respectively

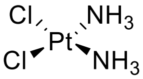

Cisplatin

IC_{50} = 12.6 ± 0.5, 5.5 ± 0.3, 5 ± 0.56, 5 μM against HCT116, HepG2, PC3 and VERO-B cell lines, respectively

Fig. S8 (continued)**.** Antiproliferation properties of spirooxindoles linked to 3-acylindole **51** and cisplatin.

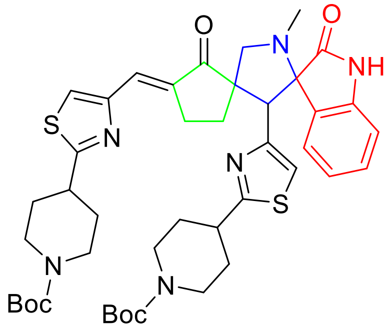

65a

IC_{50} = 153.1, 168.4 μM against MCF7 and HeLa cell lines, respectively

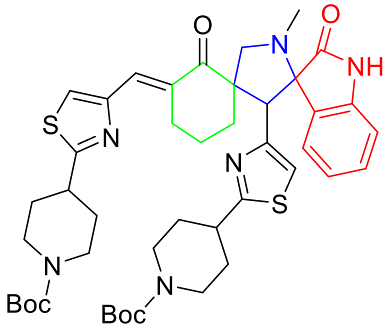

65b

IC_{50} = 93.3, 99.2 μM against MCF7 and HeLa cell lines, respectively

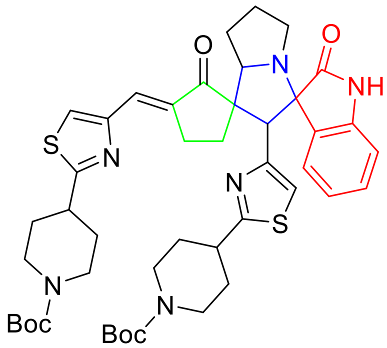

66a

IC_{50} = 68.7, 71.4 μM against MCF7 and HeLa cell lines, respectively

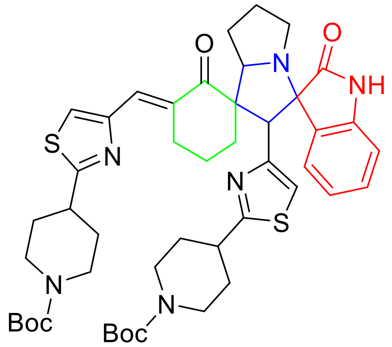

66b

IC_{50} = 76.8, 82.6 μM against MCF7 and HeLa cell lines, respectively

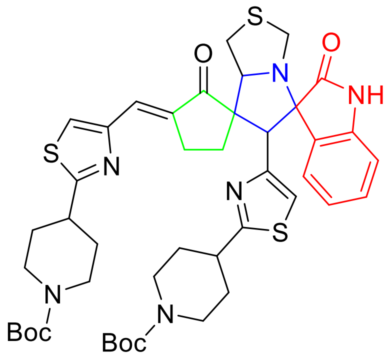

67a

IC_{50} = 87.9, 92.3 μM against MCF7 and HeLa cell lines, respectively

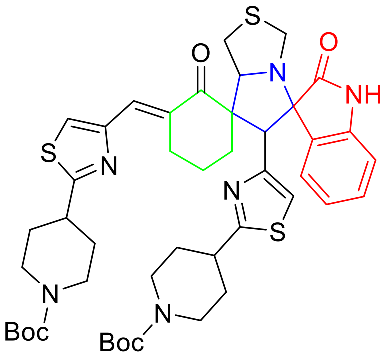

67b

IC_{50} = 75.9, 85.7 μM against MCF7 and HeLa cell lines, respectively

Fig. S9. Antiproliferation properties of dispirooxindole-pyrrolidines **65**–**68** and doxorubicin.

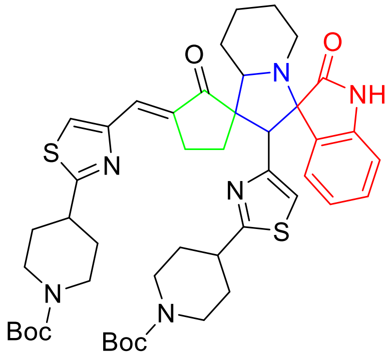

68a

IC_{50} = 63.4, 76.3 μM
against MCF7 and HeLa
cell lines, respectively

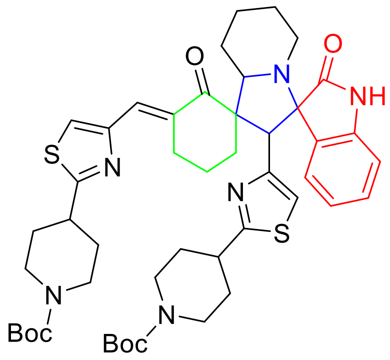

68b

IC_{50} = 91.2, 105.4 μM
against MCF7 and HeLa
cell lines, respectively

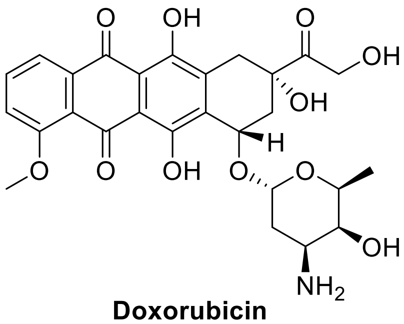

Doxorubicin

IC_{50} = 59.4, 65.3 μM
against MCF7 and HeLa
cell lines, respectively

Fig. S9. (continued)**.** Antiproliferation properties of dispirooxindole-pyrrolidines **65**–**68** and doxorubicin.

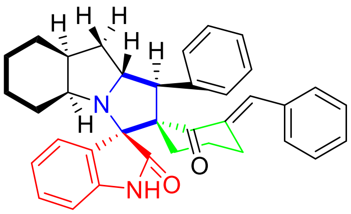

70a

IC_{50} = 24.1 ± 1.1, 7.1 ± 0.2, 25.04 ± 0.57, 19.50 ± 0.56 μM against PC3, HeLa , MCF7, and MDA-MB-231 cell lines, respectively

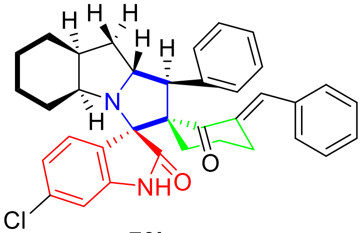

70b

IC_{50} = 3.7 ± 1.0, 27.72 ± 0.59, 24.08 ± 0.02 μM against PC3, MCF7, and MDA-MB-231 cell lines, respectively

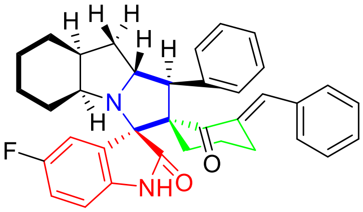

70c

IC_{50} = 17.9 ± 0.2, 27.82 ± 1.02, 20.62 ± 2.16 μM against PC3, MCF7, and MDA-MB-231 cell lines, respectively

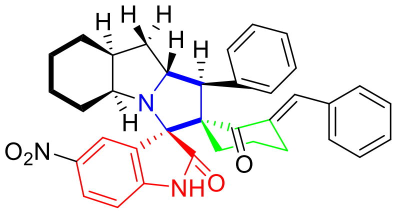

70d

IC_{50} = 29.8 ± 0.1 μM against PC3 cell line

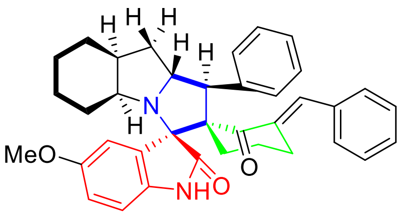

70e

IC_{50} = 19.6 ± 1.2, 26.5 ± 0.04 μM against PC3 and HeLa cell lines, respectively

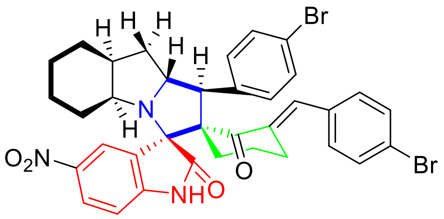

70f

IC_{50} = 14.3 ± 1.0 μM against PC3 cell line

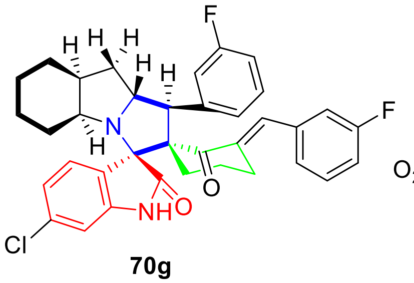

70g

IC_{50} = 14.43 ± 0.09 μM against MDA-MB-231 cell line

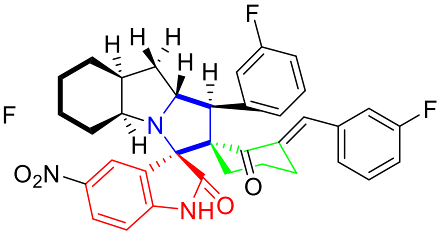

70h

IC_{50} = 7.63 ± 0.08 μM against MDA-MB-231 cell line

Fig. S10. Antiproliferation properties of dispirooxindole-pyrrolidines **70** and doxorubicin.

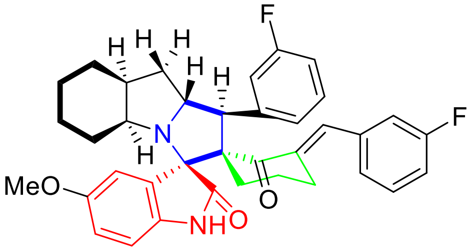

70i

IC_{50} = 11.9 ± 0.04, 10.49 ± 0.71 μM against HeLa and MDA-MB-231 cell lines, respectively

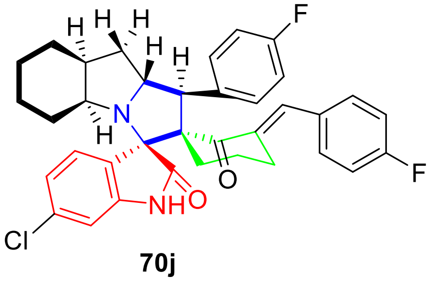

70j

IC_{50} = 7.2 ± 0.5, 14.45 ± 0.08 μM against HeLa , and MDA-MB-231 cell lines, respectively

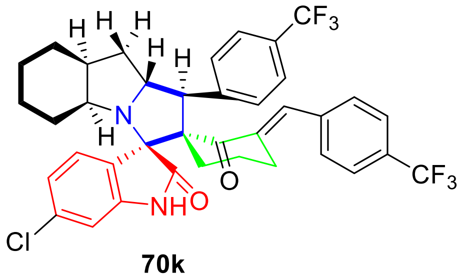

70k

IC_{50} = 24.6 ± 0.4 μM against HeLa cell line

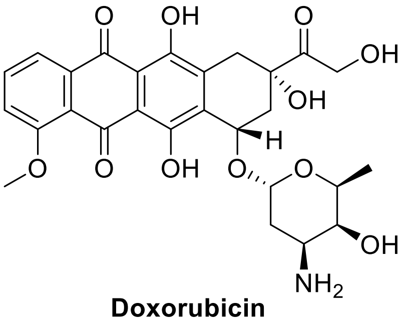

Doxorubicin

IC_{50} = 1.9 ± 0.4, 0.9 ± 0.14, 0.79 ± 0.05, 0.32 ± 0.002 μM against PC3, HeLa , MCF7, and MDA-MB-231 cell lines, respectively

Fig. S10 (continued)**.** Antiproliferation properties of dispirooxindole-pyrrolidines **70** and doxorubicin.

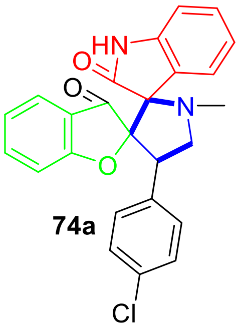

74a

IC_{50} = 21 ± 5.5, >100, 8.9 ± 0.4, 93 ± 9.7, 36 ± 4.6, 15 ± 2.6 μM against A549, HBL-100, HeLa, SW1573, T-47D, and WiDr cell lines, respectively

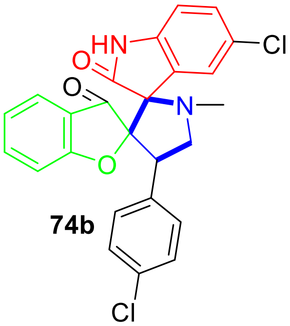

74b

IC_{50} = 4.4 ± 2.4, 24 ± 8.2, 5.0 ± 1.5, 17 ± 1.2, 19 ± 2.9, 21 ± 4.9 μM against A549, HBL-100, HeLa, SW1573, T-47D, and WiDr cell lines, respectively

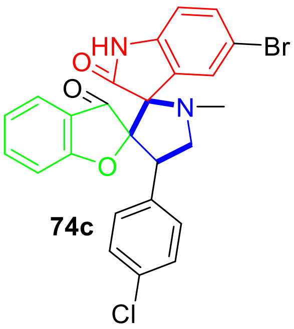

74c

IC_{50} = 20 ± 2.7, 42 ± 10, 7.7 ± 2.4, 20 ± 1.3, 18 ± 1.5, 23 ± 5.4 μM against A549, HBL-100, HeLa, SW1573, T-47D, and WiDr cell lines, respectively

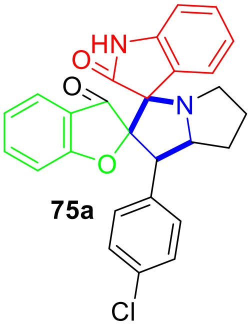

75a

IC_{50} = 3.4 ± 0.8, 4.5 ± 1.2, 2.6 ± 0.5, 18 ± 2.8, 3.2 ± 0.3, 3.6 ± 0.4 μM against A549, HBL-100, HeLa, SW1573, T-47D, and WiDr cell lines, respectively

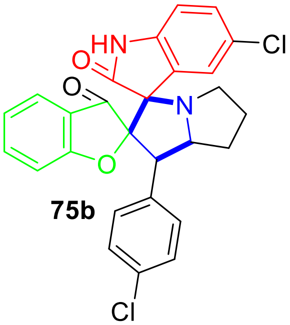

75b

IC_{50} = 4.5 ± 1.1, 4.3 ± 1.1, 2.8 ± 0.1, 7.0 ± 0.2, 3.4 ± 0.3, 3.2 ± 0.5 μM against A549, HBL-100, HeLa, SW1573, T-47D, and WiDr cell lines, respectively

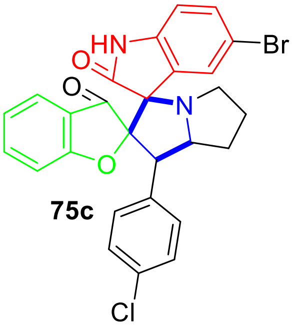

75c

IC_{50} = 3.6 ± 1.0, 4.1 ± 1.1, 2.7 ± 0.4, 4.6 ± 1.4, 13 ± 1.7, 11 ± 2.2 μM against A549, HBL-100, HeLa, SW1573, T-47D, and WiDr cell lines, respectively

Fig. S11. Antiproliferation properties of dispirooxindole-pyrrolidines collaborating benzofuranyl heterocycle **74**–**79** and standard references (cisplatin, etoposide, and camptothecin).

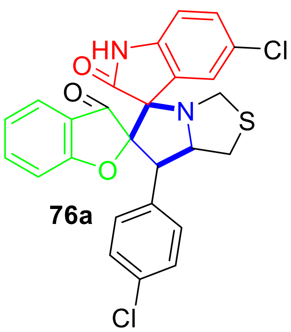

76a

IC_{50} = 4.1 ± 1.0, 7.4 ± 1.8, 3.1 ± 0.7, 6.4 ± 0.7, 4.0 ± 0.2, 5.1 ± 1.1 μM against A549, HBL-100, HeLa, SW1573, T-47D, and WiDr cell lines, respectively

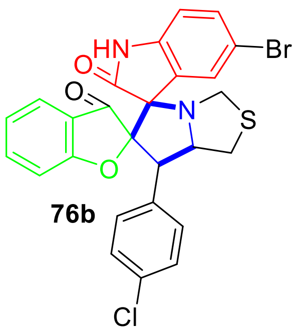

76b

IC_{50} = 4.2 ± 1.3, 7.6 ± 0.6, 3.1 ± 0.6, 9.3 ± 2.0, 5.5 ± 0.3, 6.2 ± 1.0 μM against A549, HBL-100, HeLa, SW1573, T-47D, and WiDr cell lines, respectively

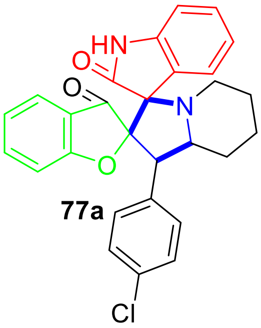

77a

IC_{50} = 4.0 ± 0.4, 5.0 ± 0.3, 2.5 ± 0.3, 8.1 ± 0.03, 4.2 ± 0.4, 4.6 ± 0.7 μM against A549, HBL-100, HeLa, SW1573, T-47D, and WiDr cell lines, respectively

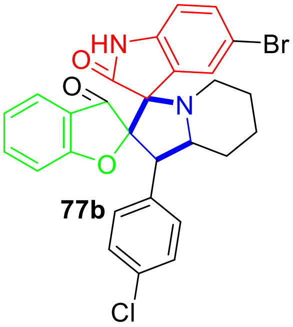

77b

IC_{50} = 4.9 ± 0.7, 14 ± 3.2, 3.3 ± 0.7, 11 ± 1.9, 8.5 ± 0.8, 9.6 ± 1.9 μM against A549, HBL-100, HeLa, SW1573, T-47D, and WiDr cell lines, respectively

Fig. S11 (continued)**.** Antiproliferation properties of dispirooxindole-pyrrolidines collaborating benzofuranyl heterocycle **74–79** and standard references (cisplatin, etoposide, and camptothecin).

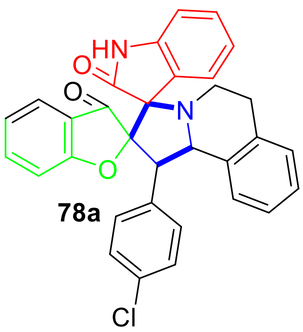

78a

IC_{50} = 20 ± 4.7, >100,
7.9 ± 1.3, >100,
93 ± 12, 89 ± 15 μM
against A549, HBL-100,
HeLa, SW1573, T-47D,
and WiDr cell lines,
respectively

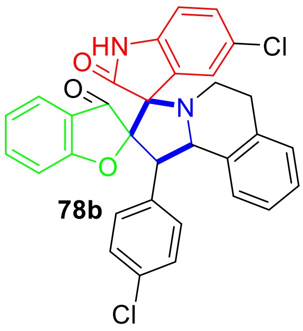

78b

IC_{50} = 23 ± 10, >100,
5.4 ± 2.9, >100,
75 ± 3.6, 46 ± 13 μM
against A549, HBL-100,
HeLa, SW1573, T-47D,
and WiDr cell lines,
respectively

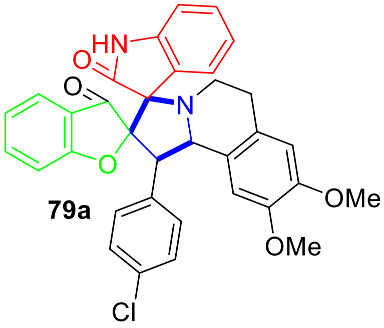

79a

IC_{50} = 27 ± 6.6, 94 ± 10,
9.0 ± 3.1, >100,
94 ± 11, >10 μM
against A549, HBL-100,
HeLa, SW1573, T-47D,
and WiDr cell lines,
respectively

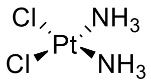

Cisplatin

IC_{50} = 1.9 ± 0.2, 2.0 ± 0.3,
3.0 ± 0.4, 17 ± 3.3,
26 ± 5.3 μM
against HBL-100,
HeLa, SW1573, T-47D,
and WiDr cell lines,
respectively

Fig. S11 (continued)**.** Antiproliferation properties of dispirooxindole-pyrrolidines collaborating benzofuranyl heterocycle **74**–**79** and standard references (cisplatin, etoposide, and camptothecin).

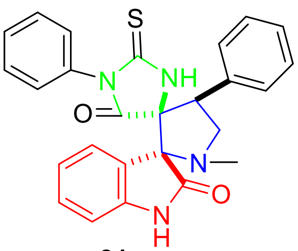

81a

IC_{50} = >100, >100, >100, >100 μM against LNCaP ($p53^{+}$), PC3 ($p53^{-}$), HCT^{wt} ($p53^{+}$), and $HCT^{-/-}$ ($p53^{-}$), respectively

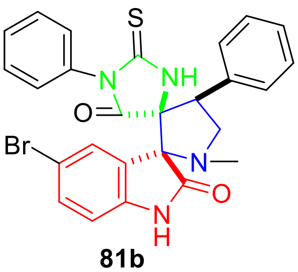

81b

IC_{50} = 2.2 ± 0.8, 4.6 ± 2.5, 10.5 ± 2.3, 12.5 ± 3.2 μM against LNCaP ($p53^{+}$), PC3 ($p53^{-}$), HCT^{wt} ($p53^{+}$), and $HCT^{-/-}$ ($p53^{-}$), respectively

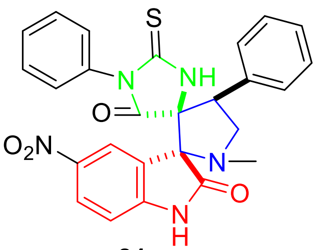

81c

IC50 = >100, >100, >100, >100 μM against LNCaP ($p53^{+}$), PC3 ($p53^{-}$), HCTwt ($p53^{+}$), and $HCT^{-}/^{-}$ ($p53^{-}$), respectively

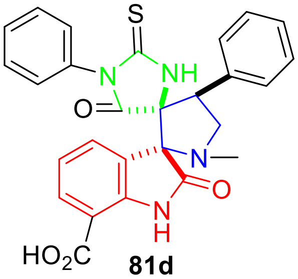

81d

IC50 = >100, >100, >100, >100 μM against LNCaP ($p53^{+}$), PC3 ($p53^{-}$), HCTwt ($p53^{+}$), and $HCT^{-}/^{-}$ ($p53^{-}$), respectively

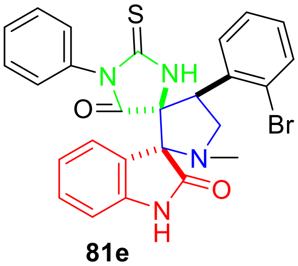

81e

IC50 = >100, >100, >100, >100 μM against LNCaP ($p53^{+}$), PC3 ($p53^{-}$), HCTwt ($p53^{+}$), and $HCT^{-}/^{-}$ ($p53^{-}$), respectively

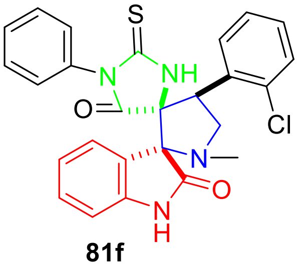

81f

IC50 = >100, >100, 21.7 ± 5.8, 23.1 ± 6.6 μM against LNCaP ($p53^{+}$), PC3 ($p53^{-}$), HCTwt ($p53^{+}$), and $HCT^{-}/^{-}$ ($p53^{-}$), respectively

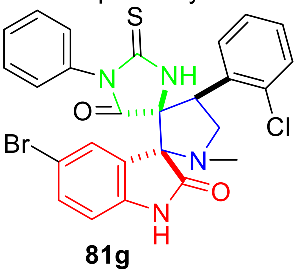

81g

IC50 = >100, >100 μM against LNCaP ($p53^{+}$), and PC3 ($p53^{-}$), respectively

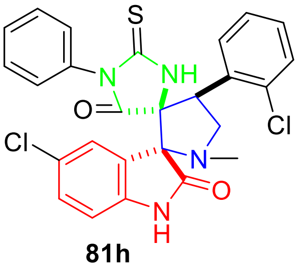

81h

IC_{50} = >100, >100, >100, >100 μM against LNCaP ($p53^{+}$), PC3 ($p53^{-}$), HCT^{wt} ($p53^{+}$), and $HCT^{-/-}$ ($p53^{-}$), respectively

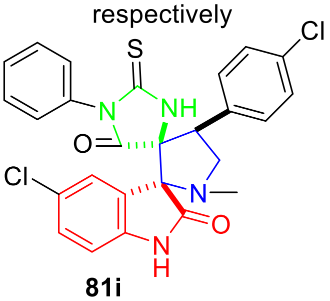

81i

IC_{50} = >100, >100, >100, >100 μM against LNCaP ($p53^{+}$), PC3 ($p53^{-}$), HCT^{wt} ($p53^{+}$), and $HCT^{-/-}$ ($p53^{-}$), respectively

Fig. S12. Antiproliferation properties of dispirooxindole-pyrrolidines **81** and nutlin-3a.

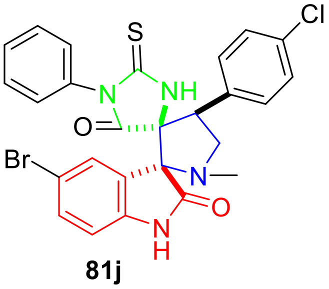

81j

IC_{50} = 1.2 ± 0.6, 11.3 ± 1.2, 3.5 ± 1.2, >100 μM against LNCaP ($p53^{+}$), PC3 ($p53^{-}$), HCT^{wt} ($p53^{+}$), and $HCT^{-/-}$ ($p53^{-}$), respectively

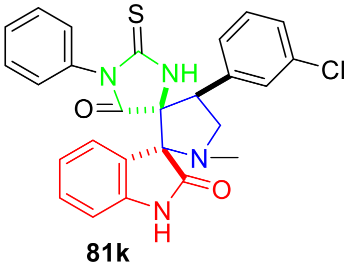

81k

IC_{50} = >100, >100 μM against LNCaP ($p53^{+}$), and PC3 ($p53^{-}$), respectively

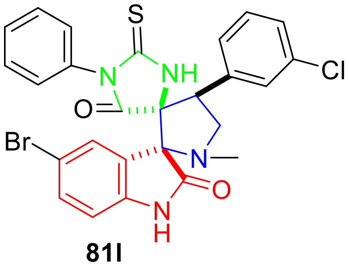

81l

IC_{50} = >100, >100, >100, >100 μM against LNCaP ($p53^{+}$), PC3 ($p53^{-}$), HCT^{wt} ($p53^{+}$), and $HCT^{-/-}$ ($p53^{-}$), respectively

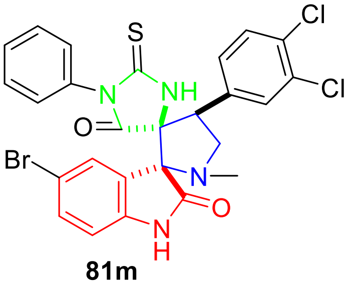

81m

IC_{50} = >100, >100, 7.6 ± 1.6, 8.8 ± 3.5 μM against LNCaP ($p53^{+}$), PC3 ($p53^{-}$), HCT^{wt} ($p53^{+}$), and $HCT^{-/-}$ ($p53^{-}$), respectively

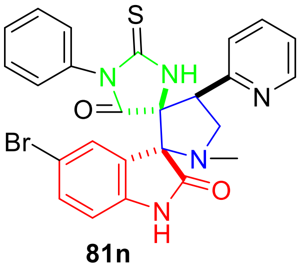

81n

IC_{50} = >100, >100, 5.6 ± 2.3, 7.6 ± 3.2 μM against LNCaP ($p53^{+}$), PC3 ($p53^{-}$), HCT^{wt} ($p53^{+}$), and $HCT^{-/-}$ ($p53^{-}$), respectively

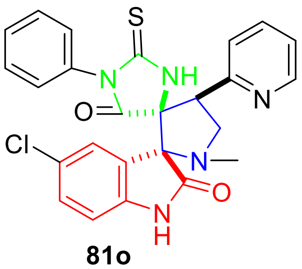

81o

IC_{50} = 2.1 ± 0.3, 8.2 ± 3.5, 60.1 ± 8.6, >100 μM against LNCaP ($p53^{+}$), PC3 ($p53^{-}$), HCT^{wt} ($p53^{+}$), and $HCT^{-/-}$ ($p53^{-}$), respectively

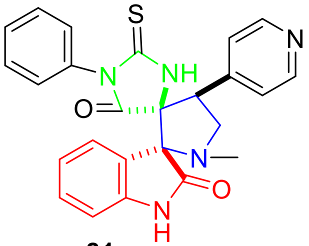

81p

IC_{50} = >100, >100, >100, >100 μM against LNCaP ($p53^{+}$), PC3 ($p53^{-}$), HCT^{wt} ($p53^{+}$), and $HCT^{-/-}$ ($p53^{-}$), respectively

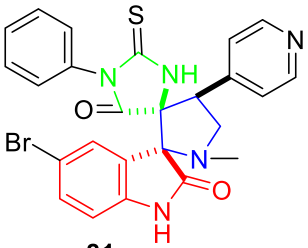

81q

IC_{50} = >100, >100, >100, >100 μM against LNCaP ($p53^{+}$), PC3 ($p53^{-}$), HCT^{wt} ($p53^{+}$), and $HCT^{-/-}$ ($p53^{-}$), respectively

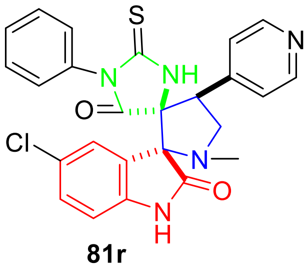

81r

IC_{50} = >100, >100, 50.± 10.2, >100 μM against LNCaP ($p53^{+}$), PC3 ($p53^{-}$), HCT^{wt} ($p53^{+}$), and $HCT^{-/-}$ ($p53^{-}$), respectively

Fig. S12 (continued)**.** Antiproliferation properties of dispirooxindole-pyrrolidines **81** and nutlin-3a.

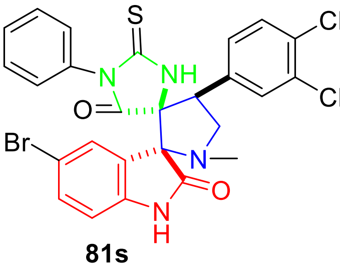

81s

IC_{50} = >100, >100, 20.0 ± 8.9, 50.0 ± 10.4 μM against LNCaP ($p53^+$), PC3 ($p53^-$), HCT^{wt} ($p53^+$), and $HCT^{-/-}$ ($p53^-$), respectively

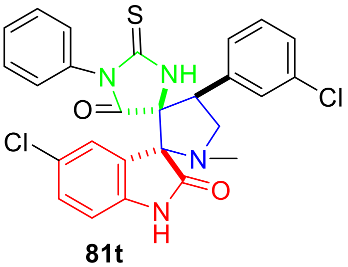

81t

IC_{50} = >100, >100, 50.7 ± 8.9, 50.7 ± 12.6 μM against LNCaP ($p53^+$), PC3 ($p53^-$), HCT^{wt} ($p53^+$), and $HCT^{-/-}$ ($p53^-$), respectively

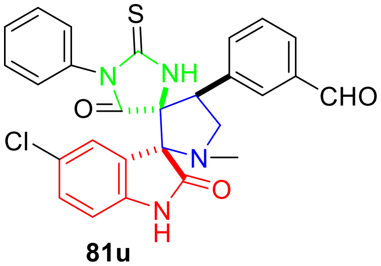

81u

IC50 = >100, >100, >100, >100 mM against LNCaP ($p53^+$), PC3 ($p53^-$), HCTwt ($p53^+$), and $HCT^{-}/^{-}$ ($p53^-$), respectively

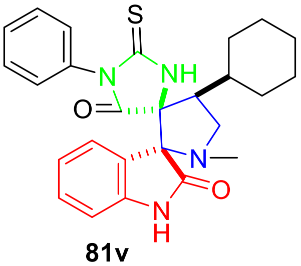

81v

IC50 = >100, >100 μM against LNCaP ($p53^+$), and PC3 ($p53^-$), respectively

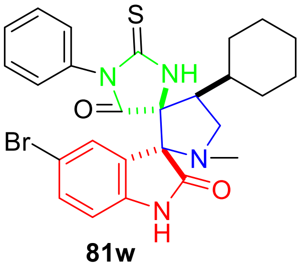

81w

IC50 = >100, >100 μM against LNCaP ($p53^+$), and PC3 ($p53^-$), respectively

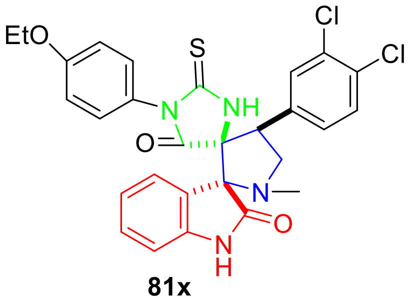

81x

IC_{50} = >100, >100, >100, >100 μM against LNCaP ($p53^+$), PC3 ($p53^-$), HCT^{wt} ($p53^+$), and $HCT^{-/-}$ ($p53^-$), respectively

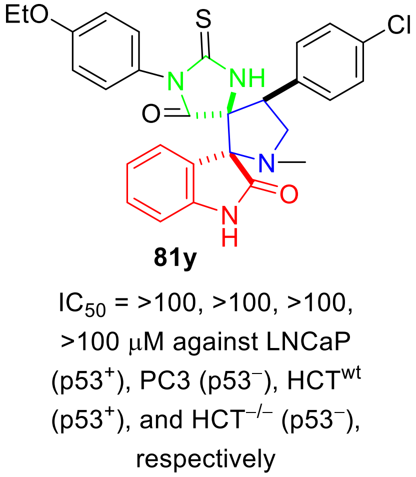

81y

IC_{50} = >100, >100, >100, >100 μM against LNCaP ($p53^+$), PC3 ($p53^-$), HCT^{wt} ($p53^+$), and $HCT^{-/-}$ ($p53^-$), respectively

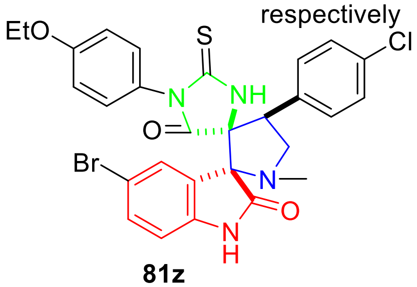

81z

IC_{50} = 3.5 ± 1.9, 9.8 ± 3.7, 14.1 ± 4.2, 70.5 ± 15.2 μM against LNCaP ($p53^+$), PC3 ($p53^-$), HCT^{wt} ($p53^+$), and $HCT^{-/-}$ ($p53^-$), respectively

Fig. S12 (continued)**.** Antiproliferation properties of dispirooxindole-pyrrolidines **81** and nutlin-3a.

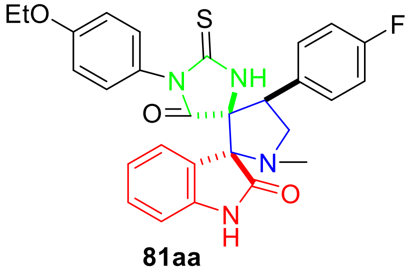

81aa

IC_{50} = >100, >100, >100, >100 μM against LNCaP (p53^{+}), PC3 (p53^{-}), HCT^{wt} (p53^{+}), and HCT$^{-/-}$ (p53^{-}), respectively

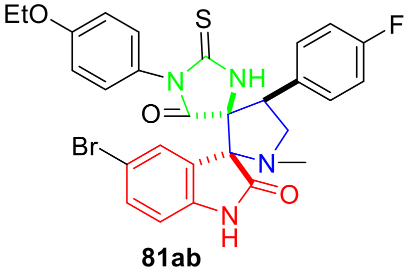

81ab

IC_{50} = >100, >100, >100, >100 μM against LNCaP (p53^{+}), PC3 (p53^{-}), HCT^{wt} (p53^{+}), and HCT$^{-/-}$ (p53^{-}), respectively

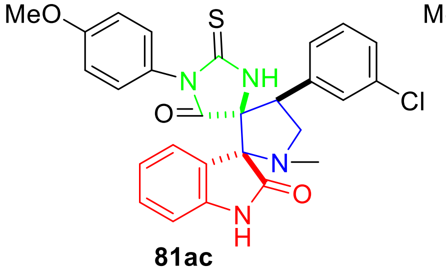

81ac

IC_{50} = >100, >100, >100, >100 μM against LNCaP (p53^{+}), PC3 (p53^{-}), HCT^{wt} (p53^{+}), and HCT$^{-/-}$ (p53^{-}), respectively

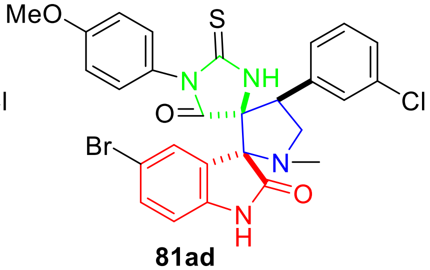

81ad

IC_{50} = >100, >100, >100, >100 μM against LNCaP (p53^{+}), PC3 (p53^{-}), HCT^{wt} (p53^{+}), and HCT$^{-/-}$ (p53^{-}), respectively

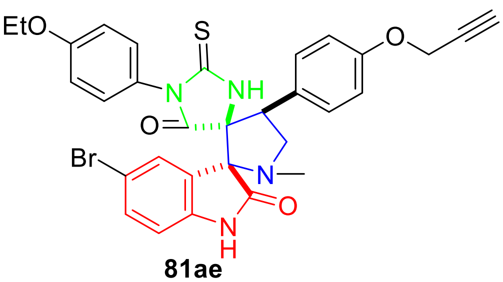

81ae

IC_{50} = >100, >100, >100, >100 μM against LNCaP (p53^{+}), PC3 (p53^{-}), HCT^{wt} (p53^{+}), and HCT$^{-/-}$ (p53^{-}), respectively

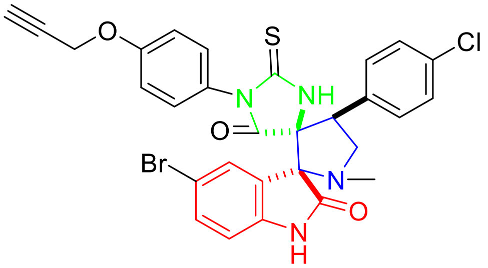

81af

IC_{50} = >100, >100, >100, >100 μM against LNCaP (p53^{+}), PC3 (p53^{-}), HCT^{wt} (p53^{+}), and HCT$^{-/-}$ (p53^{-}), respectively

Fig. S12 (continued)**.** Antiproliferation properties of dispirooxindole-pyrrolidines **81** and nutlin-3a.

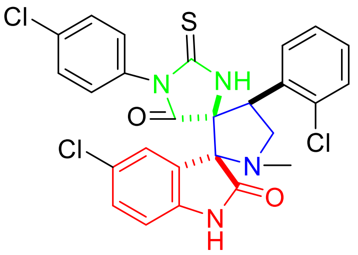

81ag

IC_{50} = >100, >100, >100, >100 μM against LNCaP ($p53^+$), PC3 ($p53^-$), HCT^{wt} ($p53^+$), and $HCT^{-/-}$ ($p53^-$), respectively

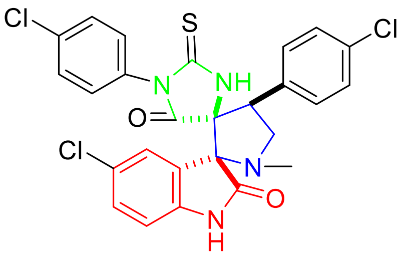

81ah

IC_{50} = >100, >100, >100, >100 μM against LNCaP ($p53^+$), PC3 ($p53^-$), HCT^{wt} ($p53^+$), and $HCT^{-/-}$ ($p53^-$), respectively

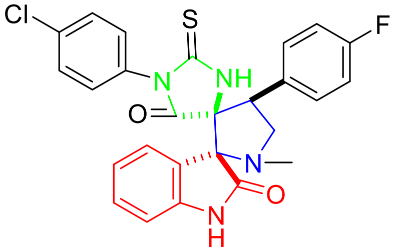

81ai

IC_{50} = >100, >100, >100, >100 μM against LNCaP ($p53^+$), PC3 ($p53^-$), HCT^{wt} ($p53^+$), and $HCT^{-/-}$ ($p53^-$), respectively

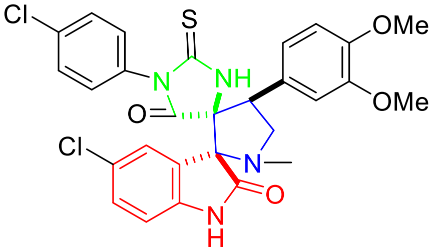

81aj

IC_{50} = >100, >100, >100, >100 μM against LNCaP ($p53^+$), PC3 ($p53^-$), HCT^{wt} ($p53^+$), and $HCT^{-/-}$ ($p53^-$), respectively

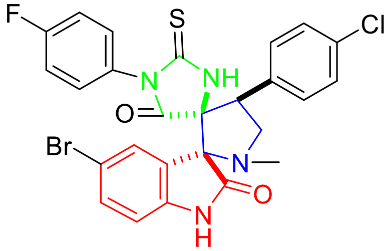

81ak

IC_{50} = >100, >100, >100, >100 μM against LNCaP ($p53^+$), PC3 ($p53^-$), HCT^{wt} ($p53^+$), and $HCT^{-/-}$ ($p53^-$), respectively

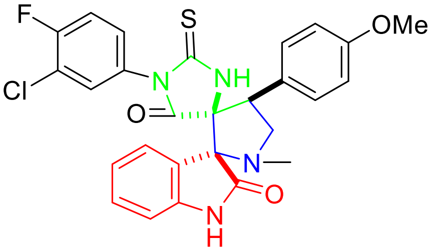

81al

IC_{50} = >100, >100, >100, >100 μM against LNCaP ($p53^+$), PC3 ($p53^-$), HCT^{wt} ($p53^+$), and $HCT^{-/-}$ ($p53^-$), respectively

Fig. S12 (continued)**.** Antiproliferation properties of dispirooxindole-pyrrolidines **81** and nutlin-3a.

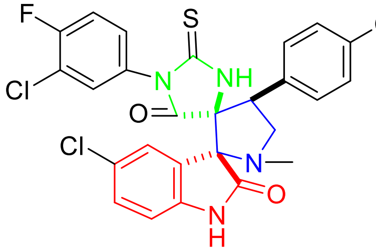

81am

IC_{50} = >100, >100, >100, >100 μM against LNCaP ($p53^+$), PC3 ($p53^-$), HCT^{wt} ($p53^+$), and $HCT^{-/-}$ ($p53^-$), respectively

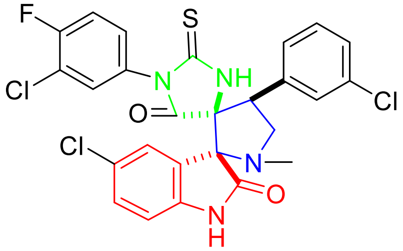

81an

IC_{50} = 3.2 ± 1.6, 5.7 ± 2.6, 9.3 ± 4.6, 9.5 ± 4.9 μM against LNCaP ($p53^+$), PC3 ($p53^-$), HCT^{wt} ($p53^+$), and $HCT^{-/-}$ ($p53^-$), respectively

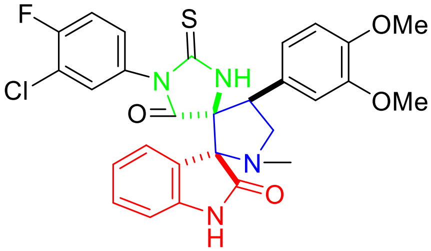

81ao

IC_{50} = 8.6 ± 1.8, 10.2 ± 2.3, 35.0 ± 16.2, >100 μM against LNCaP ($p53^+$), PC3 ($p53^-$), HCT^{wt} ($p53^+$), and $HCT^{-/-}$ ($p53^-$), respectively

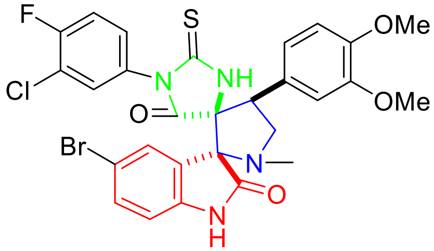

81ap

IC_{50} = >100, >100 μM against LNCaP ($p53^+$), and PC3 ($p53^-$), respectively

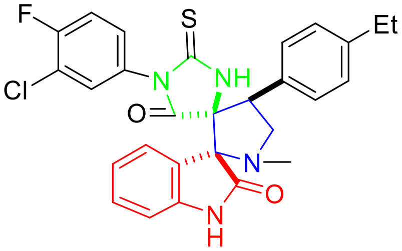

81aq

IC_{50} = >100, >100, 6.3 ± 2.5, 6.5 ± 3.6 μM against LNCaP ($p53^+$), PC3 ($p53^-$), HCT^{wt} ($p53^+$), and $HCT^{-/-}$ ($p53^-$), respectively

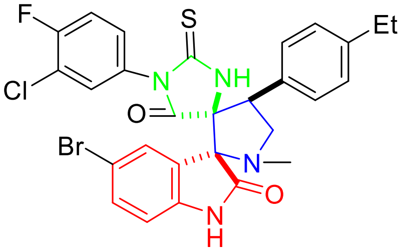

81ar

IC_{50} = >100, >100, >100, >100 μM against LNCaP ($p53^+$), PC3 ($p53^-$), HCT^{wt} ($p53^+$), and $HCT^{-/-}$ ($p53^-$), respectively

Fig. S12 (continued)**.** Antiproliferation properties of dispirooxindole-pyrrolidines **81** and nutlin-3a.

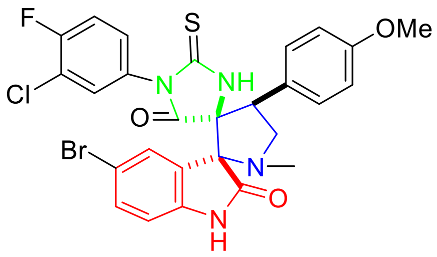

81as

IC_{50} = >100, >100, >100, >100 μM against LNCaP ($p53^{+}$), PC3 ($p53^{-}$), HCT^{wt} ($p53^{+}$), and $HCT^{-/-}$ ($p53^{-}$), respectively

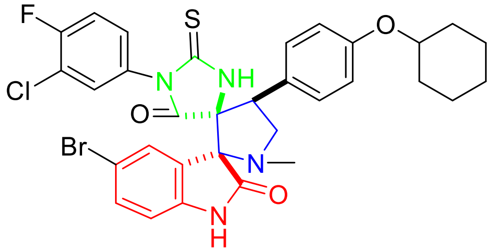

81at

IC_{50} = >100, >100 μM against LNCaP ($p53^{+}$), and PC3 ($p53^{-}$), respectively

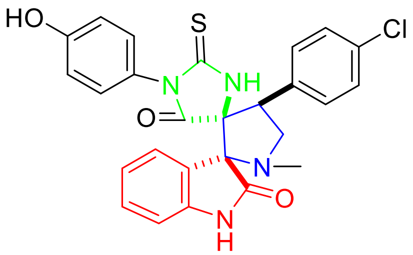

81au

IC_{50} = 18.0 ± 4.7, 4.65 ± 2.3, 6.9 ± 3.1, >100 μM against LNCaP ($p53^{+}$), PC3 ($p53^{-}$), HCT^{wt} ($p53^{+}$), and $HCT^{-/-}$ ($p53^{-}$), respectively

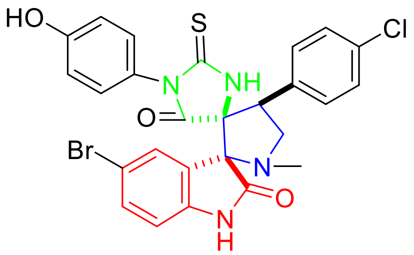

81av

IC_{50} = >100, >100, >100, >100 μM against LNCaP ($p53^{+}$), PC3 ($p53^{-}$), HCT^{wt} ($p53^{+}$), and $HCT^{-/-}$ ($p53^{-}$), respectively

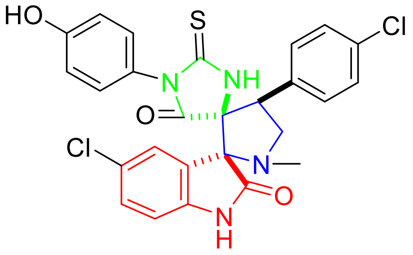

81aw

IC_{50} = >100, >100, >100, >100 μM against LNCaP ($p53^{+}$), PC3 ($p53^{-}$), HCT^{wt} ($p53^{+}$), and $HCT^{-/-}$ ($p53^{-}$), respectively

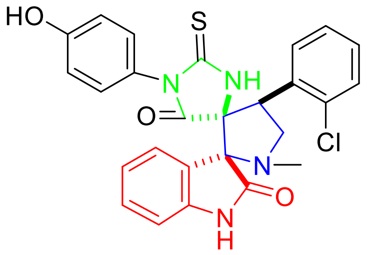

81ax

IC_{50} = >100, >100, >100, >100 μM against LNCaP ($p53^{+}$), PC3 ($p53^{-}$), HCT^{wt} ($p53^{+}$), and $HCT^{-/-}$ ($p53^{-}$), respectively

Fig. S12 (continued)**.** Antiproliferation properties of dispirooxindole-pyrrolidines **81** and nutlin-3a.

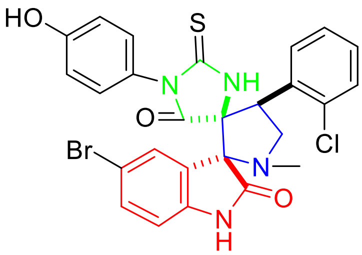

81ay

IC_{50} = 9.5 ± 2.5, 20.0 ± 8.4, >100, >100 μM against LNCaP ($p53^+$), PC3 ($p53^-$), HCT^{wt} ($p53^+$), and $HCT^{-/-}$ ($p53^-$), respectively

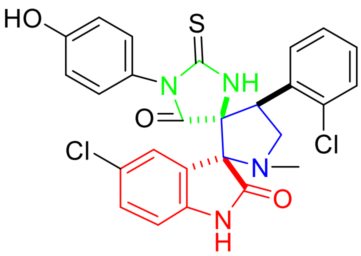

81az

IC_{50} = 7.0 ± 1.0, 15.1 ± 1.2, >100, >100 μM against LNCaP ($p53^+$), PC3 ($p53^-$), HCT^{wt} ($p53^+$), and $HCT^{-/-}$ ($p53^-$), respectively

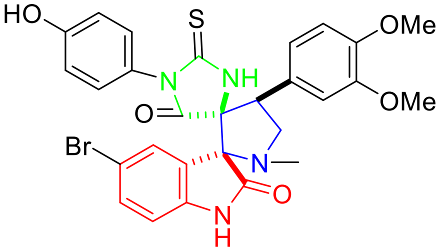

81ba

IC_{50} = 9.0 ± 2.0, 30.0 ± 10.5, >100, >100 μM against LNCaP ($p53^+$), PC3 ($p53^-$), HCT^{wt} ($p53^+$), and $HCT^{-/-}$ ($p53^-$), respectively

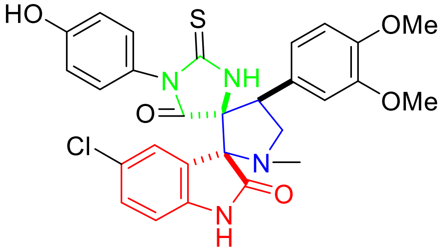

81bb

IC_{50} = 17.0 ± 1.0, 20.0 ± 6.9, >100, >100 μM against LNCaP ($p53^+$), PC3 ($p53^-$), HCT^{wt} ($p53^+$), and $HCT^{-/-}$ ($p53^-$), respectively

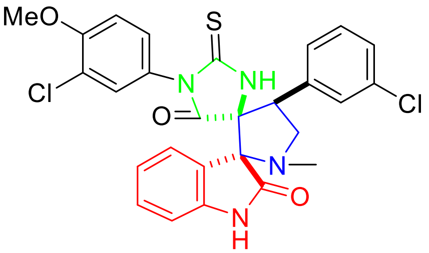

81bc

IC_{50} = >100, >100 μM against LNCaP ($p53^+$), and PC3 ($p53^-$), respectively

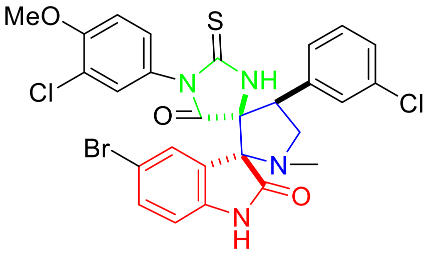

81bd

IC_{50} = >50, >50 μM against LNCaP ($p53^+$), and PC3 ($p53^-$), respectively

Fig. S12 (continued)**.** Antiproliferation properties of dispirooxindole-pyrrolidines **81** and nutlin-3a.

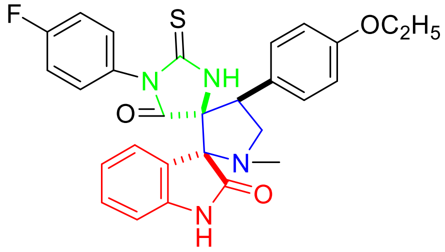

81be

IC$_{50}$ = >100, >100 μM against LNCaP (p53$^+$), and PC3 (p53$^-$), respectively

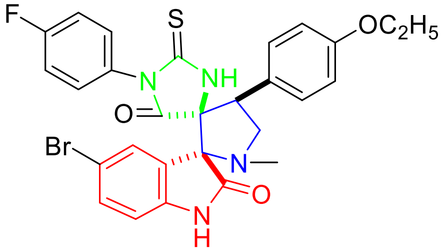

81bf

IC$_{50}$ = >100, >100 μM against LNCaP (p53$^+$), and PC3 (p53$^-$), respectively

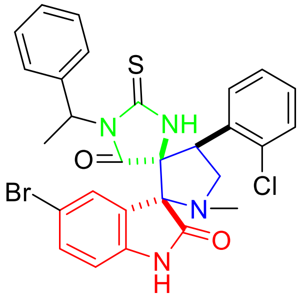

81bg

IC$_{50}$ = >100, >100, >100, >100 μM against LNCaP (p53$^+$), PC3 (p53$^-$), HCT^{wt} (p53$^+$), and HCT$^{-/-}$ (p53$^-$), respectively

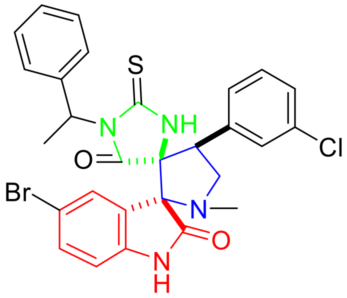

81bh

IC$_{50}$ = 3.4 ± 1.5, 8.8 ± 3.1, 8.0 ± 2.5, >100 μM against LNCaP (p53$^+$), PC3 (p53$^-$), HCT^{wt} (p53$^+$), and HCT$^{-/-}$ (p53$^-$), respectively

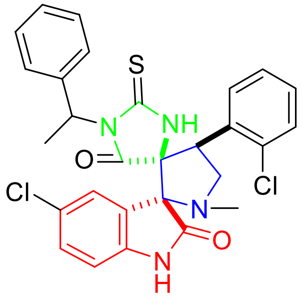

81bi

IC$_{50}$ = >100, >100, 24.0 ± 6.7, >100 μM against LNCaP (p53$^+$), PC3 (p53$^-$), HCT^{wt} (p53$^+$), and HCT$^{-/-}$ (p53$^-$), respectively

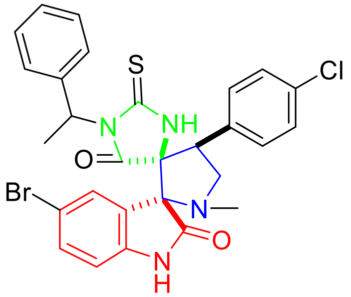

81bj

IC$_{50}$ = >100, >100, >100, >100 μM against LNCaP (p53$^+$), PC3 (p53$^-$), HCT^{wt} (p53$^+$), and HCT$^{-/-}$ (p53$^-$), respectively

Fig. S12 (continued)**.** Antiproliferation properties of dispirooxindole-pyrrolidines **81** and nutlin-3a.

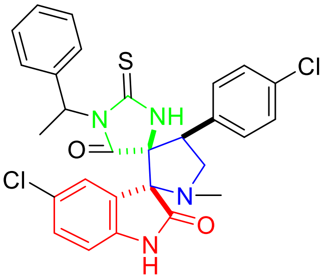

81bk

IC_{50} = >100, >100,
>100, >100 μM
against LNCaP ($p53^+$),
PC3 ($p53^-$), HCT^{wt} ($p53^+$),
and $HCT^{-/-}$ ($p53^-$), respectively

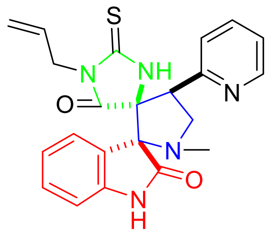

81bl

IC_{50} = >50, >50 μM
against LNCaP ($p53^+$),
and PC3 ($p53^-$), respectively

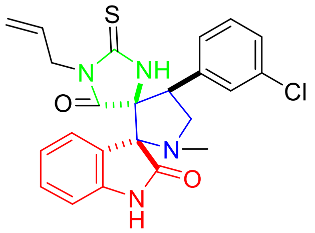

81bm

IC_{50} = >50, >50 μM
against LNCaP ($p53^+$),
and PC3 ($p53^-$), respectively

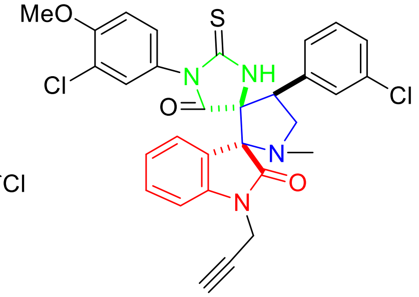

81bn

IC_{50} = >100, >100,
>100, >100 μM
against LNCaP ($p53^+$),
PC3 ($p53^-$), HCT^{wt} ($p53^+$),
and $HCT^{-/-}$ ($p53^-$), respectively

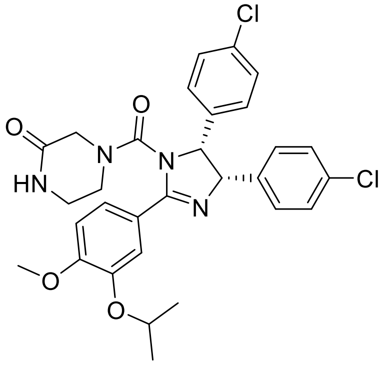

Nutlin-3a

IC_{50} = 2.7 ± 0.9, 28.9 ± 3.7,
4.5 ± 1.3, >100 μM
against LNCaP ($p53^+$),
PC3 ($p53^-$), HCT^{wt} ($p53^+$),
and $HCT^{-/-}$ ($p53^-$), respectively

Fig. S12 (continued)**.** Antiproliferation properties of dispirooxindole-pyrrolidines **81** and nutlin-3a.

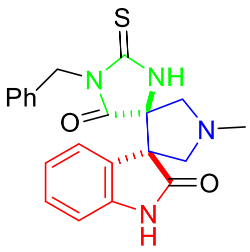

84a

CC_{50} = 20.3 ± 3.7, 31.3 ± 6.7, 16.1 ± 2.5, 9.8 ± 1.6, 66.2 ± 11.74, 40.11 ± 19.11, 12.5 ± 2.1, 30.1 ± 9.1 μM against A549, MCF7, VA13, Hek293T, $HCT^{+/+}$, $HCT^{-/-}$, LNCaP, and PC3, respectively

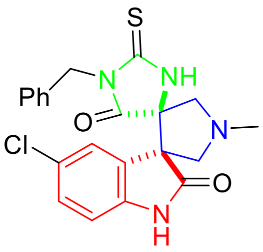

84b

CC_{50} = 9.1 ± 2.2, 24.3 ± 2.3, 21.4 ± 1.9, 9.5 ± 1.7, 60.73 ± 13.75, 30.01 ± 15.21, 10.3 ± 1.1, 49.0 ± 12.7 μM against A549, MCF7, VA13, Hek293T, $HCT^{+/+}$, $HCT^{-/-}$, LNCaP, and PC3, respectively

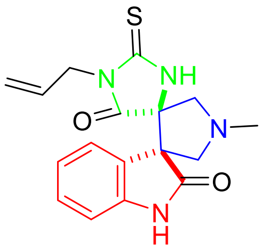

84c

CC_{50} = 45.9 ± 13.4, 41.8 ± 12.4, 14.33 ± 6.65, 9.8 ± 3.3, 53.1 ± 15.8 μM against A549, Hek293T, $HCT^{-/-}$, LNCaP, and PC3, respectively

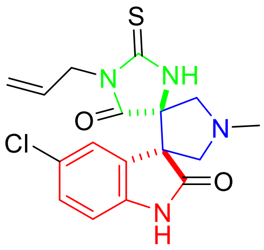

84d

CC_{50} = 32.1 ± 6.7, 68.4 ± 8.8, 30.8 ± 4.5, 11.5 ± 3, 57.73 ± 18.41, 40.11 ± 5.23, 20.0 ± 3.6, 50.0 ± 9.1 μM against A549, MCF7, VA13, Hek293T, $HCT^{+/+}$, $HCT^{-/-}$, LNCaP, and PC3, respectively

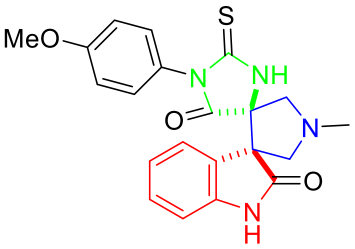

84e

CC_{50} = 18.9 ± 3.1, 22.8 ± 5.6, 13.2 ± 2.3, 6.5 ± 0.9, 27.9 ± 6.94, 36.0 ± 12.55, 3.45 ± 0.45, 50.0 ± 9.1 μM against A549, MCF7, VA13, Hek293T, $HCT^{+/+}$, $HCT^{-/-}$, and LNCaP, respectively

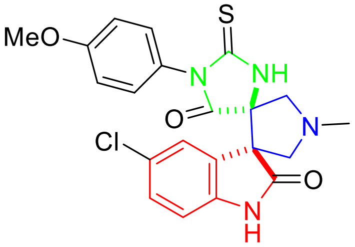

84f

CC_{50} = 2.8 ± 0.4, 2.3 ± 0.5, 2.3 ± 0.4, 1.1 ± 0.1 μM against A549, MCF7, VA13, and Hek293T, respectively

Fig. S13. Antiproliferation properties of dispirooxindole-pyrrolidines **84,** etoposide, and nutlin-3.

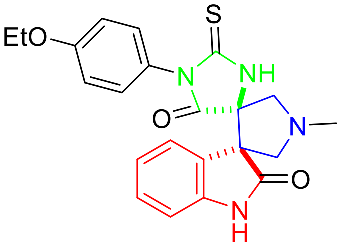

84g

CC_{50} = 38.2 $\pm$ 11.5, 75.2 $\pm$ 29.3, 73.6 $\pm$ 23.4, 30.2 $\pm$ 8.2 μM against A549, MCF7, VA13 and Hek293T, respectively

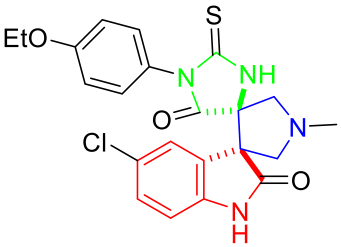

84h

CC_{50} = 6.2 $\pm$ 2.1, 8.5 $\pm$ 1.2, 6.3 $\pm$ 1.1, 6.7 $\pm$ 0.9 μM against A549, MCF7, VA13 and Hek293T, respectively

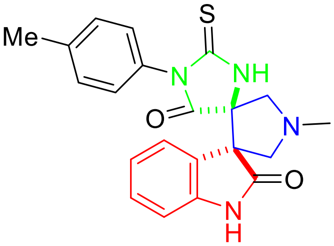

84i

CC_{50} = 88.2 $\pm$ 32.2 μM against MCF7

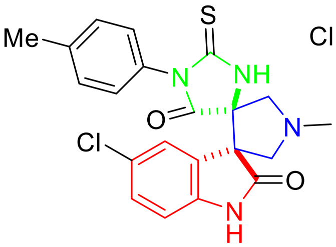

84j

CC_{50} = 62 $\pm$ 8.3, 13.7 $\pm$ 2.1, 27.2 $\pm$ 5.3, 17.4 $\pm$ 2 μM against A549, MCF7, VA13 and Hek293T, respectively

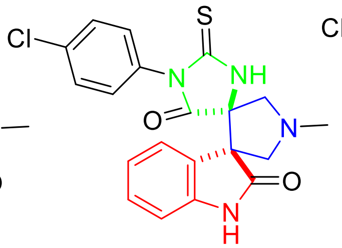

84k

CC_{50} = 67.5 $\pm$ 6.9, 27.9 $\pm$ 6.9, 59.1 $\pm$ 7.6, 69 $\pm$ 3.7, 52.7 $\pm$ 11.4, 40.9 $\pm$ 20.5, 5.2 $\pm$ 0.91, 7.8 $\pm$ 1.4 μM against A549, MCF7, VA13, Hek293T, $HCT^{+/+}$, $HCT^{–/–}$, LNCaP, and PC3, respectively

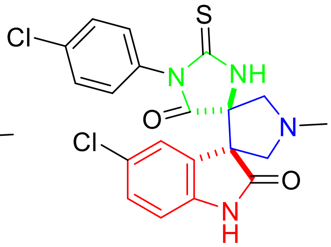

84l

CC_{50} = 37 $\pm$ 6.5, 19.1 $\pm$ 4.1, 30.4 $\pm$ 3, 34 $\pm$ 3.4, 42.5 $\pm$ 9.1, 57.11 $\pm$ 5.76, 4.4 $\pm$ 0.67, 2.2 $\pm$ 0.7 μM against A549, MCF7, VA13, Hek293T, $HCT^{+/+}$, $HCT^{–/–}$, LNCaP, and PC3, respectively

Fig. S13 (continued)**.** Antiproliferation properties of dispirooxindole-pyrrolidines **84,** etoposide, and nutlin-3.

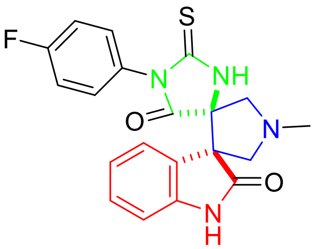

84m

CC_{50} = 105.9 ± 43.1, 171.0 ± 15.8, 23.3 ± 3.3, 42.4 ± 4.7, 3.4 ± 0.7, 1.93 ± 0.91 μM against MCF7, VA13, $HCT^{+/+}$, $HCT^{-/-}$, LNCaP, and PC3, respectively

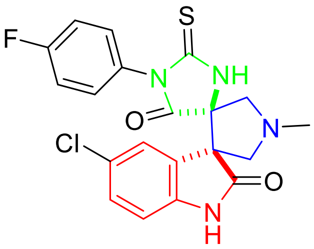

84n

CC_{50} = 53.0 ± 9.5 μM against Hek293T

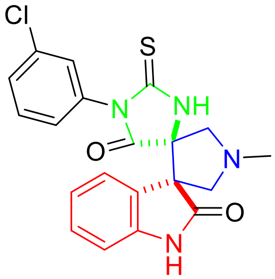

84o

CC_{50} = 53.4 ± 11.2, 23.2 ± 3.6, 36.3 ± 3.2, 48 ± 5.3 μM against A549, MCF7, VA13, and Hek293T, respectively

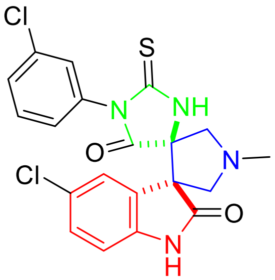

84p

CC_{50} = 11.3 ± 2.4, 6.6 ± 1, 12.3 ± 1.6, 18.4 ± 1.7, 34.2 ± 5.7, 4.1 ± 0.1, 2.2 ± 1.4 μM against A549, MCF7, VA13, Hek293T, $HCT^{+/+}$, LNCaP, and PC3, respectively

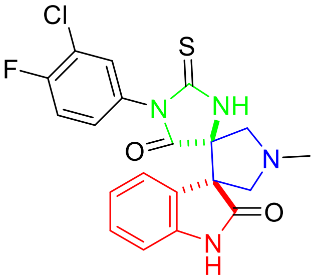

84q

CC_{50} = 62.2 ± 27.7, 57.9 ± 18.3, 69.1 ± 21 μM against MCF7, VA13, and Hek293T, respectively

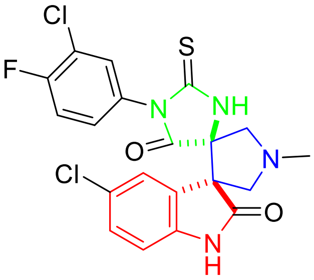

84r

CC_{50} = 14.1 ± 1.8, 14.1 ± 1.2, 13.9 ± 1.5, 11.1 ± 1.3 μM against A549, MCF7, VA13, and Hek293T, respectively

Fig. S13 (continued)**.** Antiproliferation properties of dispirooxindole-pyrrolidines **84,** etoposide, and nutlin-3.

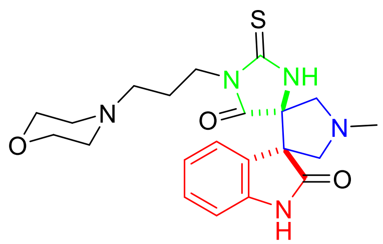

84s

CC_{50} = 20.8 ± 4.8, 3.4 ± 0.6, 53.6 ± 17.3 μM against HCT$^{+/+}$, LNCaP, and PC3, respectively

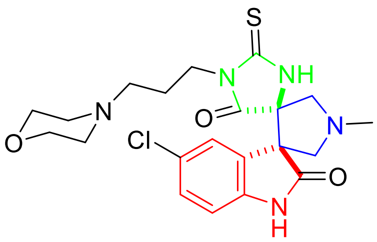

84t

CC_{50} = 103.2 ± 27.7 μM against Hek293T, respectively

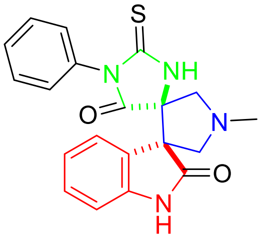

84u

CC_{50} = 57.9 ± 17.1, 94.1 ± 19.7, 75.1 ± 8.6, 54.7 ± 9.5, 9.8 ± 1.8, 52.2 ± 11.6 μM against MCF7, VA13, Hek293T, HCT$^{+/+}$, LNCaP, and PC3, respectively

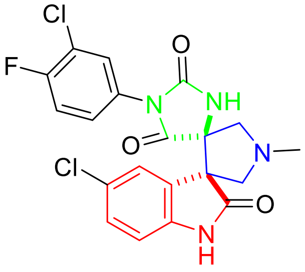

84v

CC_{50} = 35.3 ± 17.7, 15.1 ± 1.2, 19.6 ± 1.8, >50 μM against A549, Hek293T, LNCaP, and PC3, respectively

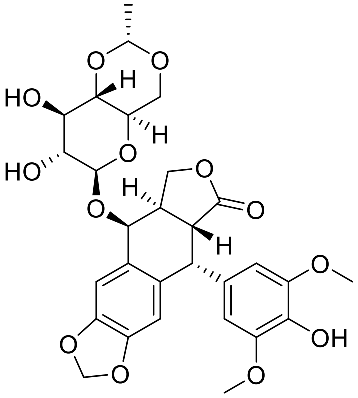

Etoposide

CC_{50} = 0.3 ± 0.1, 2.6 ± 0.9, 1.1 ± 0.2, 0.3 ± 0.1, 0.43 ± 0.12, 0.85 ± 0.22 μM against A549, MCF7, VA13, Hek293T, HCT$^{+/+}$, and HCT$^{-/-}$, respectively

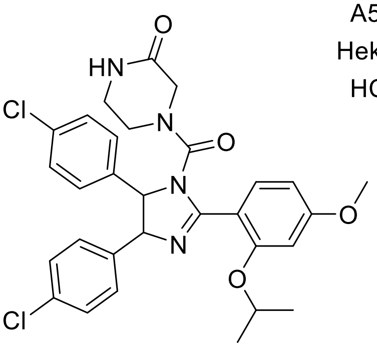

Nutlin-3

CC_{50} = 3.3 ± 0.13, 35.12 ± 2.65 μM against HCT$^{+/+}$, andHCT$^{-/-}$, respectively

Fig. S13 (continued)**.** Antiproliferation properties of dispirooxindole-pyrrolidines **84,** etoposide, and nutlin-3.

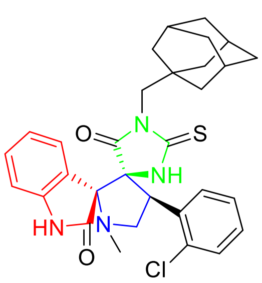

89a

IC_{50} = 15.46 ± 14.6, 9.72 ± 7.93, 13.58 ± 13.08, 7.41 ± 2.78 μM against A549, HEK293T, MCF7, and VA13, respectively

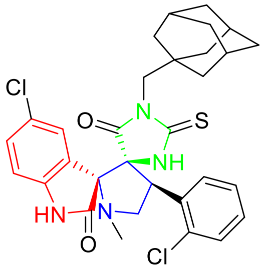

89b

IC_{50} = 9.33 ± 6.9, 8.33 ± 6.76, 3.23 ± 3.02, 9.01 ± 7.11 μM against A549, HEK293T, MCF7, and VA13, respectively

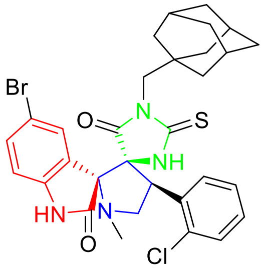

89c

IC_{50} = 7.2 ± 1.85, 7.63 ± 0.75, 2.79 ± 2.75, 17.26 ± 14.76 μM against A549, HEK293T, MCF7, and VA13, respectively

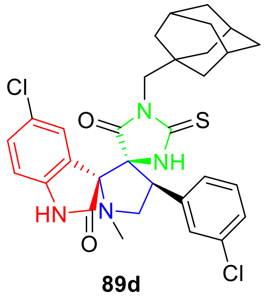

89d

IC_{50} = 14.87 ± 14.28, 20.08 ± 18.11, 12.67 ± 12.36, 29.41 ± 15.55 μM against A549, HEK293T, MCF7, and VA13, respectively

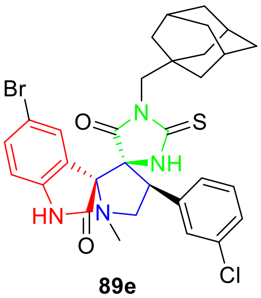

89e

IC_{50} = 10.3 ± 4.88, 11.23 ± 3.99, 20.69 ± 15.56, 46.88 ± 30.69 μM against A549, HEK293T, MCF7, and VA13, respectively

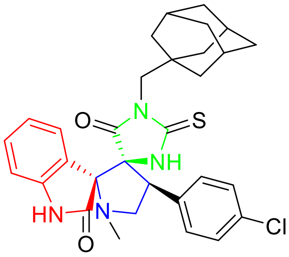

89f

IC_{50} = 7.6 ± 0.7, 7.7 ± 0.8, 4.9 ± 0.5, 18.4 ± 5.9 μM against A549, HEK293T, MCF7, and VA13, respectively

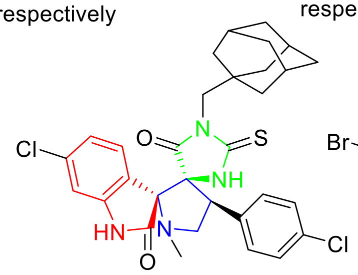

89g

IC_{50} = 8.7 ± 1.8, 9.3 ± 0.7, 7.3 ± 0.5, 9.7 ± 1.2 μM against A549, HEK293T, MCF7, and VA13, respectively

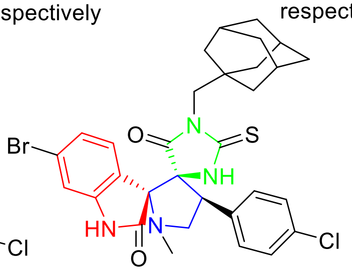

89h

IC_{50} = 10.04 ± 7.1, 10.18 ± 2.81, 13.61 ± 6.68, 35.23 ± 34.13 μM against A549, HEK293T, MCF7, and VA13, respectively

Fig. S14. Antiproliferation properties of dispirooxindole-pyrrolidines **89**, nutlin-3a and cisplatin.

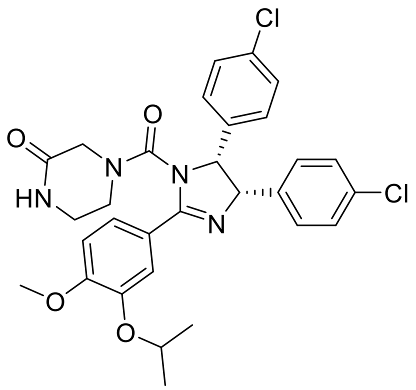

Nutlin-3a

IC_{50} = 14.9 ± 0.6,
10.4 ± 0.8
μM against A549,
and HEK293T,
respectively

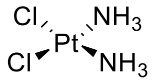

Cisplatin

IC_{50} = 44.13 ± 3.9,
12.4 ± 3.9, >50,
>50 μM against
A549, HEK293T,
MCF7, and VA13,
respectively

Fig. S14 (continued)**.** Antiproliferation properties of dispirooxindole-pyrrolidines **89**, nutlin-3a and cisplatin.

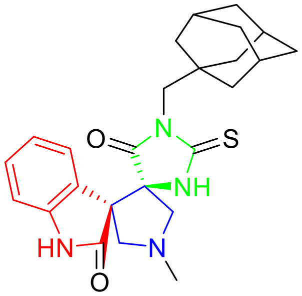

92a

IC_{50} = 6.8 ± 1.3, 5.1 ± 0.5, 5.2 ± 0.4, 5.2 ± 1.0 μM against A549, HEK293T, MCF7, and VA13, respectively

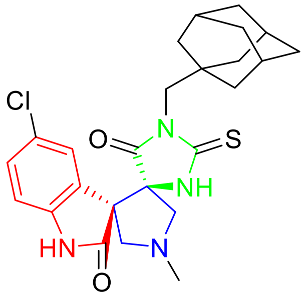

92b

IC_{50} = 5.0 ± 0.6, 2.8 ± 0.3, 2.8 ± 0.2, 2.9 ± 0.5 μM against A549, HEK293T, MCF7, and VA13, respectively

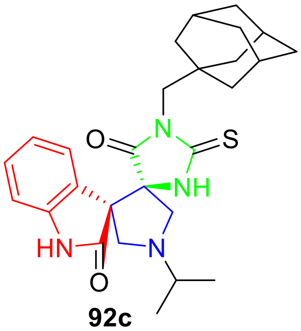

92c

IC_{50} = 9.3 ± 0.6, 5.8 ± 0.4, 5.4 ± 0.4, 5.6 ± 1.3 μM against A549, HEK293T, MCF7, and VA13, respectively

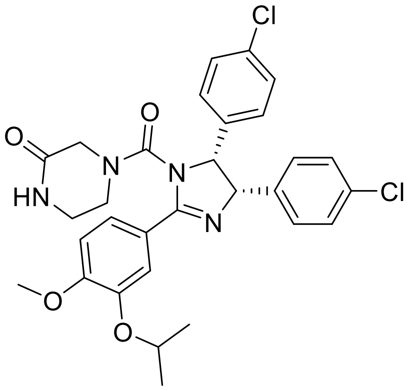

Nutlin-3a

IC_{50} = 14.9 ± 0.6, 10.4 ± 0.8 μM against A549, and HEK293T, respectively

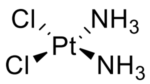

Cisplatin

IC_{50} = 44.13 ± 3.9, 12.4 ± 3.9, >50, >50 μM against A549, HEK293T, MCF7, and VA13, respectively

Fig. S15. Antiproliferation properties of dispirooxindole-pyrrolidines **92**, nutlin-3a and cisplatin.

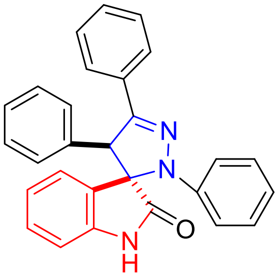

97a

IC_{50} = >20 μM against HCT116 p53$^{+/+}$

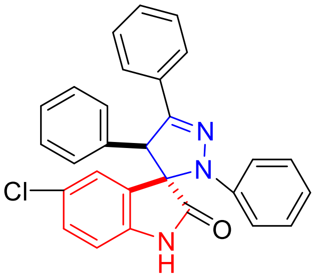

97b

IC_{50} = 11.0 ± 0.3 μM against HCT116 p53$^{+/+}$

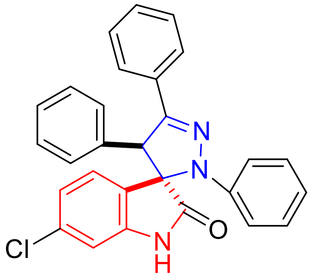

97c

IC_{50} = 16.7 ± 0.9 μM against HCT116 p53$^{+/+}$

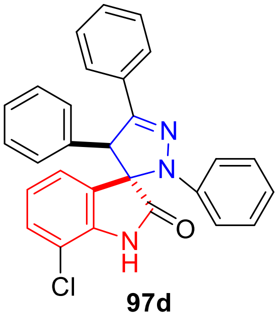

97d

IC_{50} = 16.6 ± 1.5 μM against HCT116 p53$^{+/+}$

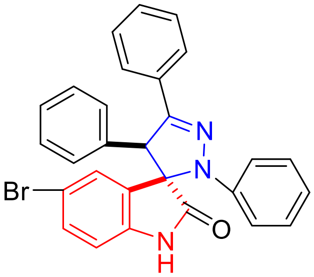

97e

IC_{50} = 13.1 ± 1.0 μM against HCT116 p53$^{+/+}$

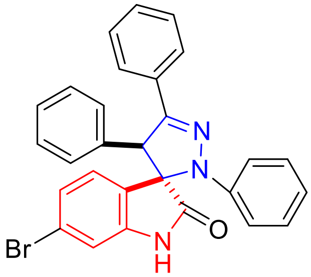

97f

IC_{50} = 15.9 ± 0.6 μM against HCT116 p53$^{+/+}$

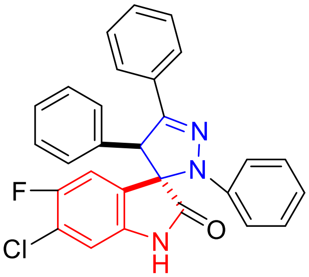

97g

IC_{50} = 12.4 ± 0.7 μM against HCT116 p53$^{+/+}$

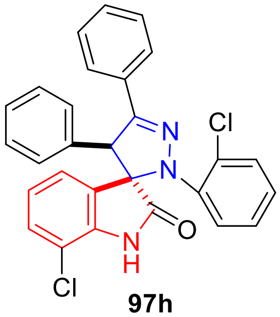

97h

IC_{50} = >20 μM against HCT116 p53$^{+/+}$

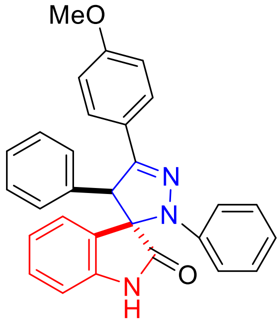

97i

IC_{50} = 11.7 ± 0.3 μM against HCT116 p53$^{+/+}$

Fig. S16. Antiproliferation properties of spirooxindole-pyrazolines **97**.

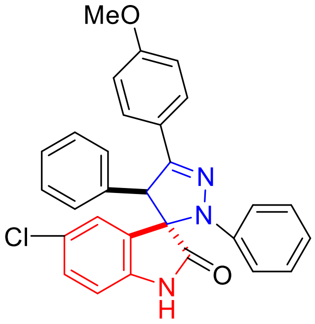

97j

IC_{50} = 10.6 ± 1.5 μM
against HCT116 $p53^{+/+}$

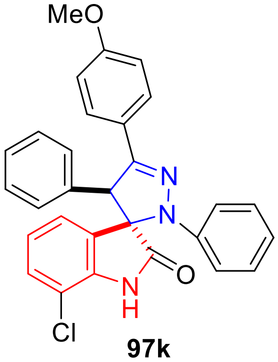

97k

IC_{50} = 15.6 ± 0.9 μM
against HCT116 $p53^{+/+}$

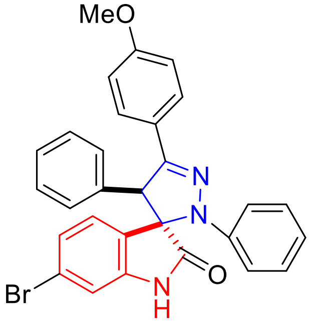

97l

IC_{50} = 12.8 ± 0.7 μM
against HCT116 $p53^{+/+}$

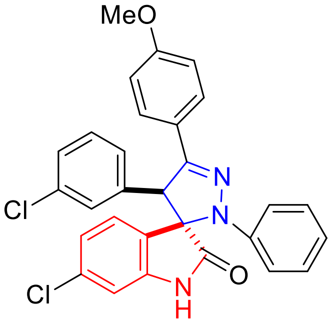

97m

IC_{50} = 10.9 ± 0.8 μM
against HCT116 $p53^{+/+}$

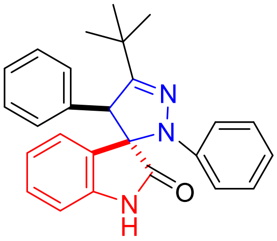

97n

IC_{50} = >20 μM
against HCT116 $p53^{+/+}$

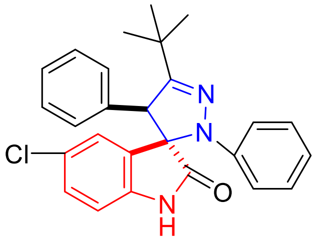

97o

IC_{50} = 17.0 ± 1.3 μM
against HCT116 $p53^{+/+}$

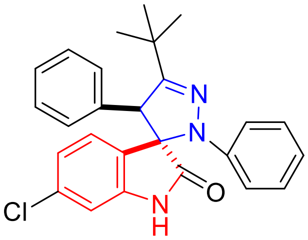

97p

IC_{50} = >20 μM
against HCT116 $p53^{+/+}$

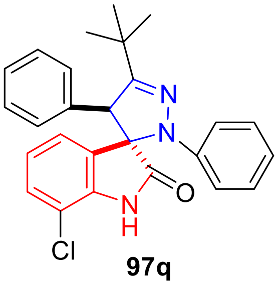

97q

IC_{50} = 13.3 ± 1.4 μM
against HCT116 $p53^{+/+}$

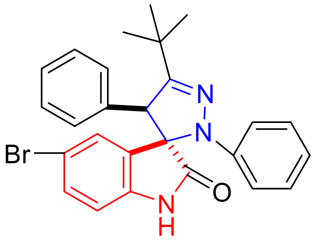

97r

IC_{50} = >20 μM
against HCT116 $p53^{+/+}$

Fig. S16 (continued)**.** Antiproliferation properties of spirooxindole-pyrazolines **97**.

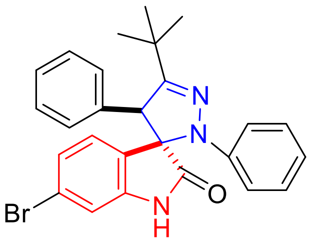

97s

IC_{50} = >20 μM
against HCT116 $p53^{+/+}$

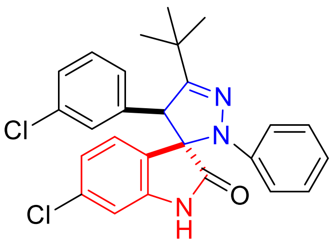

97t

IC_{50} = 18.4 ± 1.8 μM
against HCT116 $p53^{+/+}$

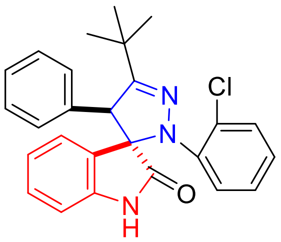

97u

IC_{50} = >20 μM
against HCT116 $p53^{+/+}$

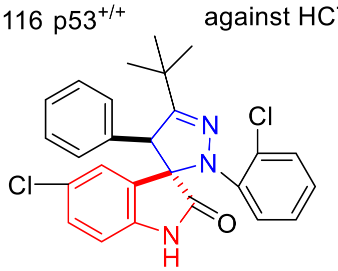

97v

IC_{50} = >20 μM
against HCT116 $p53^{+/+}$

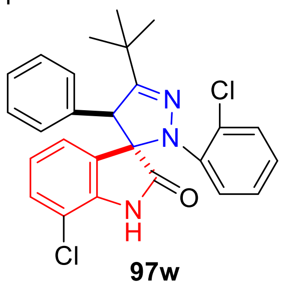

97w

IC_{50} = 17.3 ± 0.2 μM
against HCT116 $p53^{+/+}$

Fig. S16 (continued)**.** Antiproliferation properties of spirooxindole-pyrazolines **97**.

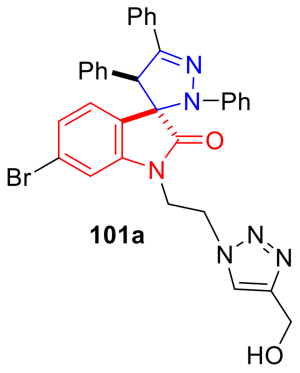

101a

IC_{50} = 11.9, 9.30 μM
against MCF7, and
MDA-MB-231,
respectively

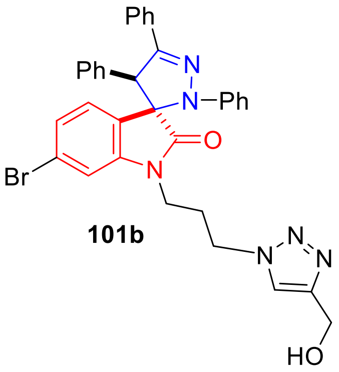

101b

IC_{50} = >40, >40 μM
against MCF7, and
MDA-MB-231,
respectively

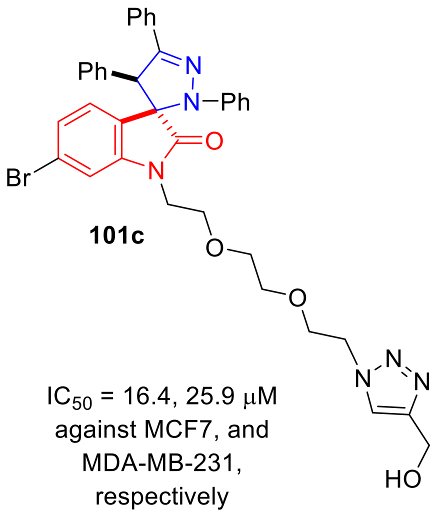

101c

IC_{50} = 16.4, 25.9 μM
against MCF7, and
MDA-MB-231,
respectively

Fig. S17. Antiproliferation properties of spirooxindole-pyrazolines linked to triazolyl heterocycle **101**.

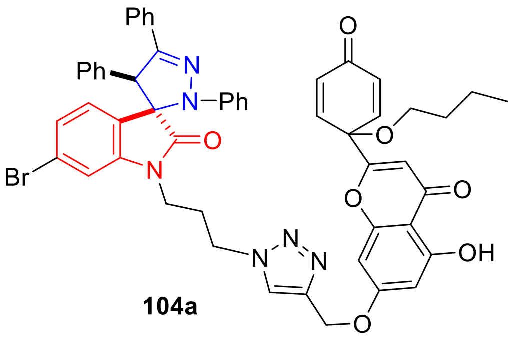

104a

IC_{50} = >40, 10.6, >40 µM against MCF7, MDA-MB-231, and MCF 10A, respectively

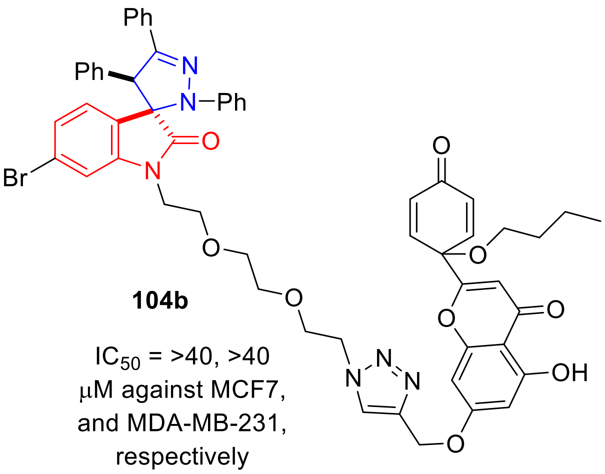

104b

IC_{50} = >40, >40 µM against MCF7, and MDA-MB-231, respectively

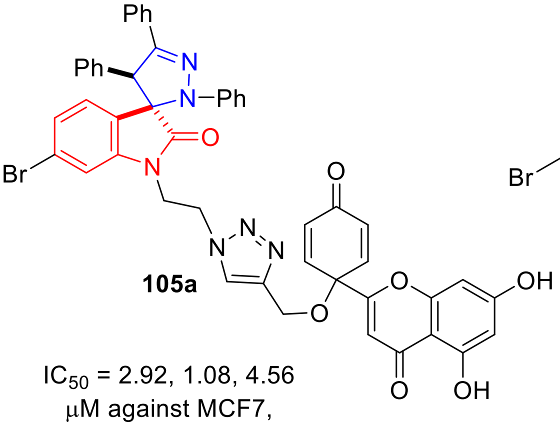

105a

IC_{50} = 2.92, 1.08, 4.56 µM against MCF7, MDA-MB-231, and MCF 10A, respectively

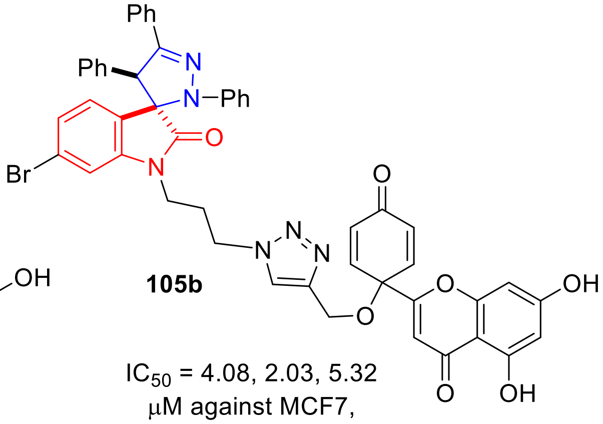

105b

IC_{50} = 4.08, 2.03, 5.32 µM against MCF7, MDA-MB-231, and MCF 10A, respectively

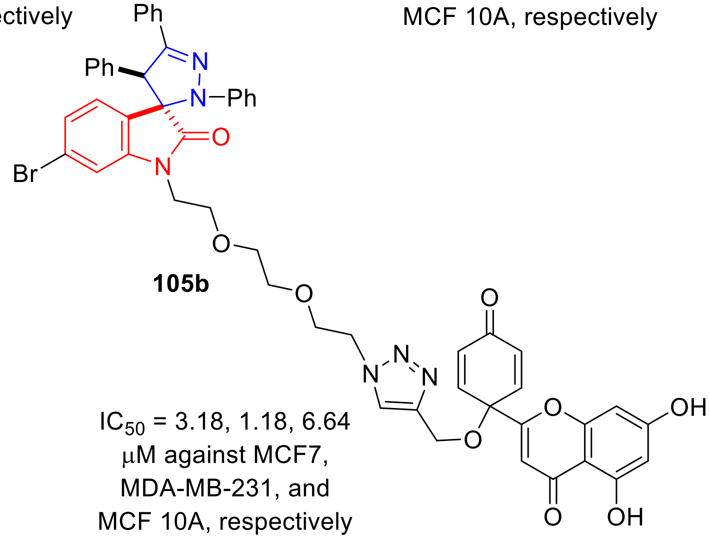

105b

IC_{50} = 3.18, 1.18, 6.64 µM against MCF7, MDA-MB-231, and MCF 10A, respectively

Fig. S18. Antiproliferation properties of spirooxindole-pyrazolines linked to triazolyl heterocycle **104**/**105**.

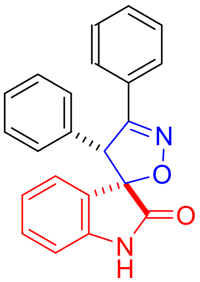

107a

GI_{50} = >100 μM against HepG2

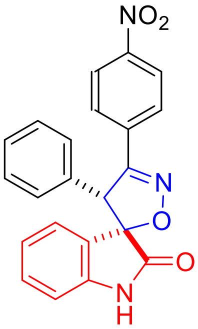

107b

GI_{50} = 52.14 ± 1.09 μM against HepG2

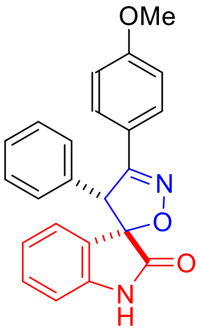

107c

GI_{50} = 77.77 ± 1.05 μM against HepG2

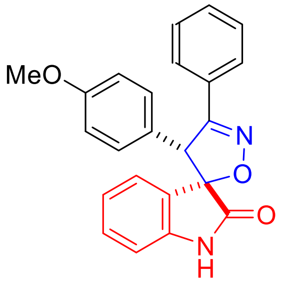

107d

GI_{50} = >100 μM against HepG2

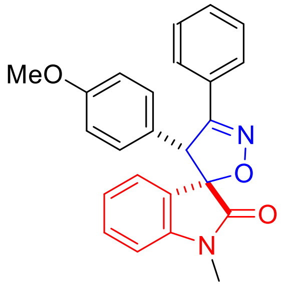

107e

GI_{50} = >100 μM against HepG2

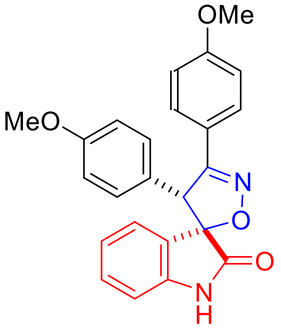

107f

GI_{50} = >100 μM against HepG2

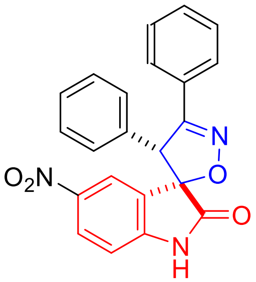

107g

GI_{50} = 83.59 ± 1.04 μM against HepG2

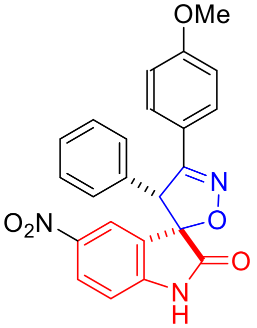

107h

GI_{50} = 53.10 ± 1.14 μM against HepG2

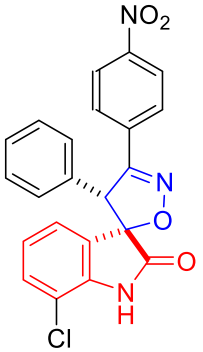

107i

GI_{50} = 55.48 ± 1.11 μM against HepG2

Fig. S19. Antiproliferation properties of spirooxindole-isoxazolines **107** and nutlin-3.

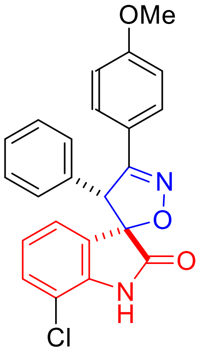

107j

GI_{50} = 55.79 ± 1.11 μM against HepG2

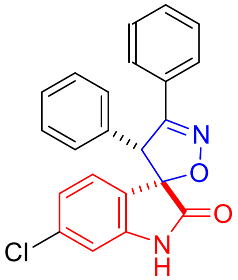

107k

GI_{50} = 37.07 ± 1.08, 39.80 ± 1.10, 48.22 ± 1.13, 48.86 ± 1.08 μM against HepG2, HCT116 ($p53^{+/+}$), HCT116 ($p53^{-/-}$), and SW620, respectively

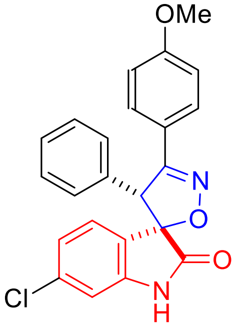

107l

GI_{50} = 38.14 ± 1.08, 34.72 ± 1.14, 40.14 ± 1.17, 53.20 ± 1.10 μM against HepG2, HCT116 ($p53^{+/+}$), HCT116 ($p53^{-/-}$), and SW620, respectively

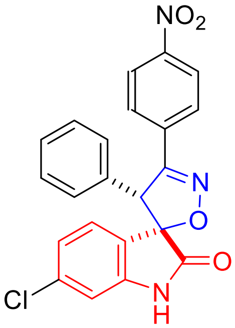

107m

GI_{50} = 33.84 ± 1.11, 32.37 ± 1.12, 38.97 ± 1.14, 33.52 ± 1.07 μM against HepG2, HCT116 ($p53^{+/+}$), HCT116 ($p53^{-/-}$), and SW620, respectively

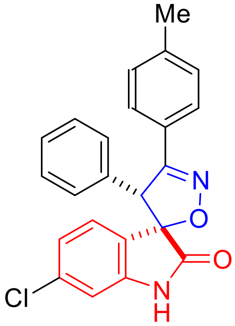

107n

GI_{50} = 29.11 ± 1.09, 26.56 ± 1.07, 30.64 ± 1.12, 31.56 ± 1.05 μM against HepG2, HCT116 ($p53^{+/+}$), HCT116 ($p53^{-/-}$), and SW620, respectively

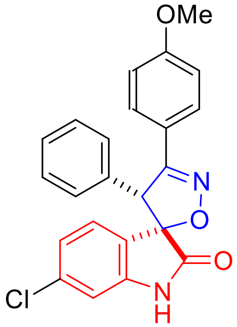

107o

GI_{50} = 35.70 ± 1.07, 30.51 ± 1.08, 35.56 ± 1.10, 36.74 ± 1.04 μM against HepG2, HCT116 ($p53^{+/+}$), HCT116 ($p53^{-/-}$), and SW620, respectively

Fig. S19 (continued)**.** Antiproliferation properties of spirooxindole-isoxazolines **107** and nutlin-3.

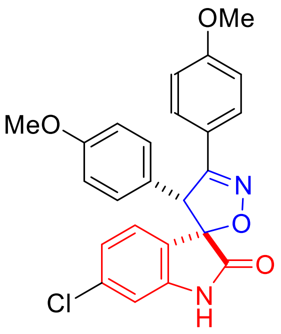

107p

GI_{50} = >100 μM against HepG2

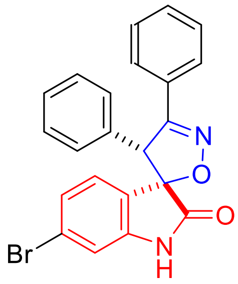

107q

GI_{50} = 32.84 ± 1.07, 35.01 ± 1.07, 40.55 ± 1.11, 39.65 ± 1.07 μM against HepG2, HCT116 ($p53^{+/+}$), HCT116 ($p53^{-/-}$), and SW620, respectively

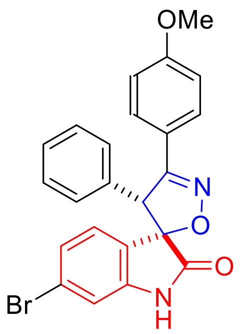

107r

GI_{50} = 31.69 ± 1.10, 33.38 ± 1.13, 39.03 ± 1.18, 40.36 ± 1.09 μM against HepG2, HCT116 ($p53^{+/+}$), HCT116 ($p53^{-/-}$), and SW620, respectively

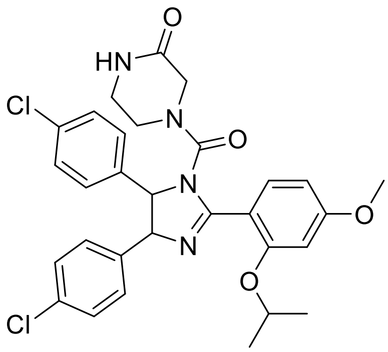

Nutlin-3

GI_{50} = 51.31 ± 1.04, 39.65 ± 1.12, 52.34 ± 1.15, 57.04 ± 1.04 μM against HepG2, HCT116 ($p53^{+/+}$), HCT116 ($p53^{-/-}$), and SW620, respectively

Fig. S19 (continued). Antiproliferation properties of spirooxindole-isoxazolines **107** and nutlin-3.

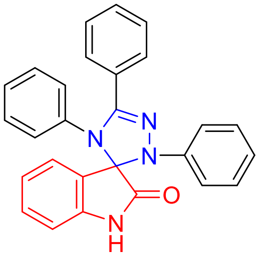

108a

IC_{50} = 36.2 ± 3.3, 23.4 ± 4.3 μM against MCF7, and MDA-MB-231, respectively

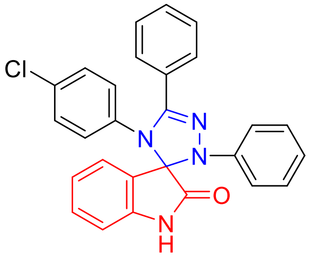

108b

IC_{50} = 24.1 ± 4.8, 10.7 ± 1.0, 15.0 ± 1.5, 18.5 ± 1.2, >100 μM against MCF7, MDA-MB-231, HCT116 ($p53^{+/+}$), HCT116 ($p53^{-/-}$), and HEK 293T, respectively

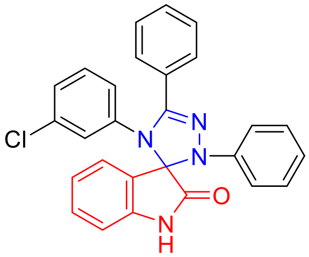

108c

IC_{50} = 13.7 ± 0.4, 12.5 ± 1.1, 19.9 ± 1.4, 22.1 ± 2.0, 25.7 ± 5.6 μM against MCF7, MDA-MB-231, HCT116 ($p53^{+/+}$), HCT116 ($p53^{-/-}$), and HEK 293T, respectively

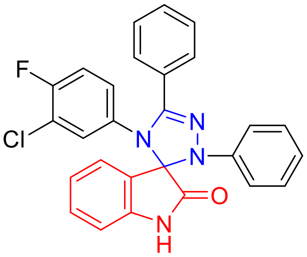

108d

IC_{50} = 19.5 ± 0.7, 32.3 ± 4.0 μM against MCF7, and MDA-MB-231, respectively

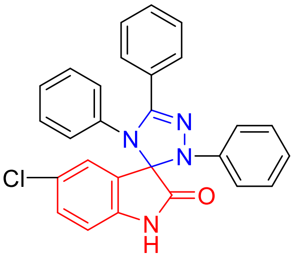

108e

IC_{50} = 19.1 ± 0.8, 11.7 ± 0.6, 21.6 ± 0.6, 22.1 ± 3.2, 32.6 ± 9.3 μM against MCF7, MDA-MB-231, HCT116 ($p53^{+/+}$), HCT116 ($p53^{-/-}$), and HEK 293T, respectively

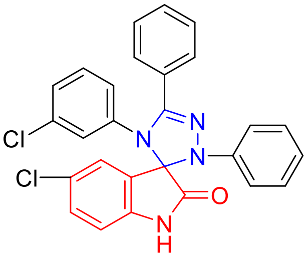

108f

IC_{50} = 9.8 ± 1.5, 8.9 ± 1.9, 16.3 ± 0.2, 17.9 ± 0.6, >100 μM against MCF7, MDA-MB-231, HCT116 ($p53^{+/+}$), HCT116 ($p53^{-/-}$), and HEK 293T, respectively

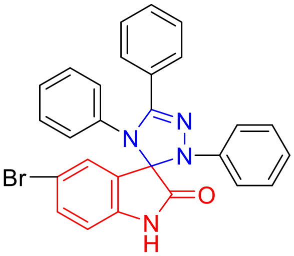

108g

IC_{50} = 23.0 ± 1.7, 6.7 ± 0.4, 22.2 ± 0.5, 22.5 ± 4.4, 56.3 ± 11.3 μM against MCF7, MDA-MB-231, HCT116 ($p53^{+/+}$), HCT116 ($p53^{-/-}$), and HEK 293T, respectively

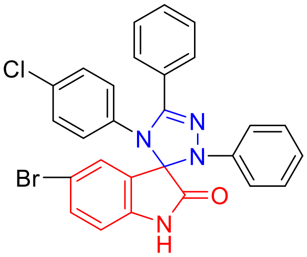

108h

IC_{50} = 16.8 ± 1.0, 5.2 ± 1.0, 16.3 ± 1.2, 18.2 ± 0.9, 21.6 ± 6.8 μM against MCF7, MDA-MB-231, HCT116 ($p53^{+/+}$), HCT116 ($p53^{-/-}$), and HEK 293T, respectively

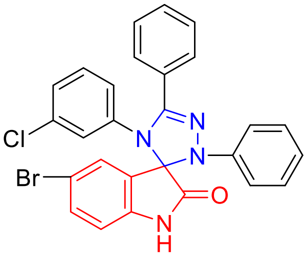

108i

IC_{50} = 21.4 ± 2.2, 3.5 ± 1.0, 15.7 ± 1.4, 17.8 ± 2.0, 21.6 ± 6.8 μM against MCF7, MDA-MB-231, HCT116 ($p53^{+/+}$), HCT116 ($p53^{-/-}$), and HEK 293T, respectively

Fig. S20. Antiproliferation properties of spirooxindole-triazoles **108** and nutlin-3a.

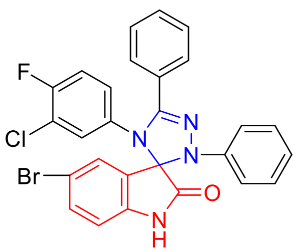

108j

IC_{50} = 11.0 ± 2.4, 4.6 ± 0.3, 13.5 ± 1.0, 15.9 ± 1.3, 16.6 ± 3.7 μM against MCF7, MDA-MB-231, HCT116 ($p53^{+/+}$), HCT116 ($p53^{-/-}$), and HEK 293T, respectively

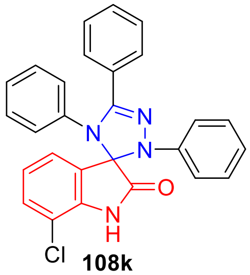

108k

IC_{50} = 10.8 ± 0.9, 8.9 ± 0.6, 23.7 ± 0.6, 20.1 ± 1.7, >100 μM against MCF7, MDA-MB-231, HCT116 ($p53^{+/+}$), HCT116 ($p53^{-/-}$), and HEK 293T, respectively

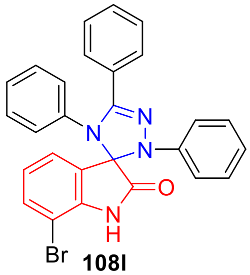

108l

IC_{50} = 21.8 ± 1.6, 23.7 ± 4.5, μM against MCF7, and MDA-MB-231, respectively

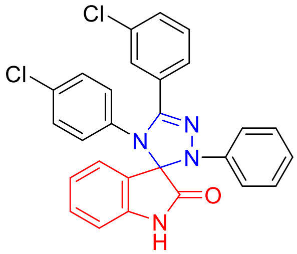

108m

IC_{50} = 13.1 ± 2.4, 11.6 ± 1.3, 17.4 ± 2.4, 16.1 ± 1.6, 20.5 ± 5.3 μM against MCF7, MDA-MB-231, HCT116 ($p53^{+/+}$), HCT116 ($p53^{-/-}$), and HEK 293T, respectively

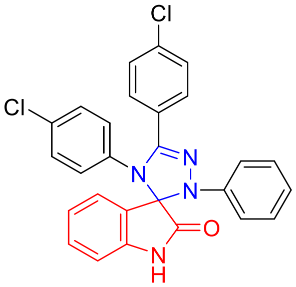

108n

IC_{50} = 10.9 ± 2.0, 8.2 ± 0.8, 16.8 ± 2.2, 18.5 ± 1.6, 22.7 ± 5.3 μM against MCF7, MDA-MB-231, HCT116 ($p53^{+/+}$), HCT116 ($p53^{-/-}$), and HEK 293T, respectively

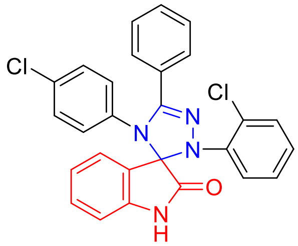

108o

IC_{50} = 28.4 ± 5.0, 24.8 ± 0.8 μM against MCF7, and MDA-MB-231, respectively

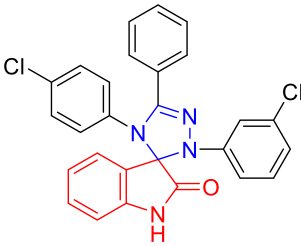

108p

IC_{50} = 14.2 ± 2.4, 15.7 ± 2.4, 19.8 ± 1.4, 18.5 ± 0.9, 15.4 ± 2.6 μM against MCF7, MDA-MB-231, HCT116 ($p53^{+/+}$), HCT116 ($p53^{-/-}$), and HEK 293T, respectively

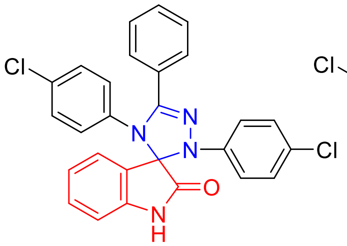

108q

IC_{50} = 10.1 ± 0.5, 9.0 ± 1.2, 18.5 ± 0.9, 18.7 ± 0.5, 15.8 ± 4.4 μM against MCF7, MDA-MB-231, HCT116 ($p53^{+/+}$), HCT116 ($p53^{-/-}$), and HEK 293T, respectively

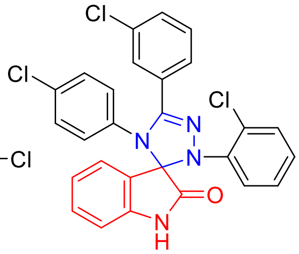

108r

IC_{50} = 13.1 ± 0.5, 14.5 ± 0.5, 25.3 ± 1.3, 34.6 ± 2.9, 35.0 ± 12.2 μM against MCF7, MDA-MB-231, HCT116 ($p53^{+/+}$), HCT116 ($p53^{-/-}$), and HEK 293T, respectively

Fig. S20 (continued)**.** Antiproliferation properties of spirooxindole-triazoles **108** and nutlin-3a.

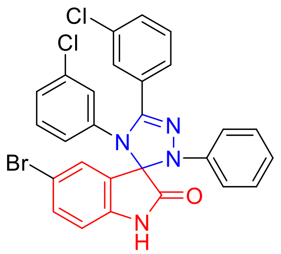

108s

IC_{50} = 8.0 ± 1.4, 10.2 ± 1.3, 12.6 ± 1.2, 13.0 ± 1.4, 12.5 ± 1.1 μM against MCF7, MDA-MB-231, HCT116 ($p53^{+/+}$), HCT116 ($p53^{-/-}$), and HEK 293T, respectively

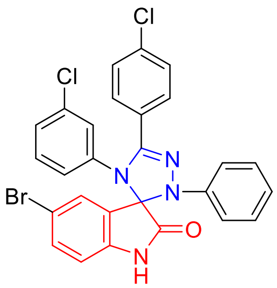

108t

IC_{50} = 10.1 ± 0.9, 14.6 ± 2.1, 10.8 ± 1.2, 10.8 ± 0.9, 20.3 ± 6.5 μM against MCF7, MDA-MB-231, HCT116 ($p53^{+/+}$), HCT116 ($p53^{-/-}$), and HEK 293T, respectively

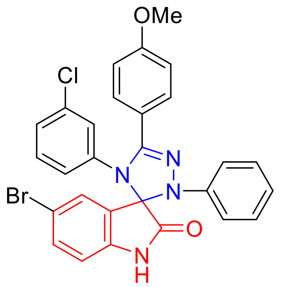

108u

IC_{50} = 12.3 ± 1.5, 14.1 ± 1.1, 22.2 ± 1.4, 25.8 ± 1.2, 25.9 ± 5.8 μM against MCF7, MDA-MB-231, HCT116 ($p53^{+/+}$), HCT116 ($p53^{-/-}$), and HEK 293T, respectively

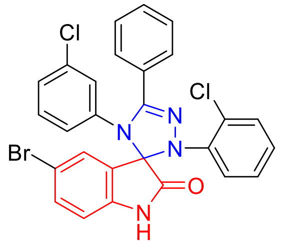

108v

IC_{50} = 10.2 ± 0.8, 11.7 ± 1.7, 21.5 ± 2.1, 31.6 ± 1.5, 20.9 ± 3.0 μM against MCF7, MDA-MB-231, HCT116 ($p53^{+/+}$), HCT116 ($p53^{-/-}$), and HEK 293T, respectively

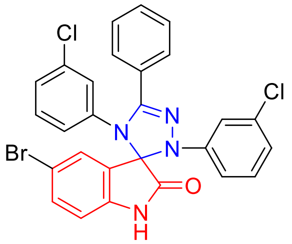

108w

IC_{50} = 9.8 ± 0.9, 10.7 ± 3.1, 12.0 ± 1.1, 13.1 ± 1.4, 16.9 ± 3.4 μM against MCF7, MDA-MB-231, HCT116 ($p53^{+/+}$), HCT116 ($p53^{-/-}$), and HEK 293T, respectively

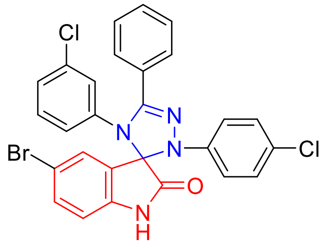

108x

IC_{50} = 11.4 ± 0.9, 15.0 ± 4.4, 15.0 ± 1.3, 17.2 ± 1.8, 16.2 ± 2.4 μM against MCF7, MDA-MB-231, HCT116 ($p53^{+/+}$), HCT116 ($p53^{-/-}$), and HEK 293T, respectively

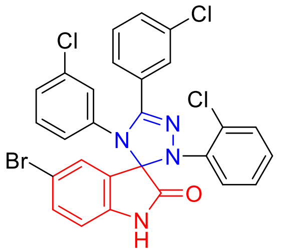

108y

IC_{50} = 9.5 ± 3.1, 7.6 ± 2.4, 13.9 ± 1.1, 17.7 ± 1.3, 21.4 ± 7.4 μM against MCF7, MDA-MB-231, HCT116 ($p53^{+/+}$), HCT116 ($p53^{-/-}$), and HEK 293T, respectively

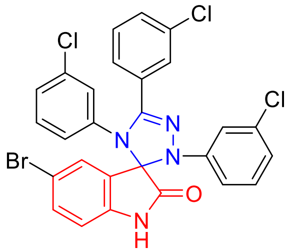

108z

IC_{50} = 6.9 ± 0.7, 7.0 ± 1.9, 10.2 ± 1.7, 9.0 ± 0.8, 9.0 ± 1.7 μM against MCF7, MDA-MB-231, HCT116 ($p53^{+/+}$), HCT116 ($p53^{-/-}$), and HEK 293T, respectively

Fig. S20 (continued)**.** Antiproliferation properties of spirooxindole-triazoles **108** and nutlin-3a.

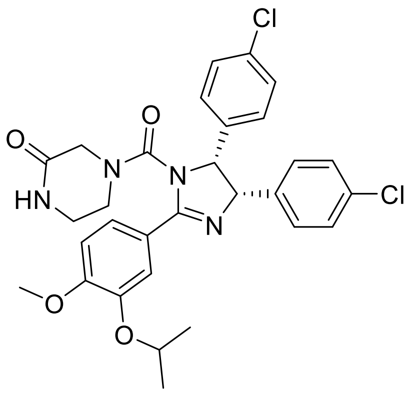

Nutlin-3a

IC_{50} = 4.0 ± 1.2, 47.8 ± 1.9 μM against HCT116 ($p53^{+/+}$), and HCT116 ($p53^{-/-}$), respectively

Fig. S20 (continued)**.** Antiproliferation properties of spirooxindole-triazoles **108** and nutlin-3a.

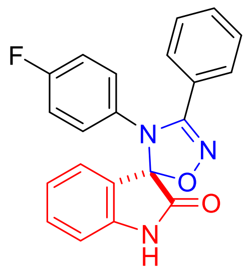

110a

GI_{50} = 99.6 ± 0.8 μM against HCT116 ($p53^{+/+}$)

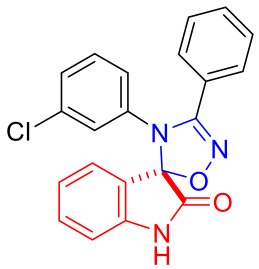

110b

GI_{50} = 70.1 ± 3.2 μM against HCT116 ($p53^{+/+}$)

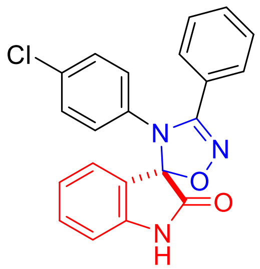

110c

GI_{50} = 54.5 ± 0.1 μM against HCT116 ($p53^{+/+}$)

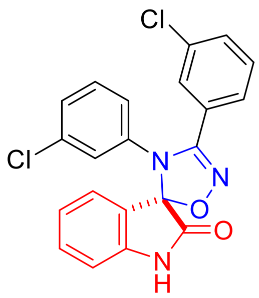

110d

GI_{50} = 32.6 ± 0.4 μM against HCT116 ($p53^{+/+}$)

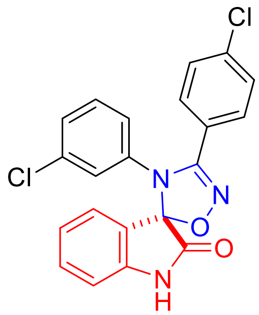

110e

GI_{50} = 33.7 ± 0.1 μM against HCT116 ($p53^{+/+}$)

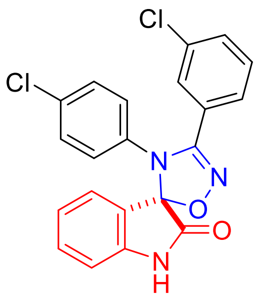

110f

GI_{50} = 38.4 ± 2.4 μM against HCT116 ($p53^{+/+}$)

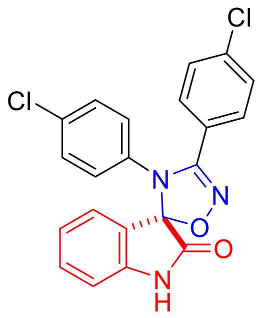

110g

GI_{50} = 31.2 ± 2.5 μM against HCT116 ($p53^{+/+}$)

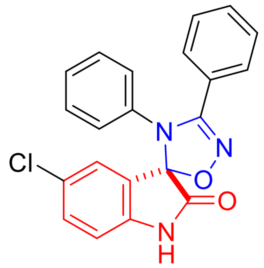

110h

GI_{50} = 39.1 ± 2.0 μM against HCT116 ($p53^{+/+}$)

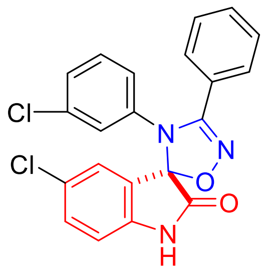

110i

GI_{50} = 26.4 ± 0.3, 41.6 ± 2.0 μM against HCT116 ($p53^{+/+}$), and HCT116 ($p53^{-/-}$), respectively

Fig. S21. Antiproliferation properties of spirooxindole-oxadiazoles **110** and nutlin-3a.

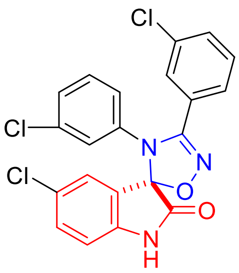

110j

GI$_{50}$ = 4.6 ± 0.2, 6.8 ± 0.7, 3.3 ± 0.3, 3.9 ± 0.4 µM against HCT116 (p53$^{+/+}$), HCT116 (p53$^{-/-}$), HepG2, and SW620 respectively

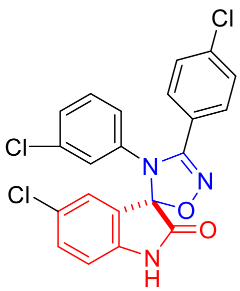

110k

GI$_{50}$ = 25.6 ± 1.0, 29.0 ± 0.6 µM against HCT116 (p53$^{+/+}$), and HCT116 (p53$^{-/-}$), respectively

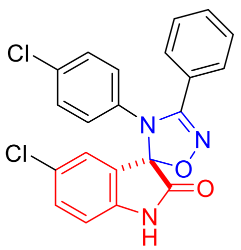

110l

GI$_{50}$ = 27.1 ± 2.5, 27.0 ± 2.7 µM against HCT116 (p53$^{+/+}$), and HCT116 (p53$^{-/-}$), respectively

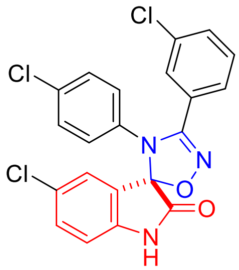

110m

GI$_{50}$ = 21.3 ± 1.0, 21.8 ± 1.4 µM against HCT116 (p53$^{+/+}$), and HCT116 (p53$^{-/-}$), respectively

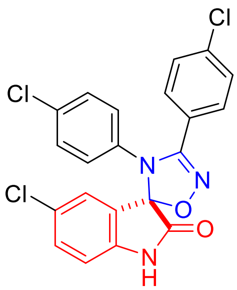

110n

GI$_{50}$ = 17.6 ± 0.5, 18.0 ± 0.8 µM against HCT116 (p53$^{+/+}$), and HCT116 (p53$^{-/-}$), respectively

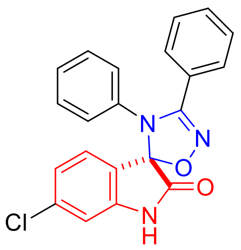

110o

GI$_{50}$ = 28.4 ± 4.0, 35.5 ± 6.7 µM against HCT116 (p53$^{+/+}$), and HCT116 (p53$^{-/-}$), respectively

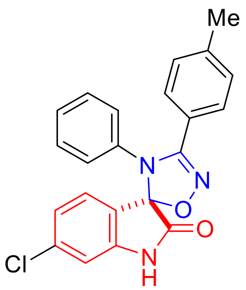

110p

GI$_{50}$ = 50.6 ± 4.4 µM against HCT116 (p53$^{+/+}$), respectively

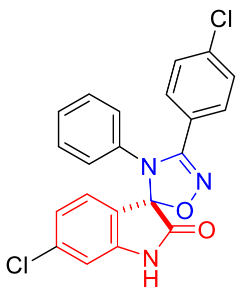

110q

GI$_{50}$ = 26.5 ± 0.1, 30.6 ± 1.3 µM against HCT116 (p53$^{+/+}$), and HCT116 (p53$^{-/-}$), respectively

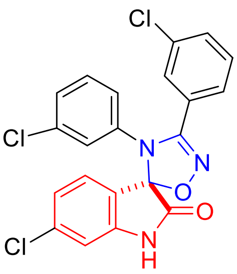

110r

GI$_{50}$ = 6.6 ± 0.0, 7.8 ± 0.8, 4.4 ± 0.4, 6.7 ± 0.8 µM against HCT116 (p53$^{+/+}$), HCT116 (p53$^{-/-}$), HepG2, and SW620 respectively

Fig. S21 (continued)**.** Antiproliferation properties of spirooxindole-oxadiazoles **110** and nutlin-3a.

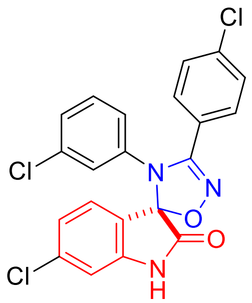

110s

GI_{50} = 20.2 ± 1.0 μM against HCT116 ($p53^{+/+}$), respectively

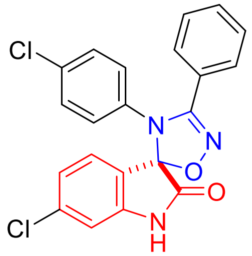

110t

GI_{50} = 31.8 ± 1.9 μM against HCT116 ($p53^{+/+}$), respectively

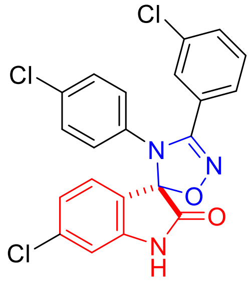

110u

GI_{50} = 28.7 ± 2.1 μM against HCT116 ($p53^{+/+}$), respectively

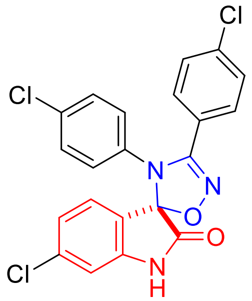

110v

GI_{50} = 13.2 ± 0.4 μM against HCT116 ($p53^{+/+}$), respectively

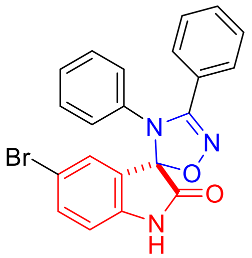

110w

GI_{50} = 13.5 ± 4.5 μM against HCT116 ($p53^{+/+}$), respectively

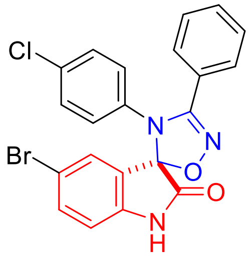

110x

GI_{50} = 21.9 ± 2.4, 25.8 ± 0.6 μM against HCT116 ($p53^{+/+}$), and HCT116 ($p53^{-/-}$), respectively

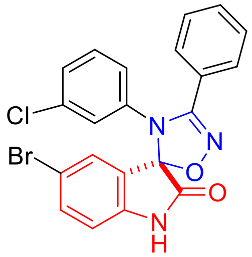

110y

GI_{50} = 4.7 ± 0.2, 7.5 ± 1.3, 3.5 ± 0.4, 4.2 ± 0.3 μM against HCT116 ($p53^{+/+}$), HCT116 ($p53^{-/-}$), HepG2, and SW620 respectively

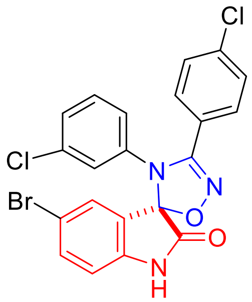

110z

GI_{50} = 7.7 ± 0.1, 12.3 ± 1.4, 4.1 ± 0.4, 8.0 ± 0.4 μM against HCT116 ($p53^{+/+}$), HCT116 ($p53^{-/-}$), HepG2, and SW620 respectively

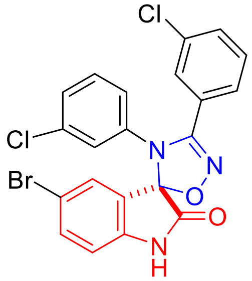

110aa

GI_{50} = 2.0 ± 0.0, 2.9 ± 0.2, 2.1 ± 0.0, 2.1 ± 0.3 μM against HCT116 ($p53^{+/+}$), HCT116 ($p53^{-/-}$), HepG2, and SW620 respectively

Fig. S21 (continued)**.** Antiproliferation properties of spirooxindole-oxadiazoles **110** and nutlin-3a.

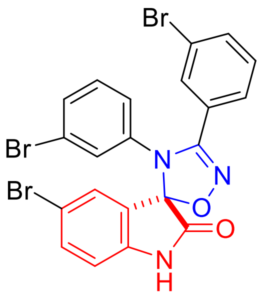

110ab

GI_{50} = 3.2 ± 0.1, 6.0 ± 0.6, 2.2 ± 0.2, 3.7 ± 0.4 μM against HCT116 ($p53^{+/+}$), HCT116 ($p53^{-/-}$), HepG2, and SW620 respectively

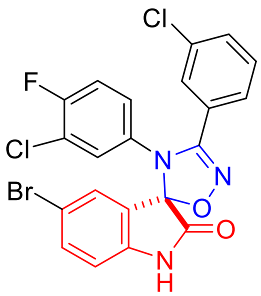

110ac

GI_{50} = 6.6 ± 0.2, 7.2 ± 0.5, 4.0 ± 0.4, 6.7 ± 0.2 μM against HCT116 ($p53^{+/+}$), HCT116 ($p53^{-/-}$), HepG2, and SW620 respectively

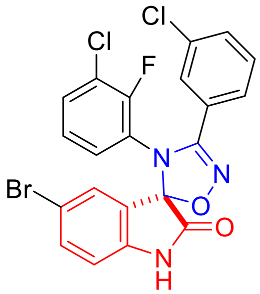

110ad

GI_{50} = 1.7 ± 0.1, 2.0 ± 0.4, 1.2 ± 0.2, 2.0 ± 0.2 μM against HCT116 ($p53^{+/+}$), HCT116 ($p53^{-/-}$), HepG2, and SW620 respectively

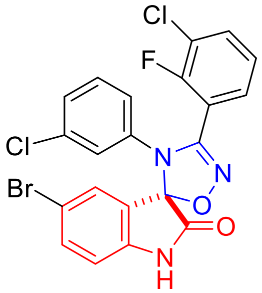

110ae

GI_{50} = 3.2 ± 0.3, 4.4 ± 0.8, 2.5 ± 0.06, 3.5 ± 0.04 μM against HCT116 ($p53^{+/+}$), HCT116 ($p53^{-/-}$), HepG2, and SW620 respectively

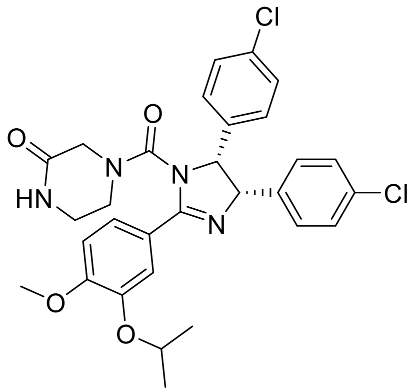

Nutlin-3a

GI_{50} = 4.0 ± 1.2, 47.8 ± 1.9 μM against HCT116 ($p53^{+/+}$), and HCT116 ($p53^{-/-}$), respectively

Fig. S21 (continued)**.** Antiproliferation properties of spirooxindole-oxadiazoles **110** and nutlin-3a,

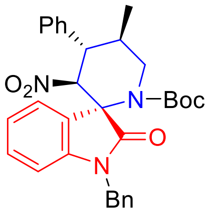

116a

% Inhibition at 20 μM = 32.26, 34.78 against MCF7, ZR-75-1 (wild-type p53), 15.33 against BT-474 (mutated-type p53), and 28.00 against MDM2

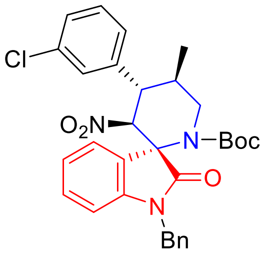

116b

% Inhibition at 20 μM = 31.75, 28.94 against MCF7, ZR-75-1 (wild-type p53), 8.67 against BT-474 (mutated-type p53), and 20.40 against MDM2

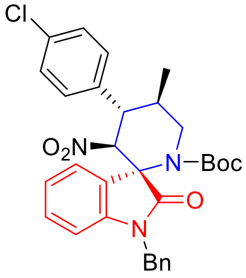

116c

% Inhibition at 20 μM = 34.94, 29.36 against MCF7, ZR-75-1 (wild-type p53), 9.82 against BT-474 (mutated-type p53), and 16.38 against MDM2

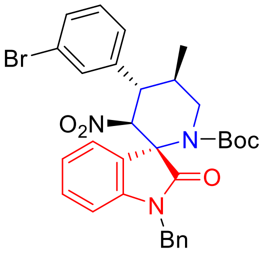

116d

% Inhibition at 20 μM = 25.71, 23.01 against MCF7, ZR-75-1 (wild-type p53), 10.89 against BT-474 (mutated-type p53), and 21.14 against MDM2

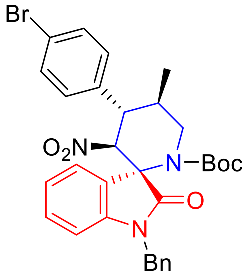

116e

% Inhibition at 20 μM = 31.73, 25.93 against MCF7, ZR-75-1 (wild-type p53), 9.47 against BT-474 (mutated-type p53), and 21.92 against MDM2

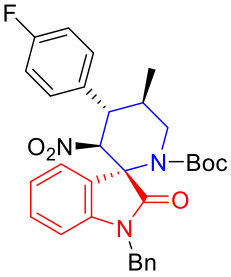

116f

% Inhibition at 20 μM = 26.66, 21.30 against MCF7, ZR-75-1 (wild-type p53), 11.63 against BT-474 (mutated-type p53), and 16.29 against MDM2

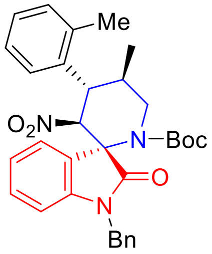

116g

% Inhibition at 20 μM = 33.17, 31.13 against MCF7, ZR-75-1 (wild-type p53), 14.25 against BT-474 (mutated-type p53), and 21.66 against MDM2

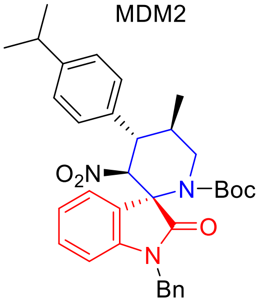

116h

% Inhibition at 20 μM = 33.66, 23.82 against MCF7, ZR-75-1 (wild-type p53), 14.00 against BT-474 (mutated-type p53), and 24.49 against MDM2

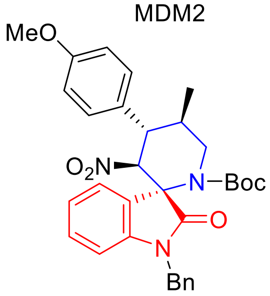

116i

% Inhibition at 20 μM = 36.94, 22.72 against MCF7, ZR-75-1 (wild-type p53), 17.31 against BT-474 (mutated-type p53), and 28.52 against MDM2

Fig. S22. Antiproliferation properties and MDM2 inhibitory effect of spirooxindole-piperidines **116–119**.

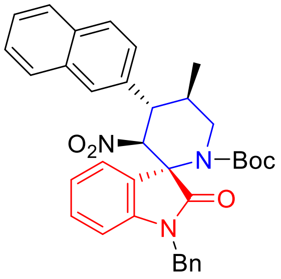

116j

% Inhibition at 20 μM = 26.36, 25.87 against MCF7, ZR-75-1 (wild-type p53), 19.18 against BT-474 (mutated-type p53), and 13.93 against MDM2

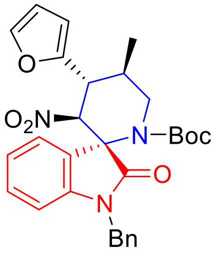

116k

% Inhibition at 20 μM = 25.14, 24.91 against MCF7, ZR-75-1 (wild-type p53), 11.62 against BT-474 (mutated-type p53), and 28.25 against MDM2

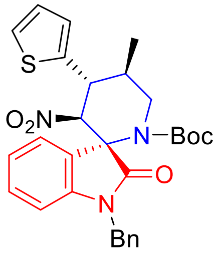

116l

% Inhibition at 20 μM = 26.78, 34.15 against MCF7, ZR-75-1 (wild-type p53), 9.40 against BT-474 (mutated-type p53), and 11.58 against MDM2

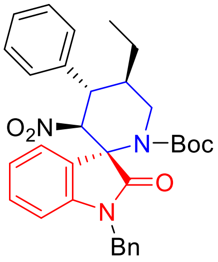

116m

% Inhibition at 20 μM = 33.86, 22.98 against MCF7, ZR-75-1 (wild-type p53), 18.24 against BT-474 (mutated-type p53), and 22.85 against MDM2

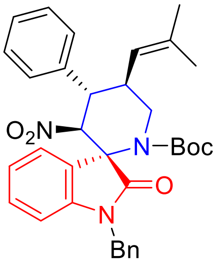

116n

% Inhibition at 20 μM = 34.81, 28.09 against MCF7, ZR-75-1 (wild-type p53), 5.65 against BT-474 (mutated-type p53), and 23.03 against MDM2

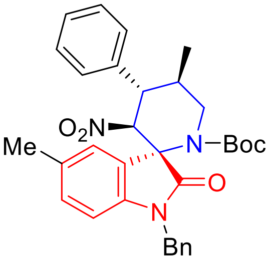

116o

% Inhibition at 20 μM = 26.27, 20.57 against MCF7, ZR-75-1 (wild-type p53), 6.14 against BT-474 (mutated-type p53), and 23.59 against MDM2

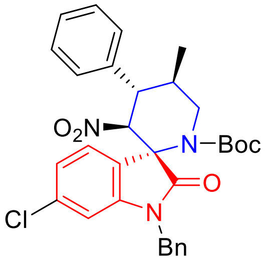

116p

% Inhibition at 20 μM = 29.78, 22.75 against MCF7, ZR-75-1 (wild-type p53), 5.53 against BT-474 (mutated-type p53), and 19.05 against MDM2

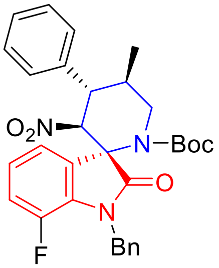

116q

% Inhibition at 20 μM = 26.50, 34.42 against MCF7, ZR-75-1 (wild-type p53), 15.88 against BT-474 (mutated-type p53), and 17.71 against MDM2

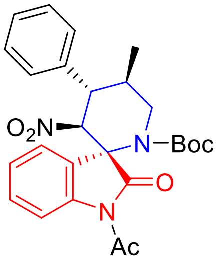

116r

% Inhibition at 20 μM = 28.74, 22.91 against MCF7, ZR-75-1 (wild-type p53), 19.88 against BT-474 (mutated-type p53), and 17.34 against MDM2

Fig. S22 (continued)**.** Antiproliferation properties and MDM2 inhibitory effect of spirooxindole-piperidines **116–119**.

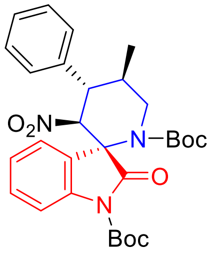

116s

% Inhibition at 20 µM = 28.27, 23.26 against MCF7, ZR-75-1 (wild-type p53), 16.32 against BT-474 (mutated-type p53), and 13.95 against MDM2

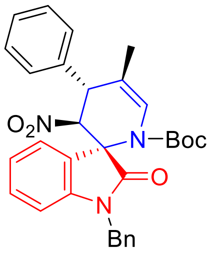

117a

% Inhibition at 20 µM = 32,13, 24.77 against MCF7, ZR-75-1 (wild-type p53), 19.97 against BT-474 (mutated-type p53), and 24.39 against MDM2

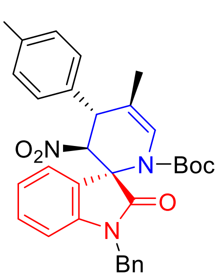

117b

% Inhibition at 20 µM = 34.13, 20.05 against MCF7, ZR-75-1 (wild-type p53), 6.91 against BT-474 (mutated-type p53), and 26.90 against MDM2

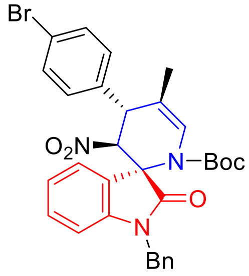

117c

% Inhibition at 20 µM = 25.52, 25.73 against MCF7, ZR-75-1 (wild-type p53), 9.37 against BT-474 (mutated-type p53), and 22.29 against MDM2

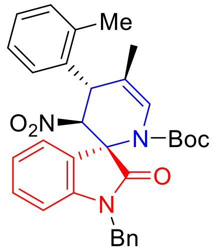

117d

% Inhibition at 20 µM = 29.97, 33.21 against MCF7, ZR-75-1 (wild-type p53), 10.33 against BT-474 (mutated-type p53), and 15.01 against MDM2

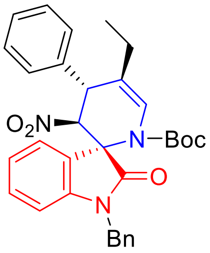

117e

% Inhibition at 20 µM = 27.33, 33.87 against MCF7, ZR-75-1 (wild-type p53), 10.69 against BT-474 (mutated-type p53), and 29.30 against MDM2

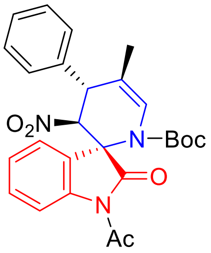

117f

% Inhibition at 20 µM = 41.83, 39.94 against MCF7, ZR-75-1 (wild-type p53), 19.06 against BT-474 (mutated-type p53), and 37.92 against MDM2

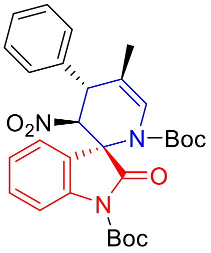

117g

% Inhibition at 20 µM = 30.88, 27.97 against MCF7, ZR-75-1 (wild-type p53), 17.71 against BT-474 (mutated-type p53), and 19.73 against MDM2

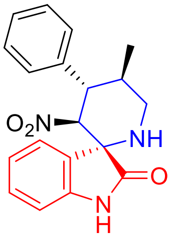

118a

% Inhibition at 20 µM = 62.26, 45.92 against MCF7, ZR-75-1 (wild-type p53), 24.52 against BT-474 (mutated-type p53), and 49.64 against MDM2

Fig. S22 (continued)**.** Antiproliferation properties and MDM2 inhibitory effect of spirooxindole-piperidines **116–119**.

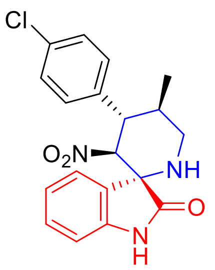

118b

% Inhibition at 20 µM = 63.14, 62.52 against MCF7, ZR-75-1 (wild-type p53), 32.00 against BT-474 (mutated-type p53), and 58.51 against MDM2

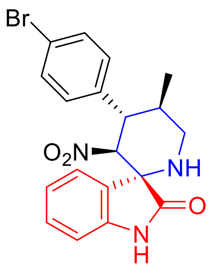

118c

% Inhibition at 20 µM = 54.28, 52.27 against MCF7, ZR-75-1 (wild-type p53), 37.87 against BT-474 (mutated-type p53), and 53.86 against MDM2

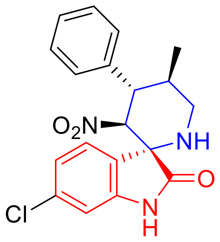

118d

% Inhibition at 20 µM = 56.44, 51.39 against MCF7, ZR-75-1 (wild-type p53), 34.13 against BT-474 (mutated-type p53), and 56.87 against MDM2

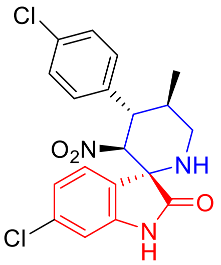

118e

% Inhibition at 20 µM = 72.09, 65.55 against MCF7, ZR-75-1 (wild-type p53), 28.06 against BT-474 (mutated-type p53), and 62.97 against MDM2

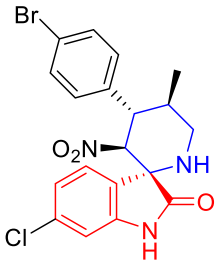

118f

% Inhibition at 20 µM = 64.28, 59.44 against MCF7, ZR-75-1 (wild-type p53), 39.52 against BT-474 (mutated-type p53), and 55.40 against MDM2

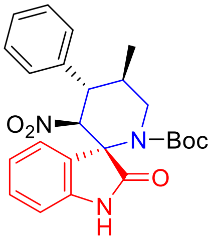

119a

% Inhibition at 20 µM = 77.08, 61.57 against MCF7, ZR-75-1 (wild-type p53), 45.78 against BT-474 (mutated-type p53), and 67.27 against MDM2

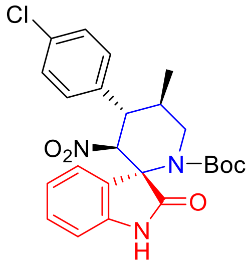

119b

% Inhibition at 20 µM = 80.20, 76.77 against MCF7, ZR-75-1 (wild-type p53), 43.22 against BT-474 (mutated-type p53), and 74.98 against MDM2

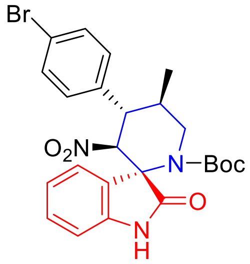

119c

% Inhibition at 20 µM = 71.50, 64.84 against MCF7, ZR-75-1 (wild-type p53), 39.11 against BT-474 (mutated-type p53), and 70.97 against MDM2

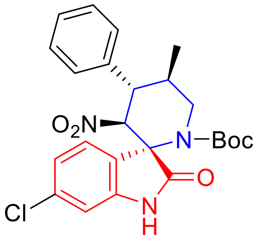

119d

% Inhibition at 20 µM = 85.36, 75.25 against MCF7, ZR-75-1 (wild-type p53), 43.33 against BT-474 (mutated-type p53), and 74.38 against MDM2

Fig. S22 (continued)**.** Antiproliferation properties and MDM2 inhibitory effect of spirooxindole-piperidines **116–119**.

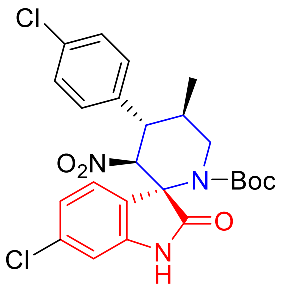

119e

% Inhibition at 20 μM = 93.03, 82.28 against MCF7, ZR-75-1 (wild-type p53), 50.00 against BT-474 (mutated-type p53), and 84.86 against MDM2

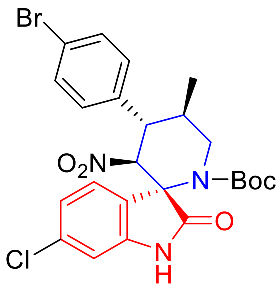

119f

% Inhibition at 20 μM = 91.64, 80.63 against MCF7, ZR-75-1 (wild-type p53), 33.40 against BT-474 (mutated-type p53), and 78.61 against MDM2

Fig. S22 (continued)**.** Antiproliferation properties and MDM2 inhibitory effect of spirooxindole-piperidines **116**–**119**.

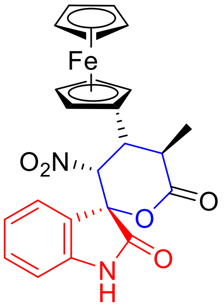

123a

% Inhibition at 1.0 μM
= 50.14, 55.14, 36.80
against MCF-7,
ZR-75-1, MDA-MB-231;
and 46 against
MDM2, respectivrly

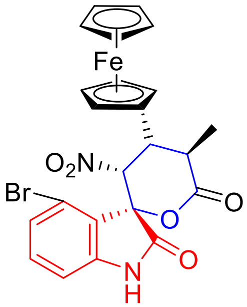

123b

% Inhibition at 1.0 μM
= 39.78, 43.78, 29.64
against MCF-7,
ZR-75-1, MDA-MB-231;
and 39 against
MDM2, respectivrly

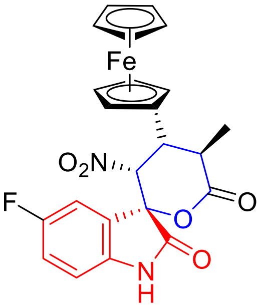

123c

% Inhibition at 1.0 μM
= 54.45, 62.03, 37.46
against MCF-7,
ZR-75-1, MDA-MB-231;
and 55 against
MDM2 ; *Ki* = 0.92 ±
0.07 μM against
MDM2, respectivrly

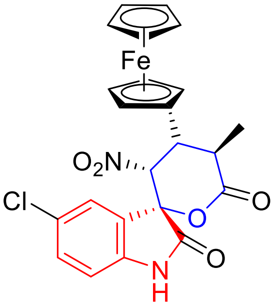

123d

% Inhibition at 1.0 μM
= 58.28, 67.02, 46.65
against MCF-7,
ZR-75-1, MDA-MB-231;
and 62 against MDM2;
Ki = 0.53 ± 0.03 μM against
MDM2; IC_{50} = 0.88 ±
0.08, 0.67 ± 0.06, 4.63
± 0.38, 6.27 ± 0.49,
3.56 ± 0.47, 6.59 ± 0.81,
5.61 ± 0.36 μM against
MCF-7, ZR-75-1, BT-474,
HCC1937, MDA-MB-231,
MDA-MB-435, SKBR-3,
respectivrly

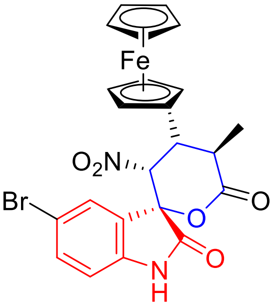

123e

% Inhibition at 1.0 μM
= 57.23, 53.29, 42.48
against MCF-7,
ZR-75-1, MDA-MB-231;
and 59 against MDM2;
Ki = 0.78 ± 0.08 μM,
respectivrly

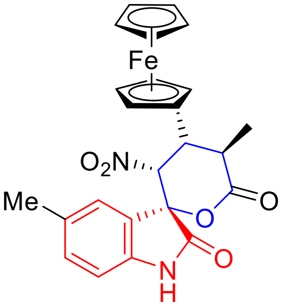

123f

% Inhibition at 1.0 μM
= 54.06, 50.27, 34.17
against MCF-7,
ZR-75-1, MDA-MB-231;
and 51 against MDM2;
Ki = 0.92 ± 0.07 μM,
respectivrly

Fig. S23. Antiproliferation and MDM2 inhibitory properties of spirooxindole-pyrans **123–125**.

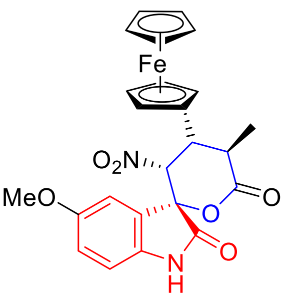

123g

% Inhibition at 1.0 μM = 58.30, 60.22, 33.92 against MCF-7, ZR-75-1, MDA-MB-231; and 53 against MDM2; *Ki* = 0.87 ± 0.11 μM, respectivrly

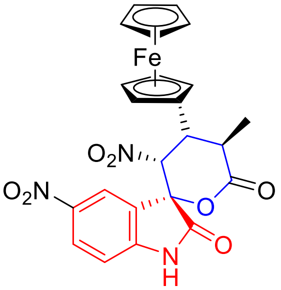

123h

% Inhibition at 1.0 μM = 43.87, 48.25, 33.21 against MCF-7, ZR-75-1, MDA-MB-231; and 41 against MDM2; *Ki* = 3.48 ± 0.32 μM, respectivrly

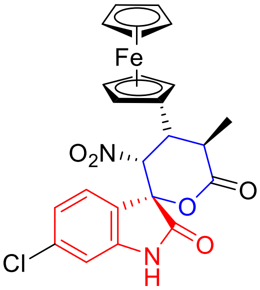

123i

% Inhibition at 1.0 μM = 53.94, 59.33, 39.44 against MCF-7, ZR-75-1, MDA-MB-231; and 58 against MDM2; *Ki* = 0.74 ± 0.12 μM, respectivrly

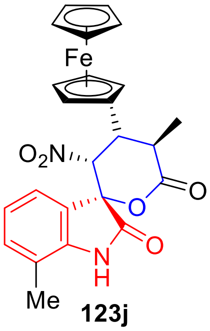

123j

% Inhibition at 1.0 μM = 42.42, 45.38, 34.86 against MCF-7, ZR-75-1, MDA-MB-231; and 42 against MDM2, respectivrly

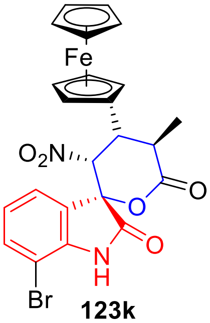

123k

% Inhibition at 1.0 μM = 41.34, 37.20, 31.98 against MCF-7, ZR-75-1, MDA-MB-231; and 39 against MDM2, respectivrly

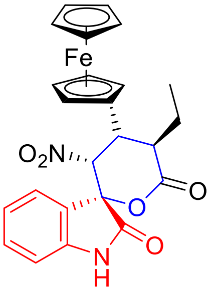

123l

% Inhibition at 1.0 μM = 42.77, 40.63, 37.13 against MCF-7, ZR-75-1, MDA-MB-231; and 47 against MDM2, respectivrly

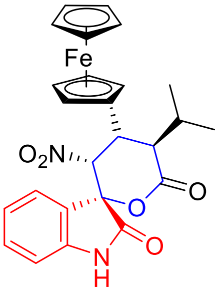

123m

% Inhibition at 1.0 μM = 46.55, 53.95, 31.36 against MCF-7, ZR-75-1, MDA-MB-231; and 49 against MDM2, respectivrly

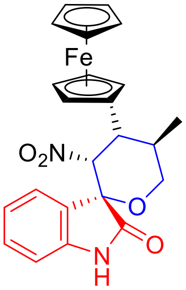

124a

% Inhibition at 1.0 μM = 33.95, 34.62, 23.89 against MCF-7, ZR-75-1, MDA-MB-231; and 35 against MDM2, respectivrly

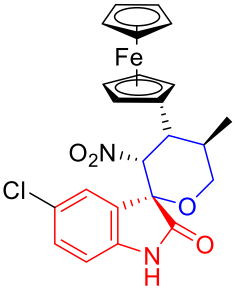

124b

% Inhibition at 1.0 μM = 44.10, 48.84, 29.82 against MCF-7, ZR-75-1, MDA-MB-231; and 42 against MDM2, respectivrly

Fig. S23 (continued)**.** Antiproliferation and MDM2 inhibitory properties of spirooxindole-pyrans **123–125**.

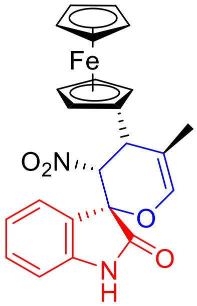

125a

% Inhibition at 1.0 μM
= 50.88, 51.38, 31.20
against MCF-7,
ZR-75-1, MDA-MB-231;
and 48 against MDM2,
respectivrly

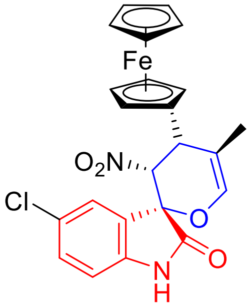

125b

% Inhibition at 1.0 μM
= 49.82, 47.82, 34.45
against MCF-7,
ZR-75-1, MDA-MB-231;
and 53 against MDM2,
respectivrly

Fig. S23 (continued)**.** Antiproliferation and MDM2 inhibitory properties of spirooxindole-pyrans **123**–**125**.

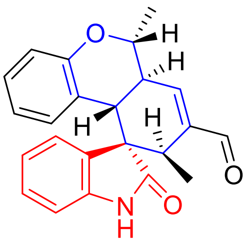

133a

% Inhibition = 23.5, 17.3 against A2870s, and A2870T at 30 μM

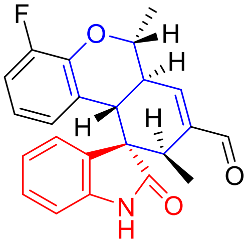

133b

% Inhibition = 22.2, 5.7 against A2870s, and A2870T at 30 μM

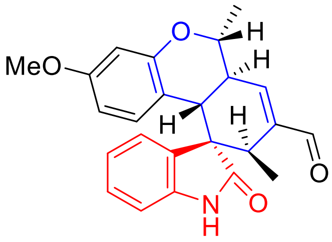

133c

% Inhibition = 9.3, 3.9 against A2870s, and A2870T at 30 μM

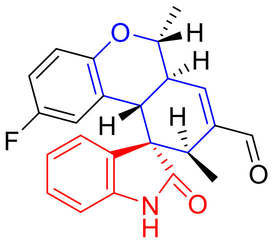

133d

% Inhibition = 22.7, 2.6 against A2870s, and A2870T at 30 μM

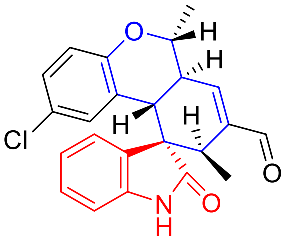

133e

% Inhibition = 16.5, 5.8 against A2870s, and A2870T at 30 μM

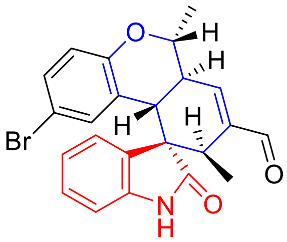

133f

% Inhibition = 35.9, 2.3 against A2870s, and A2870T at 30 μM

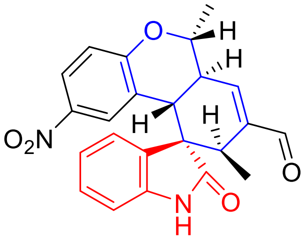

133g

% Inhibition = 25.2, 0.2 against A2870s, and A2870T at 30 μM

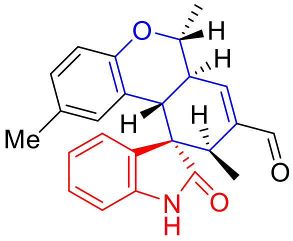

133h

% Inhibition = 28.2, 10.4 against A2870s, and A2870T at 30 μM

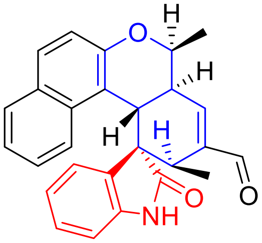

133i

IC_{50} = 7.9, 23.4, 9.5, 8.6, 18.3, 16.2, 13.7 μM against A2870s, A2870T, CT26, HCT116, A549, MCF7, and H1975, respectively

Fig. S24. Antiproliferation properties of spirooxindole-benzopyrans **133** and cisplatin

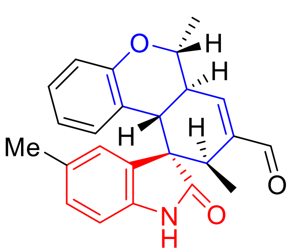

133j

% Inhibition = 12.8, 7.1 against A2870s, and A2870T at 30 μM

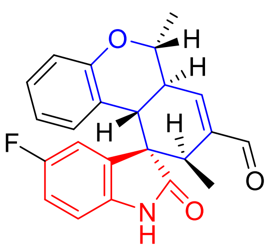

133k

% Inhibition = 14.5, 4.4 against A2870s, and A2870T at 30 μM

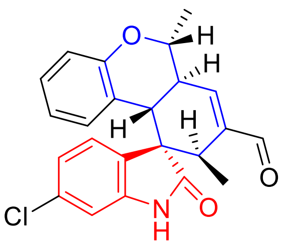

133l

IC_{50} = 19.7 μM against A2870s; % inhibition = 13.1 against A2870T at 30 μM

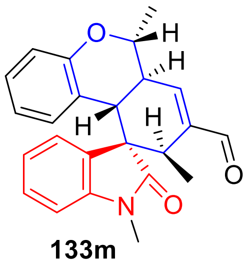

133m

% Inhibition = 26.3, 4.5 against A2870s, and A2870T at 30 μM

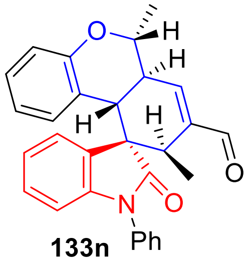

133n

IC_{50} = 18.9, 30, 25.7 μM against A2870s, H1299, and BGC823, respectively; % inhibition = 40.6 against A2870T at 30 μM

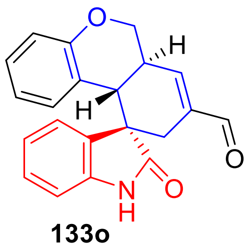

133o

IC_{50} = 21.6, 27.1, 25.8 μM against A2870s, CT26, and HCT116, respectively; % inhibition = 25.0 against A2870T at 30 μM

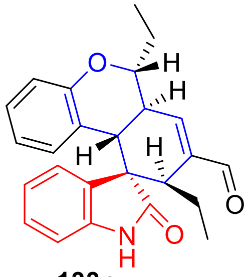

133p

% Inhibition = 34.1, 9.0 against A2870s, and A2870T at 30 μM

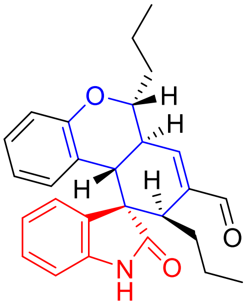

133q

IC_{50} = 25.7 μM against A2870s; % inhibition = 33.1 against A2870T at 30 μM

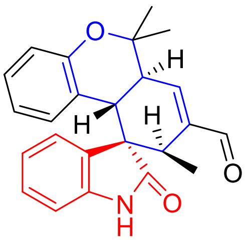

133r

% Inhibition = 14.4, 3.8 against A2870s, and A2870T at 30 μM

Fig. S24 (continued)**.** Antiproliferation properties of spirooxindole-benzopyrans **133** and cisplatin

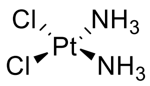

Cisplatin

IC_{50} = 20.9, 27.3, 8.9, 19.8, 23.7, 17.4, 15.6, 7.8, 9.7 μM against A2870s, A2870T, H1299, BGC823, CT26, HCT116, A549, MCF7, and H1975, respectively

Fig. S24 (continued)**.** Antiproliferation properties of spirooxindole-benzopyrans **133** and cisplatin

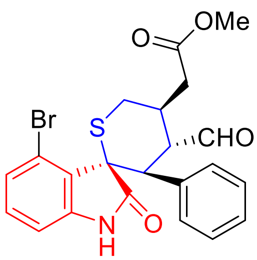

136a

IC_{50} = 35.14, 34.34, 21.34 μM against A549, HCT116, and MDA-MB-231, respectively

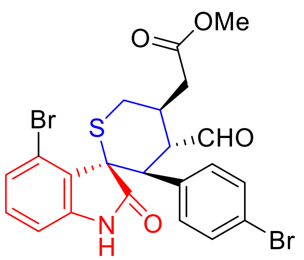

136b

IC_{50} = 1.67, 1.57, 3.55 μM against A549, HCT116, and MDA-MB-231, respectively

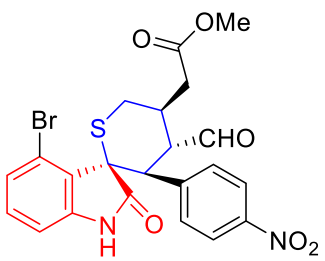

136c

IC_{50} = 30.82, 29.20, 20.92 μM against A549, HCT116, and MDA-MB-231, respectively

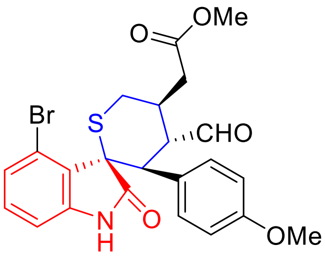

136d

IC_{50} = 34.86, 27.43, 24.91 μM against A549, HCT116, and MDA-MB-231, respectively

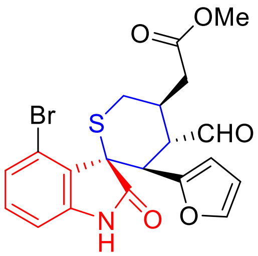

136e

IC_{50} = 30.92, 25.86, 19.39 μM against A549, HCT116, and MDA-MB-231, respectively

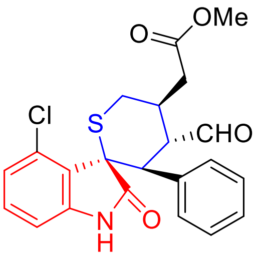

136f

IC_{50} = 12.55, 12.21, 13.77 μM against A549, HCT116, and MDA-MB-231, respectively

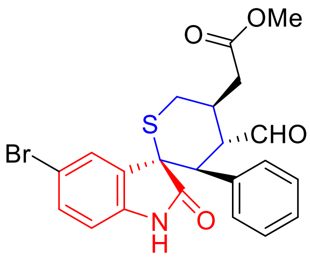

136g

IC_{50} = 12.26, 11.70, 12.72 μM against A549, HCT116, and MDA-MB-231, respectively

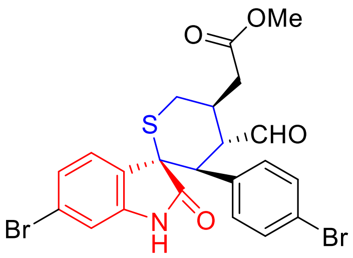

136h

IC_{50} = 6.20, 4.39, 2.82 μM against A549, HCT116, and MDA-MB-231, respectively

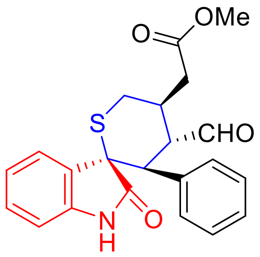

136i

IC_{50} = 86.40, 32.36, 29.88 μM against A549, HCT116, and MDA-MB-231, respectively

Fig. S25. Antiproliferation properties of spirooxindole-thiopyrans **136**, **137** and nutlin-3.

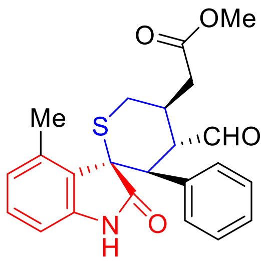

136j

IC_{50} = 13.82, 5.00, 11.65 μM against A549, HCT116, and MDA-MB-231, respectively

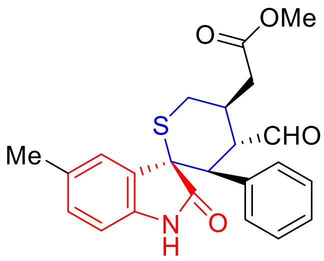

136k

IC_{50} = 17.15, 11.23, 21.12 μM against A549, HCT116, and MDA-MB-231, respectively

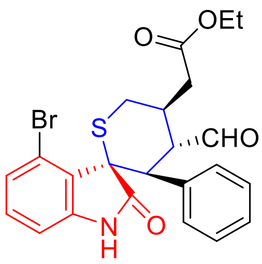

136l

IC_{50} = 25.67, 11.88, 12.37 μM against A549, HCT116, and MDA-MB-231, respectively

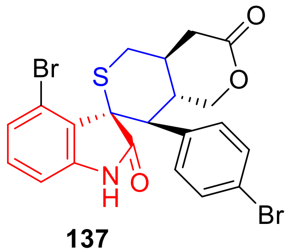

137

IC_{50} = 17.57, 13.37, 12.69 μM against A549, HCT116, and MDA-MB-231, respectively

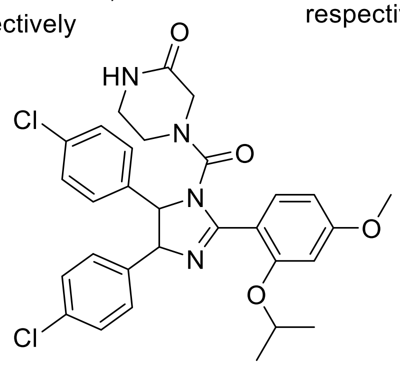

Nutlin-3

IC_{50} = 2.22, 1.16, 4.68 μM against A549, HCT116, and MDA-MB-231, respectively

Fig. S25 (continued)**.** Antiproliferation properties of spirooxindole-thiopyrans **136**, **137** and nutlin-3.

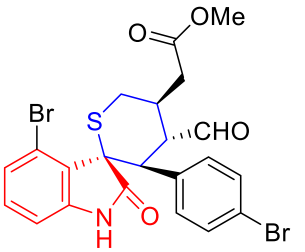

136

IC_{50} = 11.2 ± 0.8, 8.5 ± 0.7, 17.3 ± 2.2 μM against A549, MCF7, and HCT116, respectively; K_D = 4.9 ± 1.6 against MDM2

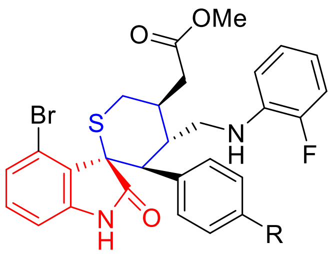

142a

IC_{50} = 4.2 ± 0.7, 3.6 ± 0.4, 10.5 ± 1.7 μM against A549, MCF7, and HCT116, respectively; K_D = 11.7 ± 4.4 against MDM2

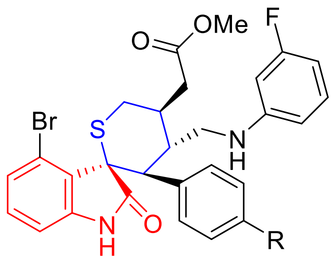

142b

IC_{50} = 3.5 ± 0.2, 2.8 ± 0.2, 8.6 ± 0.7 μM against A549, MCF7, and HCT116, respectively; K_D = 3.4 ± 0.6 against MDM2

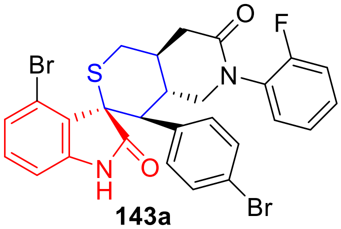

143a

IC_{50} = 2.3 ± 0.6, 4.6 ± 0.9, 9.3 ± 0.7 μM against A549, MCF7, and HCT116, respectively; K_D = 4.1 ± 1.2 against MDM2

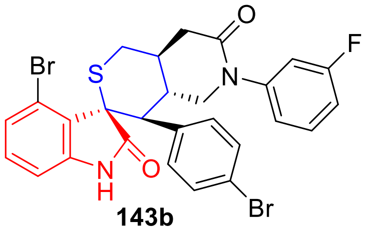

143b

IC_{50} = 3.5 ± 0.6, 3.8 ± 0.7, 9.5 ± 1.1 μM against A549, MCF7, and HCT116, respectively; K_D = 4.7 ± 0.3 against MDM2

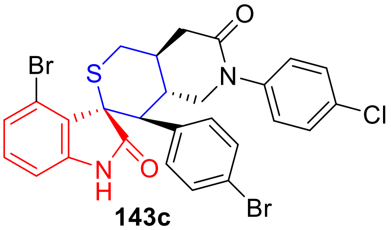

143c

IC_{50} = 4.8 ± 0.9, 4.2 ± 0.1, 8.0 ± 0.9 μM against A549, MCF7, and HCT116, respectively; K_D = 4.2 ± 0.9 against MDM2

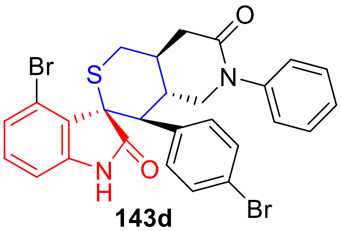

143d

IC_{50} = 4.2 ± 0.2, 4.0 ± 0.4, 8.6 ± 0.8 μM against A549, MCF7, and HCT116, respectively; K_D = 4.1 ± 0.8 against MDM2

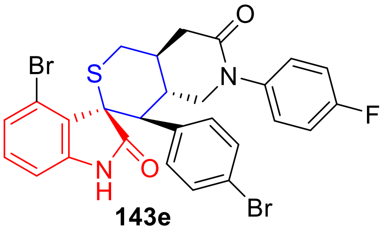

143e

IC_{50} = 4.4 ± 0.8, 4.0 ± 0.4, 10.5 ± 1.9 μM against A549, MCF7, and HCT116, respectively; K_D = 3.4 ± 0.2 against MDM2

Fig. S26. Antiproliferation and MDM2 inhibitory properties of spirooxindole-thiopyrans **136,** and **142–144**.

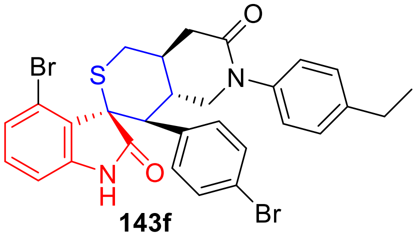

143f

IC_{50} = 2.6 ± 0.1, 3.2 ± 0.2, 10.4 ± 2.3 μM against A549, MCF7, and HCT116, respectively; K_D = 3.4 ± 0.3 against MDM2

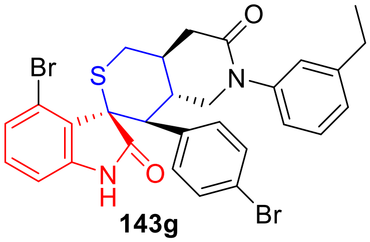

143g

IC_{50} = 4.5 ± 0.4, 3.6 ± 0.3, 8.5 ± 0.6 μM against A549, MCF7, and HCT116, respectively; K_D = 4.0 ± 0.7 against MDM2

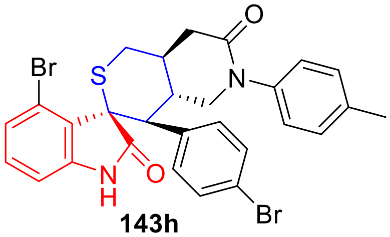

143h

IC_{50} = 4.6 ± 0.5, 3.0 ± 0.6, 5.1 ± 0.7 μM against A549, MCF7, and HCT116, respectively; K_D = 5.2 ± 1.8 against MDM2

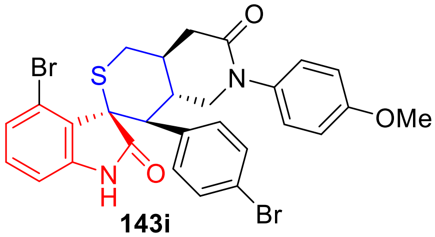

143i

IC_{50} = 4.3 ± 0.5, 3.0 ± 0.5, 8.3 ± 0.6 μM against A549, MCF7, and HCT116, respectively; K_D = 6.4 ± 0.7 against MDM2

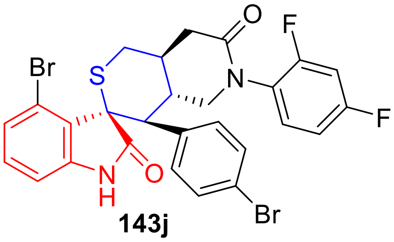

143j

IC_{50} = 2.5 ± 0.2, 4.4 ± 0.8, 9.8 ± 0.4 μM against A549, MCF7, and HCT116, respectively; K_D = 4.8 ± 1.1 against MDM2

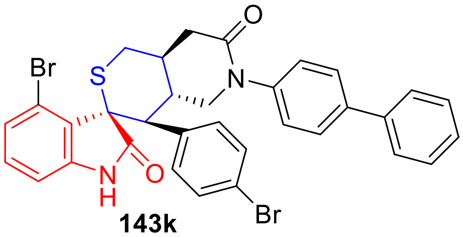

143k

IC_{50} = 4.1 ± 0.6, 6.9 ± 1.0, 5.6 ± 0.9 μM against A549, MCF7, and HCT116, respectively; K_D = 3.0 ± 0.6 against MDM2

Fig. S26 (continued)**.** Antiproliferation and MDM2 inhibitory properties of spirooxindole-thiopyrans **136,** and **142**–**144**.

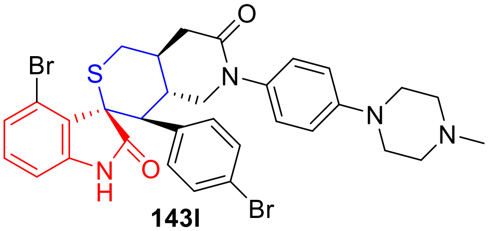

143l

IC$_{50}$ = 6.3 ± 1.2, 5.9 ± 1.3, 8.8 ± 1.5 μM against A549, MCF7, and HCT116, respectively; K_D = 3.0 ± 0.7 against MDM2

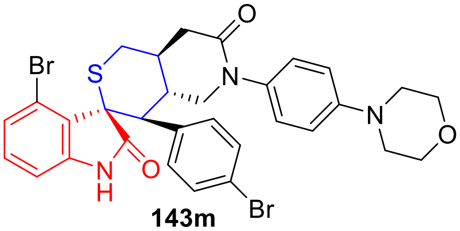

143m

IC$_{50}$ = 2.6 ± 0.4, 2.4 ± 0.4, 4.3 ± 0.6 μM against A549, MCF7, and HCT116, respectively; K_D = 2.0 ± 0.7 against MDM2

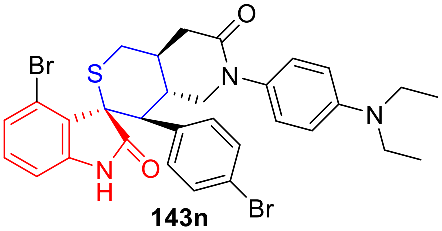

143n

IC$_{50}$ = 2.9 ± 0.2, 1.8 ± 0.5, 2.4 ± 0.1 μM against A549, MCF7, and HCT116, respectively; K_D = 4.2 ± 1.4 against MDM2

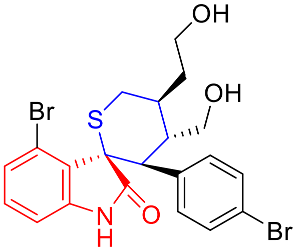

144

IC$_{50}$ = 16.5 ± 1.6, 13.5 ± 1.0, 19.1 ± 2.6 μM against A549, MCF7, and HCT116, respectively; K_D = 10.1 ± 2.6 against MDM2

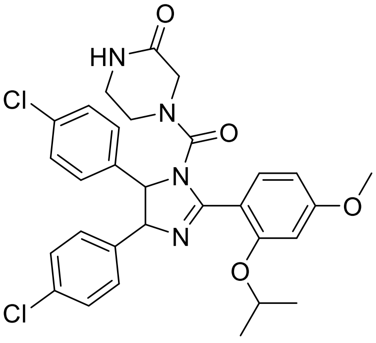

Nutlin-3

IC$_{50}$ = 10.0 ± 0.8, 14.9 ± 0.6, 28.4 ± 2.5 μM against A549, MCF7, and HCT116, respectively; K_D = 0.15 ± 0.04 against MDM2

Fig. S26 (continued)**.** Antiproliferation and MDM2 inhibitory properties of spirooxindole-thiopyrans **136,** and **142**–**144.**

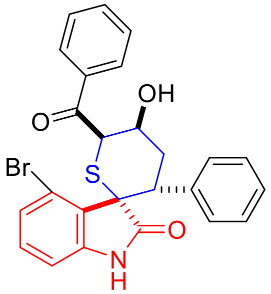

146a

% Inhibition = 64.9, 27.1, 11.6 against A549, MCF7, and HCT116 at 10 μM, respectively

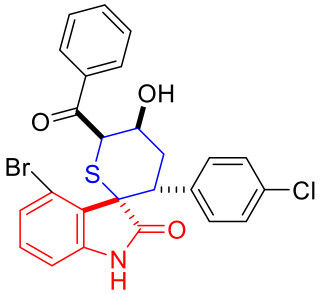

146b

% Inhibition = 36.8, 2.59, 19.7 against A549, MCF7, and HCT116 at 10 μM, respectively

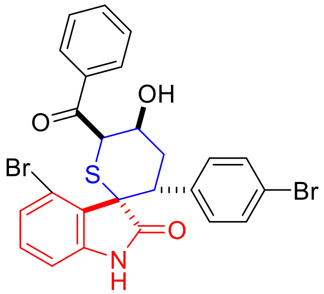

146c

% Inhibition = 74.9, 16.7, 48.3 against A549, MCF7, and HCT116 at 10 μM, respectively

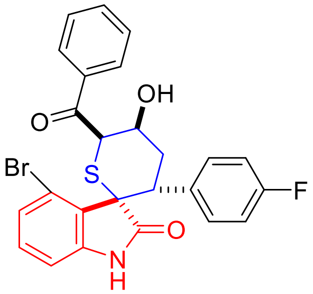

146d

% Inhibition = 79.8, 25.1, 36.3 against A549, MCF7, and HCT116 at 10 μM, respectively

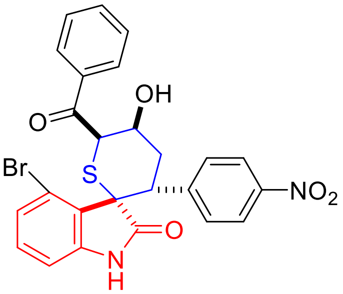

146e

% Inhibition = 60.7, 25.0, 45.1 against A549, MCF7, and HCT116 at 10 μM, respectively

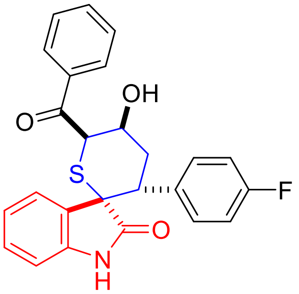

146f

% Inhibition = 42.5, 20.4, 26.8 against A549, MCF7, and HCT116 at 10 μM, respectively

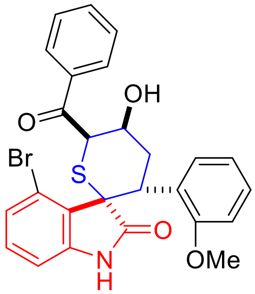

146g

% Inhibition = 69.0, 22.2, 48.3 against A549, MCF7, and HCT116 at 10 μM, respectively

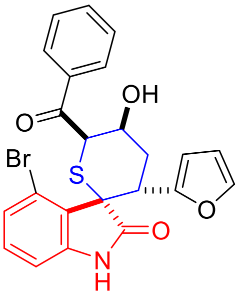

146h

% Inhibition = 65.0, 16.1, 11.5 against A549, MCF7, and HCT116 at 10 μM, respectively

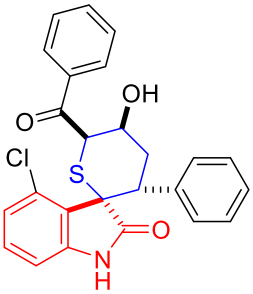

146i

% Inhibition = 67.4, 10.4, 27.0 against A549, MCF7, and HCT116 at 10 μM, respectively

Fig. S27. Antiproliferation properties of spirooxindole-thiopyrans **146–149** and nutlin-3.

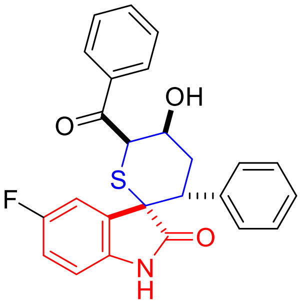

146j

% Inhibition = 56.7, 77.4, 43.7 against A549, MCF7, and HCT116 at 10 μM, respectively

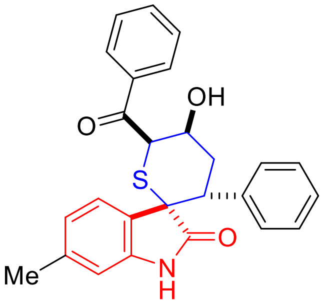

146k

% Inhibition = 79.3, 69.8, 47.2 against A549, MCF7, and HCT116 at 10 μM, respectively

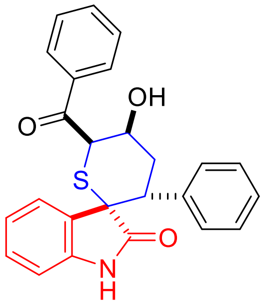

146l

% Inhibition = 24.5, 20.9, 14.8 against A549, MCF7, and HCT116 at 10 μM, respectively

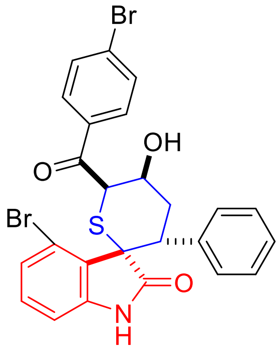

146m

% Inhibition = 86.9, 50.9, 81.7 against A549, MCF7, and HCT116 at 10 μM, respectively

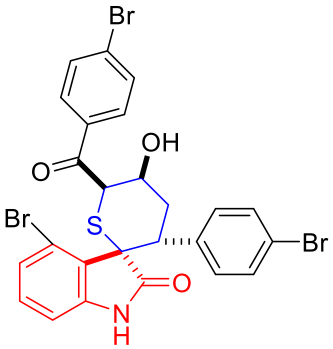

146n

% Inhibition = 50.7, 25.7, 59.9 against A549, MCF7, and HCT116 at 10 μM, respectively

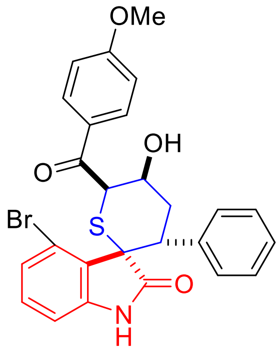

146o

% Inhibition = 38.4, 31.7, 15.9 against A549, MCF7, and HCT116 at 10 μM, respectively

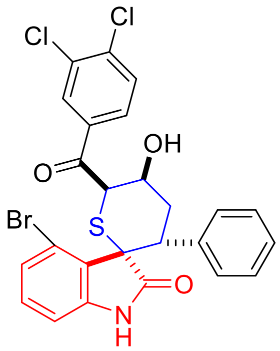

146p

% Inhibition = 82.2, 13.9, 81.9 against A549, MCF7, and HCT116 at 10 μM, respectively

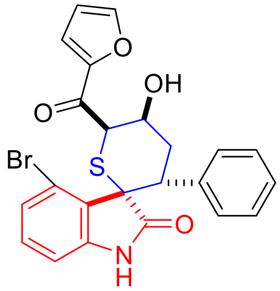

146q

% Inhibition = 52.1, 25.4, 22.1 against A549, MCF7, and HCT116 at 10 μM, respectively

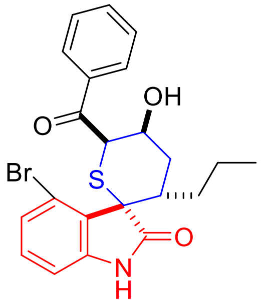

146r

% Inhibition = 55.3, 29.4, 52.2 against A549, MCF7, and HCT116 at 10 μM, respectively

Fig. S27 (continued)**.** Antiproliferation properties of spirooxindole-thiopyrans **146–149** and nutlin-3.

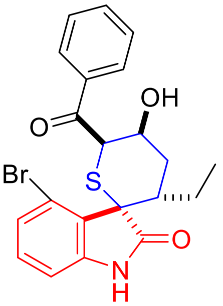

146s

% Inhibition = 53.2, 32.4, 56.3 against A549, MCF7, and HCT116 at 10 μM, respectively

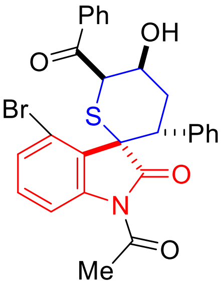

147

% Inhibition = 70.3, 77.6, 56.4 against A549, MCF7, and HCT116 at 10 μM, respectively

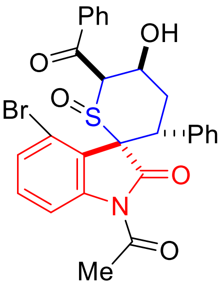

148

% Inhibition = 37.1, 43.1, 50.1 against A549, MCF7, and HCT116 at 10 μM, respectively

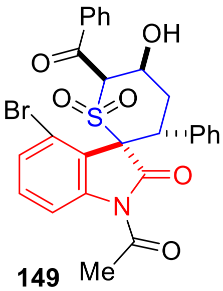

149

% Inhibition = 53.2, 68.8, 58.6 against A549, MCF7, and HCT116 at 10 μM, respectively

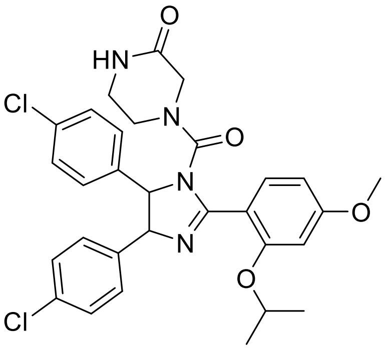

Nutlin-3

% Inhibition = 42.9, 33.1, 42.9 against A549, MCF7, and HCT116 at 10 μM, respectively

Fig. S27 (continued)**.** Antiproliferation properties of spirooxindole-thiopyrans **146**–**149** and nutlin-3.

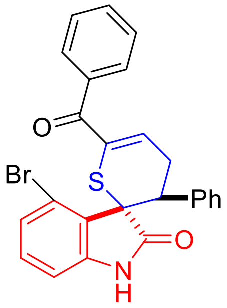

150a

% Inhibition = 78.5, 24.3, 45.0 against A549, MCF7, and HCT116, respectively

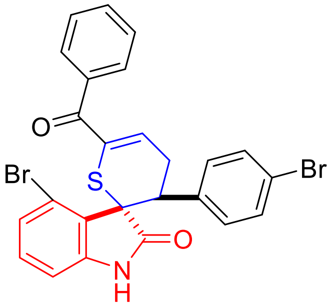

150b

% Inhibition = 88.1, 52.7, 86.0 against A549, MCF7, and HCT116, respectively

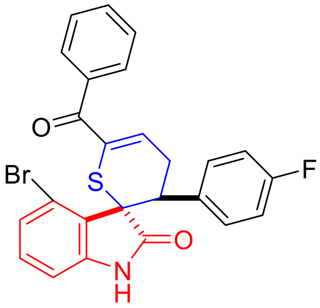

150c

% Inhibition = 84.4, 21.9, 44.1 against A549, MCF7, and HCT116, respectively

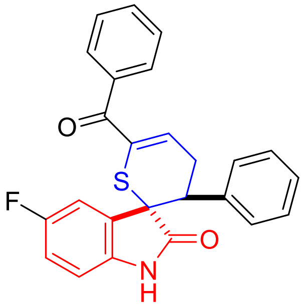

150d

% Inhibition = 74.2, 63.9, 88.4 against A549, MCF7, and HCT116, respectively

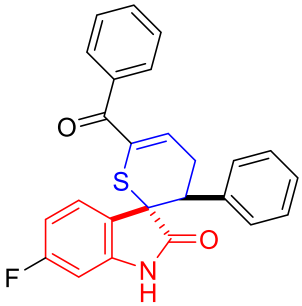

150e

% Inhibition = 66.0, 23.9, 45.1 against A549, MCF7, and HCT116, respectively

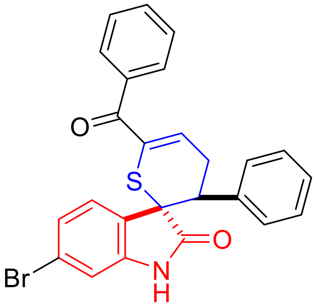

150f

% Inhibition = 67.6, 54.5, 80.0 against A549, MCF7, and HCT116, respectively

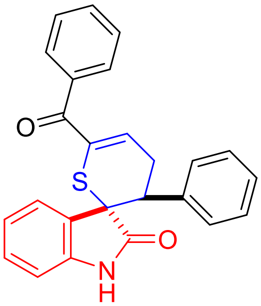

150g

% Inhibition = 57.6, 51.7, 87.6 against A549, MCF7, and HCT116, respectively

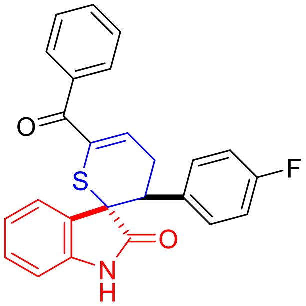

150h

% Inhibition = 72.3, 58.8, 91.1 against A549, MCF7, and HCT116, respectively

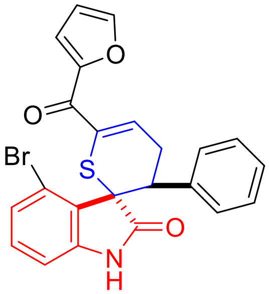

150i

% Inhibition = 87.1, 92.8, 92.1 against A549, MCF7, and HCT116, respectively

Fig. S28. Antiproliferation properties of spirooxindole-thiopyrans **150**, and nutlin-3.

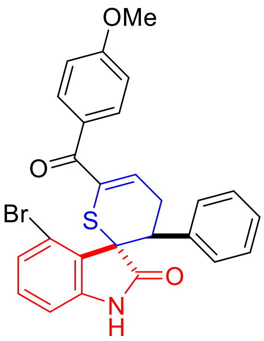

150j

% Inhibition = 74.3, 36.9, 68.4 against A549, MCF7, and HCT116, respectively

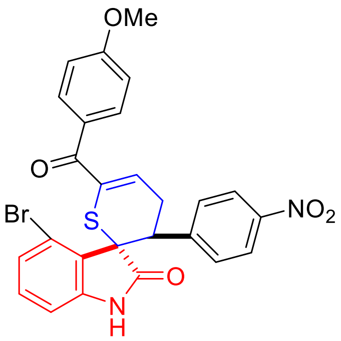

150k

% Inhibition = 73.9, 28.1, 48.1 against A549, MCF7, and HCT116, respectively

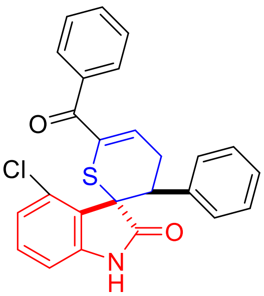

150l

% Inhibition = 65.4, 43.9, 48.8 against A549, MCF7, and HCT116, respectively

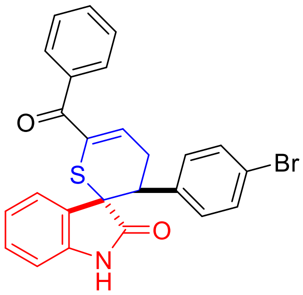

150m

% Inhibition = 75.1, 69.4, 91.9 against A549, MCF7, and HCT116, respectively

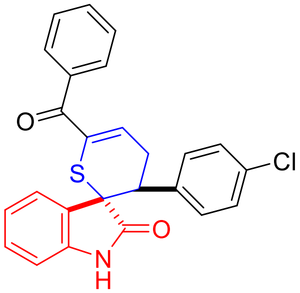

150n

% Inhibition = 72.5, 72.5, 89.1 against A549, MCF7, and HCT116, respectively

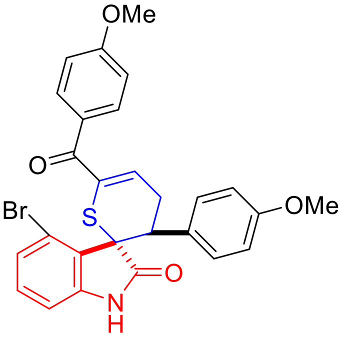

150o

% Inhibition = 40.9, 25.9, 14.3 against A549, MCF7, and HCT116, respectively

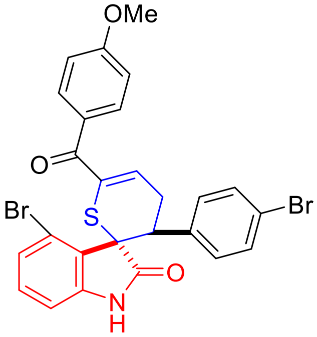

150p

% Inhibition = 19.0, 18.5, 10.8 against A549, MCF7, and HCT116, respectively

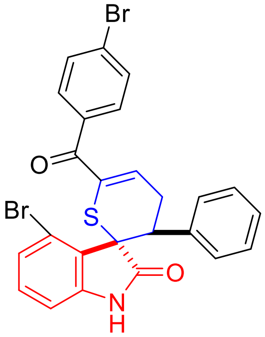

150q

% Inhibition = 93.1, 77.7, 67.3 against A549, MCF7, and HCT116, respectively

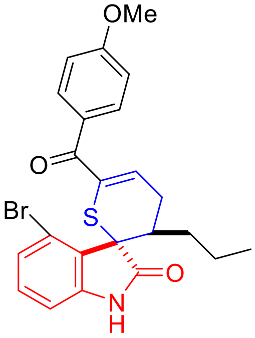

150r

% Inhibition = 44.0, 19.8, 22.7 against A549, MCF7, and HCT116, respectively

Fig. S28 (continued)**.** Antiproliferation properties of spirooxindole-thiopyrans **150**, and nutlin-3.

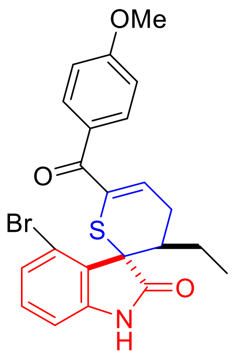

150s

% Inhibition = 46.9, 72.9, 70.8 against A549, MCF7, and HCT116, respectively

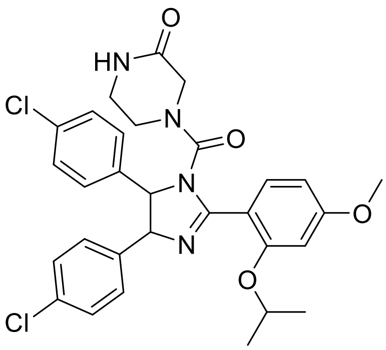

Nutlin-3

% Inhibition = 47.0, 32.8, 64.4 against A549, MCF7, and HCT116, respectively

Fig. S28 (continued)**.** Antiproliferation properties of spirooxindole-thiopyrans **150**, and nutlin-3.
